# Design and Synthesis of Lactams Derived from Mucochloric and Mucobromic Acids as *Pseudomonas aeruginosa* Quorum Sensing Inhibitors

**DOI:** 10.3390/molecules23051106

**Published:** 2018-05-07

**Authors:** Basmah Almohaywi, Aditi Taunk, Daniel S. Wenholz, Shashidhar Nizalapur, Nripendra N. Biswas, Kitty K. K. Ho, Scott A. Rice, George Iskander, David StC. Black, Renate Griffith, Naresh Kumar

**Affiliations:** 1 School of Chemistry, UNSW Australia, Sydney, NSW 2052, Australia; b.almohaywi@unsw.edu.au (B.A.); a.taunk@unsw.edu.au (A.T.); d.wenholz@unsw.edu.au (D.S.W.); s.nizalapur@unsw.edu.au (S.N.); nnathbiswas@gmail.com (N.N.B.); kitty.ho@unsw.edu.au (K.K.K.H.); g.iskander@unsw.edu.au (G.I.); d.black@unsw.edu.au (D.S.B.); 2 School of Pharmacy, King Khalid University, Abha 62529, Saudi Arabia; 3 The Singapore Centre of Environmental Life Sciences Engineering, Nanyang Technological University, Singapore 639798, Singapore; RSCOTT@ntu.edu.sg; 4 School of Medical Science, UNSW Australia, Sydney, NSW 2052, Australia; r.griffith@unsw.edu.edu.au

**Keywords:** quorum sensing, *Pseudomonas aeruginosa*, lactam, mucochloric acid, mucobromic acid

## Abstract

Bacterial infections, particularly hospital-acquired infections caused by *Pseudomonas aeruginosa*, have become a global threat with a high mortality rate. Gram-negative bacteria including *P. aeruginosa* employ *N*-acyl homoserine lactones (AHLs) as chemical signals to regulate the expression of pathogenic phenotypes through a mechanism called quorum sensing (QS). Recently, strategies targeting bacterial behaviour or QS have received great attention due to their ability to disarm rather than kill pathogenic bacteria, which lowers the evolutionary burden on bacteria and the risk of resistance development. In the present study, we report the design and synthesis of *N*-alkyl- and *N*-aryl 3,4 dichloro- and 3,4-dibromopyrrole-2-one derivatives through the reductive amination of mucochloric and mucobromic acid with aliphatic and aromatic amines. The quorum sensing inhibition (QSI) activity of the synthesized compounds was determined against a *P. aeruginosa* MH602 reporter strain. The phenolic compounds exhibited the best activity with 80% and 75% QSI at 250 µM and were comparable in activity to the positive control compound Fu-30. Computational docking studies performed using the LasR receptor protein of *P. aeruginosa* suggested the importance of hydrogen bonding and hydrophobic interactions for QSI.

## 1. Introduction

Exploring new directions to combat bacterial infections has become critically important with the rising incidence of hospital-acquired bacterial infections and the global prevalence of bacterial resistance. Traditional antibiotics are either bactericidal (kill bacteria) or bacteriostatic (inhibit the growth of bacteria) [1]. Therefore, the selective evolutionary pressures exerted by these antibiotics on microorganisms have resulted in the rise and spread of antibiotic resistance [2]. Other factors that have contributed to increased drug resistance include the expanded use of medical devices, treatments for infections in immune-compromised patients and the overuse or mishandling of antibiotics either intentionally or inadvertently [2]. Therefore, novel therapeutic approaches to combat bacterial infection and resistance are required [3].

**Figure 1:**
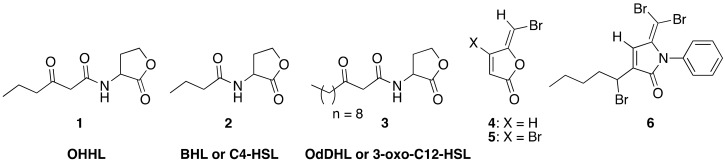
Bacterial *N*-acylated homoserine lactones (AHLs) used for QS and synthetic QS inhibition.

Bacteria possess an adaptive intracellular mechanism that aids in their communications in a cell density-dependent manner, allowing them to synchronize gene expression as a group using a process termed quorum sensing (QS) [4]. Bacteria sense changes in their population density via the production of diffusible small molecules known as autoinducers, such as the *N*-acylated homoserine lactones (AHLs) *N*-(3-oxohexanoyl)-l-homoserine lactone (OHHL) **1** in *Vibrio fischeri*, and *N*-butyryl-l-homoserine lactone **2** (C4-HSL, also known as BHL) and *N*-(3-oxododecanoyl)-l-homoserine lactone **3** (OdDHL, also known as 3-oxo-C12-HSL) in *P. aeruginosa* (Figure 1) [5].

*P. aeruginosa* is an opportunistic and ubiquitous human pathogen and is amongst the most common causative agents of nosocomial and life-threatening infections [6]. In *P. aeruginosa*, QS is coordinated via a triumvirate of *LuxR* homologues, namely the LasR, RhlR and QscR systems [7]. These overlapping receptors have significant roles in the regulation of gene expression and QS signals. In *P. aeruginosa*, QS mediates and controls the gene expressions and phenotypes responsible for its pathogenicity and resistance against the host immune system. It does so by utilizing autoinducers, which trigger the production of virulence factors (e.g., elastase, protease, pyocyanin) and biofilm formation. These phenotypes, however, are not vital to the growth of this pathogen, and thus, their inhibition does not have bacteriostatic or bactericidal effects. Hence, the interference with and antagonism of QS comprise an attractive strategy to overcome and prevent virulence and pathogenicity with minimal likelihood of resistance. Antagonists possessing the lactone head of natural AHLs, but with non-native acyl chains, represent the most extensively-studied class of synthetic QS modulators. For instance, synthetic analogues of the natural autoinducer OdDHL **3** were developed as AHL-based LasR antagonists (Figure 1) [8]. However, QS antagonists derived directly from AHLs are sensitive to enzymatic and chemical hydrolysis of the lactone ring at physiological pH, giving ring-opened products that lack QS activity [9]. Hence, several research groups have investigated replacement of the lactone group with saturated or unsaturated cyclic and heterocyclic structures [9,10].

Our research group has led the development of both fimbrolide-based analogues [11] and their lactam analogues [12,13]. The lactam fimbrolide analogue **6** exhibited good QS activity and was the most active lactam-based fimbrolide derivative tested against AHL-mediated signaling in *Escherichia coli* [13]. In line with our continuing efforts to develop new QS inhibitors, we explored the potential use of mucochloric acid **7** and mucobromic acid **8** as precursors that could provide access to functionalized lactams [14]. These compounds are inexpensive, commercially available, highly functionalized and possess multiple sites for reactivity, particularly the two halogen atoms situated across one double bond adjacent to a pseudo acid functionality. Mucohalic acids have been used for the synthesis of furanones with antibacterial and antibiofilm activities [15,16], anticancer activity [17] and anti-inflammatory activity [18]. Mucohalic acids have also been used for the synthesis of furanone-based natural products such as rubrolide [19,20], as well as their lactam analogues, showing herbicidal [20] and antibiofilm activities [15]. They have also been used as precursors of the antiseizure agent levetiracetam [21]. In this work, a library of 34 lactam compounds was prepared using the reductive amination of mucochloric and mucobromic acids with selected aliphatic and aromatic amines to furnish *N*-alkyl- and *N*-aryl 3,4 dichloro- and 3,4-dibromopyrrole-2-one derivatives. The QS inhibitory activities of these lactam derivatives were determined.

## 2. Results

### 2.1. Chemical Synthesis

#### 2.1.1. Synthesis of *N*-alkyl and *N*-aryl Lactams

**Scheme 1:**
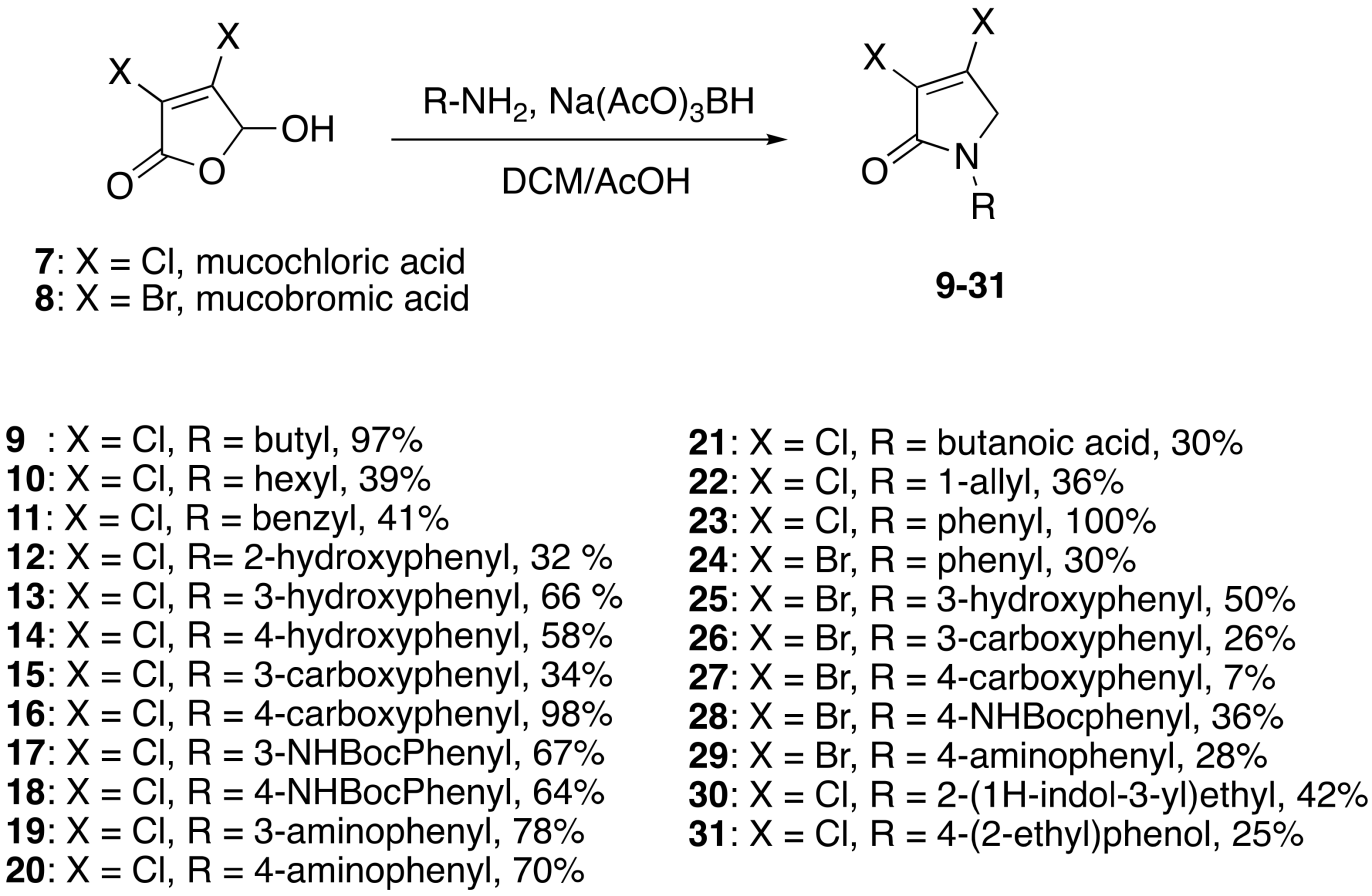
Reductive amination of mucochloric acid **7** and mucobromic acid **8** to generate lactam Compounds **9**–**31**.

In order to generate a diverse array of lactam analogues, various amines were selected including aliphatic, arylated and heterocyclic amines. Lactams **9**–**31** were prepared following a literature method using sodium triacetoxyborohydride as a reducing agent in acetic acid and mucohalic acids (mucochloric acid **7** and mucobromic acid **8**) (Scheme 1) [14]. Most of the products were purified easily by either trituration from methanol or flash column chromatography (ethyl acetate/hexane) (if required). Compounds with a relatively acidic functional group, such as those derived from carboxyaniline (**15**–**16)** or aminophenol (**12**–**14**), precipitated out from the reaction mixture as pure solids. Products were obtained in reasonable yields. The yields obtained are shown in Scheme 1 and were dependent on both the identity of the starting mucohalic acid and the type of functional group introduced. In general, reactions with mucochloric acid gave higher yields compared to those with mucobromic acid. The proposed mechanism for this reaction [14] depends on nucleophilic attack of the amine onto a protonated carbonyl group. The higher electrophilicity of chlorine compared to bromine facilitates this. When aliphatic groups were introduced, butylamine produced a higher percentage yield (79%) for **9** compared to hexylamine (39%) for **10**. In the phenolic Compounds **12**–**14**, the yield was lower when the hydroxyl group was installed on the *ortho* position compared to the *meta* and *para* analogues. The yields for these phenols follow the relative nucleophilicity of the nitrogen.

In the *ortho* derivative, the internal hydrogen bond resulted in the lower nucleophilicity of **12**, which required longer reaction times. The introduction of *para*-carboxyphenyl (**16**) resulted in a higher yield compared to the *meta* analogue **15**, suggesting the impact of the position of the electron-withdrawing group on the nucleophilicity of the aniline group. However, in the reaction of mucobromic acid with carboxy substituted amines, the *meta*-carboxyphenyl (**26**) produced a relatively higher yield than the *para* analogue (**27**) of 26% and 7%, respectively. Furthermore, the formation of the *para* analogue (**27**) required more forcing reaction conditions by heating at 30 °C instead of room temperature. Compounds **19** and **20**, and their mucobromic analogue **29**, containing aminophenyl groups, were prepared from protected *meta-* and *para*-phenylenediamines via the Boc Compounds **17**, **18** and **28**.

Products were fully characterized using ^1^H and ^13^C NMR spectroscopy and high-resolution mass spectrometry. Compounds **7**–**31** could possibly undergo nucleophilic substitution at the vinylic carbon with the loss of a chlorine or bromine. However, high-resolution mass spectrometry confirmed the correct mass to charge ratio of the anticipated products without the loss of halogens. In ^1^H NMR (in DMSO-*d*_6_), the loss of the 5-CH and 5-hydroxyl groups, which resonate at 6.24 ppm and 8.63 ppm, respectively, for mucochloric acid and 6.21 ppm and 8.52 ppm, respectively, for mucobromic acid confirmed the disappearance of the starting materials. Likewise, ^13^C NMR spectra showed the disappearance of the C5 signal, which resonates at 96.76 ppm and 100.13 ppm in mucochloric and mucobromic acids, respectively. The ^1^H NMR spectra of the products showed a distinctive singlet peak for the ring C5 methylene (CH_2_) protons. For compounds derived from aliphatic amines including the butyl **9**, hexyl **10** and allyl **22** analogues, this peak appeared at 4.01–4.03 ppm. In the tryptamine-derived Compound **30**, the methylene peak appeared as a singlet at 4.30 ppm (in DMSO-*d*_6_). The methylene peak of the tyramine-derived Compound **31** (in CDCl_3_) resonated upfield at 3.81 ppm, presumably due to an electron-donating ability of these analogues. In contrast, compounds with an aromatic substituent (e.g., **12**–**20**, **23**–**29**) exhibited the C5 methylene protons at 4.6–4.8 ppm due to the deshielding effect of the aromatic group. The hydroxyl proton of the phenolic Compounds **12**–**14** was observed as a singlet at 9.5–9.8 ppm. The correct numbers of carbons were observed for Compounds **9** to **31** as assigned by ^13^C NMR. Differences between the *N*-alkyl and *N*-aryl lactams **9**–**31** were also observed in the distinctive carbon C5 peaks. The C5 of the alkyl compounds resonated at 52.9 ppm, except for the *N*-allyl, which resonated at 53.4 ppm due to the subtle deshielding effect of the allylic double bond. Compounds with *N*-aryl substituents were also characterized by their C5 peak, which resonated downfield at 53.4 ppm–55.4 ppm. Substituents on the *N*-phenyl had an effect on the resonance of C5. For example, the C5 in Compound **23** (*N*-phenyl) resonated at 53.4 ppm, while installing the hydroxyl group at the *ortho* position of the *N*-phenyl resulted in a chemical shift of 55.4 ppm (Compound **12**), compared to 54.0 ppm for Compound **13** (*N*-3-hydroxyphenyl) and 54.5 ppm for Compound **14** (*N*-4-hydroxy phenyl). Their mucobromic analogues **24** (*N*-phenyl) and **25** (3-hydroxyphneyl) had a chemical shift of 57.4 ppm (supplementary materials NMR Spectra).

#### 2.1.2. Synthesis of Amide Analogues

**Scheme 2:**
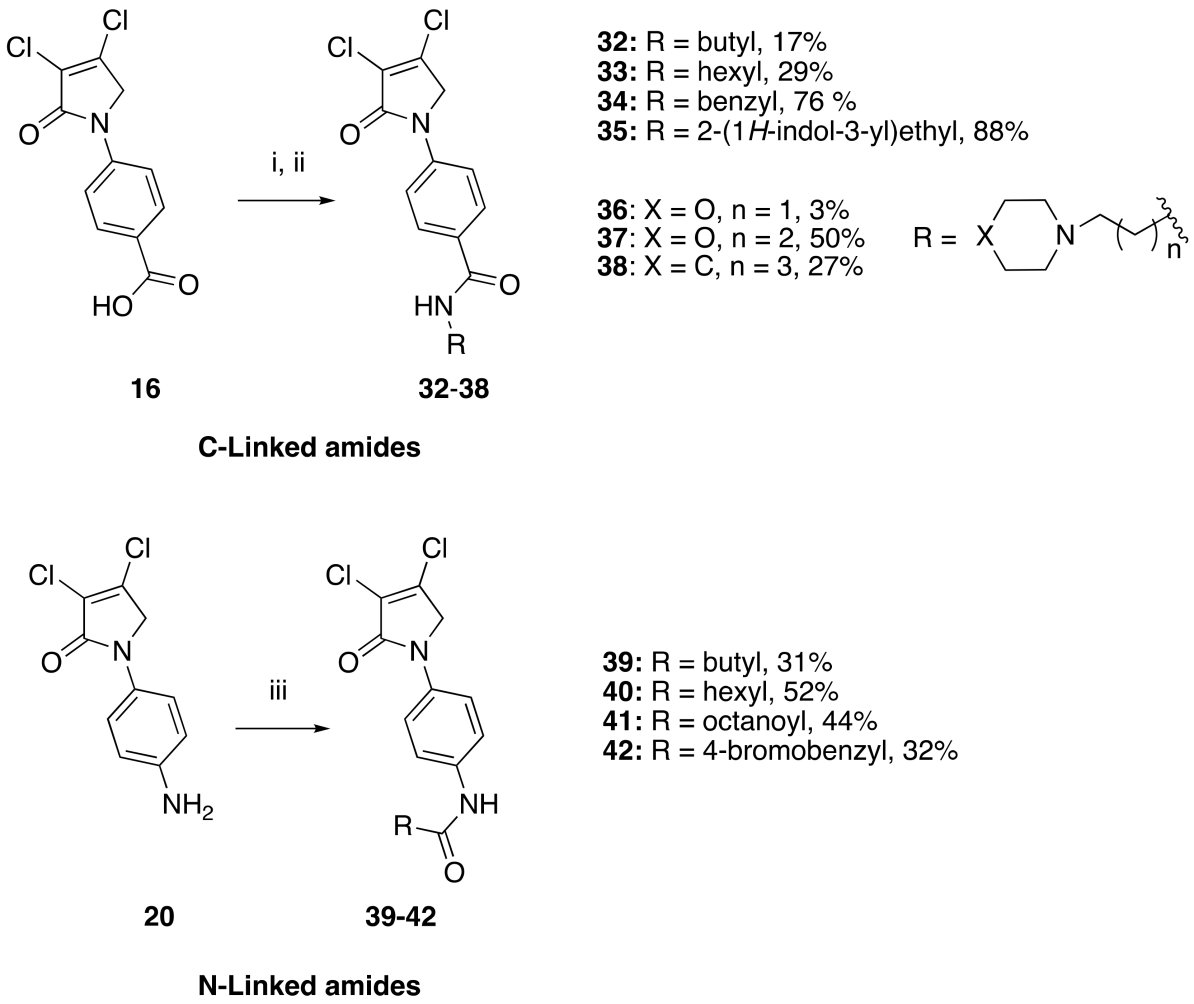
Reductive amination of mucochloric acid **7** and mucobromic acid **8** to generate lactam Compounds **9**–**31**. Reagent and conditions: I, SOCl_2_; ii, R-NH_2_, TEA, rt, THF, 24 h; iii, R-COCl, THF, TEA, rt, 24 h.

Additional amide groups were introduced into the lactam compounds to elaborate the structure-activity relationship (SAR) surrounding these compounds. We envisaged that the amide functionality might offer additional capacity to form hydrogen bonds with the biological target, the LasR receptor protein, and thus enhance quorum sensing inhibition (QSI). Therefore, the C-linked amides **32**–**38** and the *N*-linked amides **39**–**42** were targeted (Scheme 2). Mucochloric acid derivatives **16** and **20** were selected for the synthesis of the amides due to their high yields and ease of synthesis. To generate the C-linked amides, various amide coupling reagents such as 1-Ethyl-3-(3-dimethylaminopropyl)carbodiimide (EDC)/hydroxy benzotriazole (HOBT), *N,N*-diisopropyl ethylamine (DIPEA) and hexafluorophosphate benzotriazole tetramethyl uronium (HBTU) were used to couple the 4-carboxylphenyl mucochloric acid derivative **16** with various aliphatic amines; however, low yields of the products were obtained. Therefore, the acid **16** was instead converted to the corresponding acid chloride using SOCl_2_ and then reacted with the corresponding amines in the presence of triethylamine to give the target Compounds **32**–**38** in variable yields (Scheme 2). Difficulties in the purification of the products were the main cause for the lower yields.

In the ^1^H NMR, the C5 methylene group was observed as a singlet at 4.88–4.91 ppm, and there were no significant changes in this peak for the C-linked amides **32**–**38** when compared with the precursor acid (Compound **16**). The NH peak of the amides was observed as a singlet at 8.41 and 8.43 ppm for Compounds **32** and **33** containing an aliphatic chain, respectively. The amide peak of Compound **34** was observed, but overlapped with the aromatic protons at 7.21–7.46 ppm, and the NH peak of Compound **35** was observed at 10.94 ppm, presumably due to the electron-withdrawing effect of the indole moiety. The amide peak of Compounds **36** and **37** appeared as a broad signal at 8.87 and 7.91 ppm under the aromatic protons. For Compound **38**, the NH peak was observed upfield as a singlet at 5.3 ppm. Attempts to prepare amides from the acid chloride derivative of **16** with 2-aminothiophene, 2-aminobenzothizole, 2-aminobenzimidazole, 2-aminobenzoxazole and *meta*-aminopyridine were unsuccessful, giving either unreacted starting materials or intractable mixtures of products. It is possible a stronger base is needed to overcome the reduced nucleophilicity of the heterocyclic amines. To generate *N*-linked amide analogues **39**–**42**, intermediate **20** (Scheme 2) was acylated with the appropriate acyl chloride derivatives resulting in moderate yields. The acid chloride compounds were either commercially available or made by treating the corresponding acid with thionyl chloride.

The completion of the reaction was confirmed with the disappearance of amine peak at 5.13 ppm and the appearance of amide peaks at 9.92, 7.18, 7.24 and 10.33 ppm for Compounds **39**–**42** with butyl, hexyl, octyl and 4-bromobenzyl groups, respectively.

### 2.2. Biological Activity of Lactam Compounds against P. aeruginosa

#### 2.2.1. QSI of Alkyl and Aryl Lactams

The synthesized lactams were evaluated for their QSI activity following an established literature protocol [22]. In this assay, the *P. aeruginosa* MH602 (PAMH602) strain that expresses the *luxR* gene and the *luxI* gene and a promoter fused to the green fluorescent protein (GFP) reporter gene were used. Hence, the level of GFP fluorescence is a measure of AHL-mediated QS in this *P. aeruginosa* strain. The PAMH602 culture was incubated with varying concentrations (62.5 µM, 125 µM and 250 μM) of the synthesized compounds at 37 °C for 15 h, and the fluorescence of GFP at λ = 535 nm was determined. The optical density (OD) of the cultures at 600 nm was also measured to assess the effect of the compounds on bacterial growth. In these experiments, the halogenated furanone (**5**) (Fu-30) and another known QS antagonist (TP-5) were used as positive controls. The effects of the compounds on GFP fluorescence and OD are presented in Table 1.

The QS inhibition assay indicated that the synthesized lactams displayed promising QSI activity. Compounds **9**, **12**–**13**, **19**, **22**–**24** and **30** exhibited the highest QSI of 71.4–83.0% at 250 µM. The most active compound, the *ortho*-hydroxyphenyl mucochloric analogue **12**, reduced GFP fluorescence by 83% (±2.8) at 250 µM and was comparable in potency to the positive control **5** and better in activity than the triphenyl antagonist (TP-5). The QSI activity of **12** was well maintained at lower concentrations, with 82.0% (±2.6) and 69.4% (±0.3) inhibitions at 125 µM and 62.5 µM, respectively. There was a slight reduction in activity although not significant (78 ± 2.3% at 250 μM, *p* > 0.05) as the phenol group moved to the *meta* position as in derivative **13**. The *para*-phenolic lactam **14** was the least active (58.0 ± 3.3% at 250 μM, *p* < 0.001) when compared to its *ortho* (**12**) and *meta* (**13**) counterparts, suggesting the importance of the position of the phenolic hydroxyl group on activity. Interestingly, no significant difference (*p* > 0.05) in activity was observed when comparing Compound **12** with other potent *N*-aryl analogues including Compound **19** (80.4 ± 1.1%) and Compound **23** (81.7 ± 0.1%) containing the *N*-3-aminophenyl and *N*-phenyl groups, respectively, which both exhibited high inhibition at 250 µM. Furthermore, the activity of Compounds **19** and **23** was also retained at lower concentrations, giving a high percentage of inhibition of 78.1% (±1.6) and 76.5% (±0.4) at 62.5 µM, respectively.

Compound **20** containing a 4-aminophenyl group was less active compared to the *meta*-amino phenyl analogue **19**. These observations from both the *N*-phenolic and the *N*-aminophenyl compounds indicate the importance of the position of the electron donating groups installed on the *N*-phenyl ring of the lactam, with both *para*-substituted compounds showing reduced activity. Activity was also retained with the mucobromic analogue **24** (80.7 ± 3.0% at 250 µM), suggesting that both of the dichloro- and dibromo-pyrrolone were acceptable for activity for this type of scaffold. The *N*-carboxyphenyl lactams including Compounds **15**–**16** and Compound **21** (*N*-butanoic acid group), both derived from mucochloric acid, were less active compared to other *N*-aryl lactams. The position of the carboxyl group did not improve the activity, and the same was observed with their mucobromic analogues **26** and **27**, which were even less active. 

The *N*-alkyl compounds were relatively less active compared to the *N*-aryl compounds. Compounds **9** and **22** containing *N*-butyl and *N*-allyl groups possessed QSI activity (71.4 ± 1.0% and 76.4 ± 1.2% at 250 µM, respectively), but their activity was poor at lower concentrations. Moreover, adding two carbon atoms to the butyl chain of Compound **9** to give the *N*-hexyl lactam **10** showed a reduction in activity (68.0 ± 0.5% at 250 µM). Compound **30**, containing the *N*-2-(1*H*-indol-3-yl)ethyl substituent had QSI activity (74 ± 1.7% at 250 μM), but the activity dropped at lower concentrations (32.1 ± 3.5% at 62.5 µM).

#### 2.2.2. QSI of C-Linked and N-Linked Amide Analogues

In general, amides **32**–**42** displayed only low to moderate activity, as their QSI activity reduced by 30–54% at 250 μM, with low to no activity at 62.5 µM. The C-linked amides **32**–**38** were generally more active than the *N*-linked amides **39**–**42**. This indicates the possible importance of the position or the orientation of the amide group. The C-linked amides had higher activity, which ranges from 30–53.5% inhibition at 250 µM when compared to their parent acid lactam **16** (QSI = 19.8% ± 3.3 at 250 µM). Compounds **32** and **34** had a moderate QSI of 52.5% (±6.2) and 54.2% (±5.9), respectively. A comparison of the *N*-linked amides to their parent 4-aminophenyl lactam **20** (QSI = 58.8% ± 5.8 at 250 µM) showed reduced QSI activity of these amides, which ranges from 53.6–34.5%. The octyl group produced inhibition of 53.6% (±3.1), which was relatively higher than the butyl and hexyl groups.

#### 2.2.3. Evaluation of Growth Inhibition

To ensure that the decrease in GFP fluorescence was related to QS inhibition and not a result of toxicity or a decline in the population of the bacteria, the OD of the cultures were also measured and the degree of growth inhibition noted along with the QS inhibition data in Table 1 (full results are presented in the Supplementary Information). Overall, our synthesized library of lactams displayed low growth inhibition against *P. aeruginosa*. At 62.5 µM, all synthesized compounds had little to no effect on the growth of bacteria. Although the synthesized compounds have minimal effect on bacterial growth, it should be noted that there may be other factors that can affect the QS activity [23]. The best compounds in this study and TP-5 reduced bacterial growth by less than 30%, whereas the positive control compound Fu-30 (**5**) inhibited *P. aeruginosa* growth by 84% at 250 µM. Therefore, this study showed that the tested compounds inhibit QS with minimal effect on the growth of *P*. *aeruginosa*.

#### 2.2.4. Pyocyanin Inhibition

**Figure 2:**
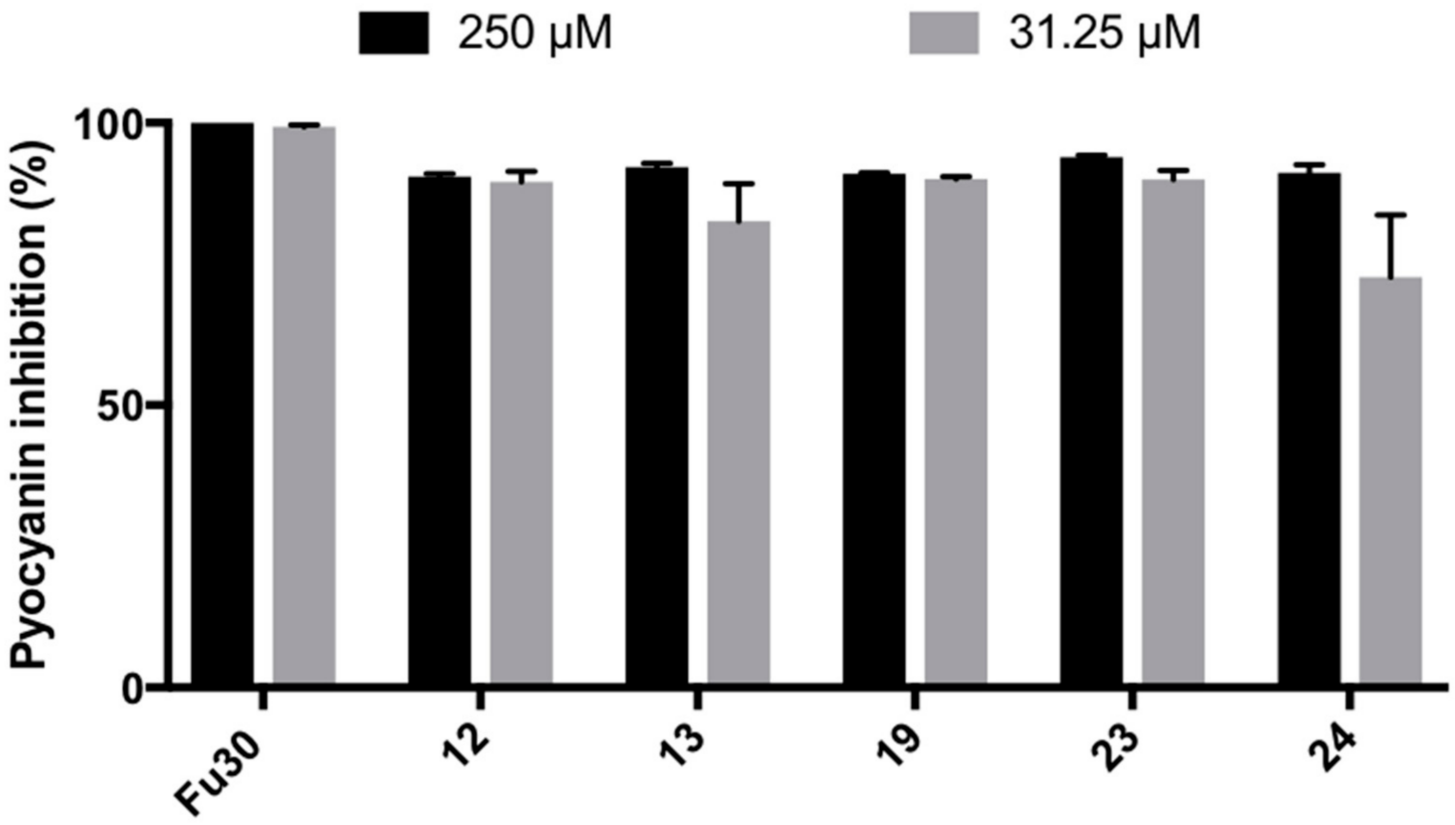
Pyocyanin inhibition in *P. aeruginosa* PAO1 by the synthesized compounds. Compounds were added to the bacteria at 250 μM or 31.25 µM and were incubated for 17 h with shaking. Pyocyanin production was measured at 695 nm in cell-free culture fluids. Error bars represent the standard error of two independent experiments.

The virulence factor pyocyanin is produced when cell density is high in response to the AHL molecule interacting with LasR. Since LasR is one of the determinant factors for the production of pyocyanin in *P. aeruginosa* [24], the ability of our compounds to inhibit pyocyanin production was investigated. Wild-type *P. aeruginosa* (PAO1) were grown in the presence of Compounds **12**, **13**, **19**, **23** and **24**, and the amount of pyocyanin in the culture supernatants was quantified based on its absorbance at 695 nm following a reported protocol [25]. The ability of the compounds to reduce pyocyanin levels was determined with respect to the levels of pyocyanin in the DMSO-treated positive control (Figure 2). Growth inhibition at OD_600_ was monitored, and the compounds showed moderate reduction in bacterial growth (22–30%).

The compounds were effective in inhibiting pyocyanin in the range of 90–94% at 250 µM. The compounds also showed potent activity at a lower concentration of 32 µM (80–89% inhibition). Our QS-based inhibitors showed comparable pyocyanin inhibition activity to those reported by others [24]. The outcome of this assay correlates well with the potent QS activity of these compounds.

### 2.3. Docking Studies

Lactams **9**–**42** were docked into the binding site of an X-ray crystal structure of the LasR receptor protein (supplementary materials Table S2) in complex with OdDHL (PDB Code 2UV0) using the Genetic Optimisation for Ligand Docking (GOLD) algorithm through the Accelrys Discovery Studio software package. Before docking, the compounds were minimized using a CHARMM force field. The crystal structure consisted of four subunits (two sets of dimers); however, compounds were only docked into the binding site of subunit E following the results of previous control dockings. The co-crystallised OdDHL ligand (**3**) was docked back into the protein giving an acceptable root mean square of deviation (RMSD) of 0.94 Å (heavy atoms) with the X-ray crystal structure.

**Figure 3:**
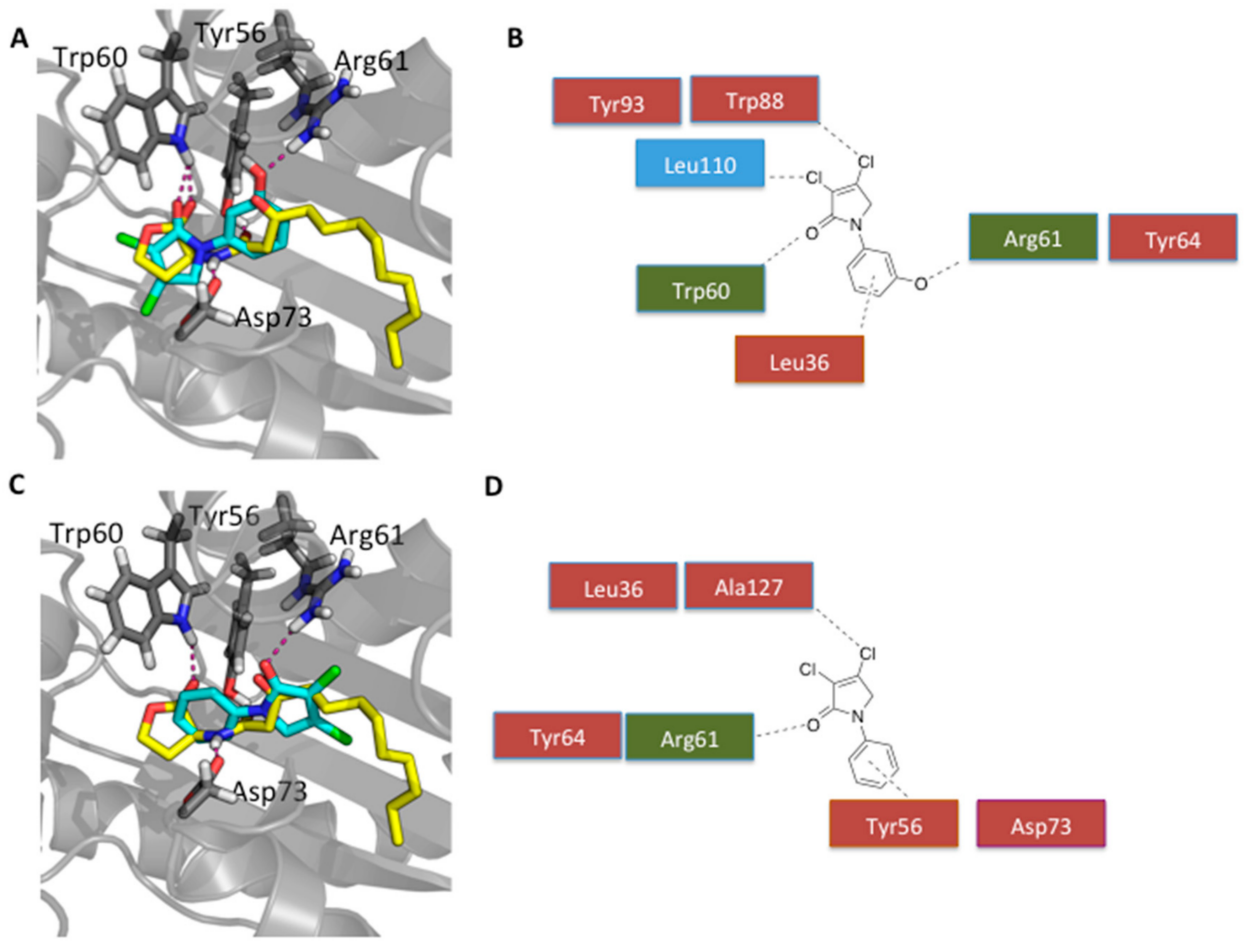
Predicted interactions for ligands **13** and **23** in the binding pocket of LasR compared to *N*-(3-oxododecanoyl)-l-homoserine lactone **3** (OdDHL) (**3**). (**A**) Compound **13** shows OdDHL-like interactions and orientation. Hydrogen bonds are in pink. OdDHL is in yellow. (**C**) Compound **23** shows a flipped orientation relative to the lactone ring of OdDHL. (**B**,**D**) describe the interactions of **13** and **23** with LasR. Hydrogen bonds are green. Halogen interactions are blue. Hydrophobic and electrostatic interactions are red.

The ligand-LasR interactions of Compounds **9**–**42** were analysed based on the highest scoring docked pose of the largest cluster. The predicted binding poses of Compounds **10**, **11**, **13**, **15**, **22**, **26**, and **31**–**42** were observed to be very similar to OdDHL, with the lactam carbonyl group of most compounds forming a hydrogen bond with Trp60 from the same position as the lactone ring of the natural ligand (Figure 3A,B). Interestingly, the remaining compounds were predicted to bind in a different pose, with the molecule flipped (Figure 3C). This alternate pose still allowed the carbonyl group of the lactam ring to form a hydrogen bond with Trp60, as was predicted for Compounds **9**, **12**, **14** and **25**; however, it also allowed the lactam CO to form a hydrogen bond with Arg61, which was observed for the remaining compounds.

It was further observed that most of the compounds with high QSI were predicted to bind in a pose different from OdDHL. One explanation for this may be similar to that proposed by Bottomley et al., in that this pose severely limits the interaction with the hydrophobic pocket of the receptor and thus inhibits the formation of a stable protein conformation [26]. Previous studies by Gerdt et al. and Bottomley et al. have identified that hydrogen bonds to residues Ser129 and Thr115 are important for agonistic activity of ligands [26,27]. Notably, none of the QS inhibitors in this study were observed to form these hydrogen bonds, which is consistent with the antagonistic activity seen for these compounds. The predicted binding poses for Compounds **12** and **14** both shared the lactam ring in the same position, resulting in the main difference between the two being the interactions of the hydroxyl group of each compound. In Compound **12**, the *ortho* hydroxyl group was predicted to form a hydrogen bond to Tyr56, whilst in the less active Compound **14,** the *para* hydroxyl group formed a hydrogen bond with Tyr93. Hydrogen bonding to Tyr56 has been identified to be a key interaction in determining the agonistic or antagonistic behaviour of a compound, which may explain the much greater QSI activity observed for **12** [27].

The potent QS inhibitors **19**, **23** and **24** form the same hydrogen bond with Arg61 and have a flipped orientation relative to the lactone head, but they form different interactions between the phenyl and the LasR binding pocket. The 4-aminophenyl group in Compound **19** forms an electrostatic π-anion with Asp73. This suggested that NH_2_ is not required for activity since Compounds **23** and **24** lack the amino group and yet have potent QS inhibition. Although there are no significant differences in QS activity between **23** and **24**, the phenyl groups differ in the type of interactions they produce. Compound **23** makes hydrophobic interactions as shown in Figure 3C,D, while Compound **24** only forms an electrostatic π-anion interaction with Asp73. For the majority of docked compounds, the halogen at the 3-position of the lactam ring was not predicted to make any interactions with the LasR pocket except halogen interaction with Leu110 for Compound **13** and the least active compounds including **15**, **33**, **35**–**38** and **40**–**41**. In contrast, the halogen at the 4-position was predicted to form hydrophobic interactions with the pocket for every docked compound including the most active ones. To further investigate the importance of these observations, a new series of compounds similar to those detailed in this study should be synthesized that lack a halogen at the 3-position of the lactam ring and have various hydrophobic alkyl or aromatic groups at the 4-position. Detailed docking results are provided in the Supplementary Information.

## 3. Conclusions

A small library of 34 lactam compounds was synthesized and evaluated for QS inhibition against *P. aeruginosa*. Compounds **9**–**42** were prepared in moderate to high yields via the reductive amination of mucochloric and mucobromic acid with a wide range of amines, including aliphatic, aromatic and heteroaromatic amines. In biological testing, several compounds possessed promising activities, with **12**, **13**, **23** and **24** being the most active and showing comparable or even superior activity to the positive controls TP-5 and Fu-30 (**5**). The tested compounds showed low bacterial growth inhibition in contrast to Fu-30. Amides were also introduced to Compounds **16** and **20** to give the C-linked amides **32**–**38** and the *N*-linked amides **39**–**42**, respectively. Generally, the amides **32**–**42** were less active compared to lactams **9**–**31**, although the C-linked amides **32**–**38** were more potent than the *N*-linked amides **39**–**42**. Several compounds showed high efficacy in pyocyanin inhibition, and the results are consistent with their potent QS activity. Docking of the synthesized compounds to the LasR receptor protein predicted favourable intermolecular interactions similar to OdDHL, including a hydrogen bond with the conserved polar Arg61 and Trp60 residues. The most active compounds were docked in a different orientation compared to OdDHL and did not form hydrogen bonds implicated in the stabilization of LasR by agonists. Overall, the results obtained from this study suggest that lactams derived from mucochloric and mucobromic acid could serve as new lead compounds for the development of potent QSI compounds that are unlikely to exert selective pressure on bacteria.

## 4. Experimental

### 4.1. Chemistry

Commercially-available reagents were purchased from Aldrich, Acros Organics, Alfa Aesar. The synthetic procedures have been reported for all compounds as general methods and appropriate references have been given for known compounds. Melting points were measured using a Mel-Temp melting point apparatus and were used uncorrected. High-resolution [+ESI] mass spectra were recorded by the Bioanalytical Mass Spectrometry Facility, UNSW, on an Orbitrap LTQ XL ion trap mass spectrometer using a nanospray (nano-electrospray) ionization source. ^1^H and ^13^C NMR spectra were determined in the designated solvent on a Bruker DPX 300 spectrometer or a Bruker Avance 400 spectrometer unless otherwise stated. Chemical shifts (δ) are quoted in parts per million (ppm), to the nearest 0.01 ppm and internally referenced relative to the solvent nuclei. ^1^H NMR spectral data are reported with their chemical shift in parts per million (ppm). The multiplicity in ^1^H NMR is abbreviated as follows: brs, broad; s, singlet; d, doublet; t, triplet; q, quartet; quint, quintet; sext, sextet; m, multiplet; or as a combination (e.g., dd, dt, etc.). The coupling constant (*J*) in hertz, integration and proton count were determined.

### 4.2. General Procedure

Procedure A: Reductive amination

Mucohalic acid (1 eq) was added to a solution of 5:3 *v*/*v* dichloromethane/glacial acetic acid. Then, an amine (1 eq) was added, and the mixture was stirred for 10 min. To that mixture, sodium triacetoxyborohydride (3 eq) solution in 5:3 *v*/*v* dichloromethane and glacial acetic acid was added. The mixture was left to stir at room temperature for 24 h unless otherwise stated.

Procedure B: Amide coupling 1

Acid **16** (1 eq) was treated with thionyl chloride (3 mL) and refluxed for 3 h at 70 °C. The mixture was then cooled and left to stir overnight at room temperature. Thionyl chloride was removed under high vacuum, and the resultant acid chloride was used without further purification. The acid chloride was dissolved in 10 mL of dry tetrahydrofuran, and triethylamine (1 eq) and amine (1 eq) were then added. The reaction mixture was left to stir at room temperature for 24 h.

Procedure C: Amide coupling 2

The amine was dissolved in 10 mL of dry tetrahydrofuran, and then, triethylamine (1 eq) and acid chloride (1.0 eq) were added. The reaction mixture was left to stir at room temperature for 24 h.

(1) 1-butyl-3,4-dichloro-1,5-dihydro-2*H*-pyrrol-2-one (**9**)


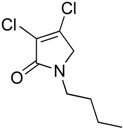


The title compound was prepared according to Procedure A from mucochloric acid (400 mg, 2.3 mmol), butyl amine (170 mg, 270 mL, 2.3 mmol) and sodium triacetoxyborohydride (1.46 g, 6.9 mmol). The reaction mixture was left to react for 24 h. The crude mixture was extracted with dichloromethane (30 mL) and washed with water (15 mL) followed by brine (15 mL), dried over sodium sulphate, and the solvent was evaporated. A residue of oil was obtained, which was purified by flash column chromatography using a gradient eluent of 25–50% ethyl acetate in hexane. A yellow oil was obtained (390 mg; 79%). ^1^H NMR (CDCl_3_, 400MHz): *δ* 0.95 (t, *J* = 7.3, 14.6 Hz, 3H, CH_3_), *δ* 1.36 (sext, *J* = 7.2, 15.0 Hz, 2H, CH_2_), 1.58 (quint, *J* = 7.2, 15.0 Hz, 2H, CH_2_), *δ* 3.50 (t, *J* = 7.4, 15.0, 2H, CH_2_), *δ* 4.04 (s, 2H, CH_2_). ^13^C NMR (CDCl_3_, 101): 13.7 (CH_3_), 19.9 (CH_2_), 30.4 (CH_2_), 42.8 (CH_2_), 53.4 (C5-CH_2_), 125.9 (C), 139.0 (C), 164.2 (C); IR (ATR): υ_max_ 2957.6, 2870.7, 1700.9, 1397.7, 1341.6, 1269.6, 1188.5, 1130.0, 843.9; UV (MeOH) λ_max_ 230.0 nm (ε 7488 cm^−1^M^−1^)HRMS (ESI) *m*/*z* calcd. for C_8_H_11_Cl_2_N_1_O_1_Na_1_ 230.0110 [M + Na]^+^, found 230.0108.

(2) 3,4-dichloro-1-hexyl-1,5-dihydro-2*H*-pyrrol-2-one (**10**)


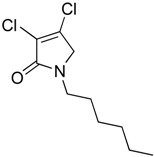


The title compound was prepared according to Procedure A from mucochloric acid (400 mg, 2.3 mmol), hexyl amine (300 mg, 270 mL, 2.3 mmol) and sodium triacetoxyborohydride (1.46 g, 6.9 mmol). The reaction mixture was left to react for 24 h. The crude mixture was extracted with dichloromethane (30 mL) and washed with water (15 mL) followed by brine (15 mL), dried over sodium sulphate, and the solvent was evaporated and a residue oil obtained, which was purified by flash column chromatography using a gradient eluent of 25–50% ethyl acetate in hexane. A yellow oil was obtained (220 mg; 39%); ^1^H NMR (CDCl_3_, 400 MHz): *δ* 0.90 (t, *J* = 6.9, 13.5 Hz, 3H, CH_3_), *δ* 1.24 (m, 6H, CH_2_), *δ* 1.60 (m, 2H, CH_2_), *δ* 3.49 (t, *J* = 7.4, 14.6 Hz, CH_2_), *δ* 4.04 (s, 2H, C5-CH_2_). ^13^C NMR (CDCl_3_, 101): 14.0 (CH_3_), 22.5 (CH_2_), 26.3 (CH_2_), 28.3 (CH_2_), 31.4 (CH_2_), 43.1 (CH_2_), 53.4 (C5-CH_2_), 125.7 (C), 139.2 (C), 164.2 (C=O); IR (ATR): υ_max_ 2927.2, 2857.2, 1702.3, 1438.2, 1397.4 1367.5, 1131.0, 1171.0, 953.7; UV (MeOH) λ_max_ 235.0 nm (ε 4248 cm^−1^M^−1^); HRMS (ESI) *m*/*z* calcd. for C_10_H_16_Cl_2_N_1_O_1_ 236.0603 [M + H]^+^, found 236.0601.

(3) 1-benzyl-3,4-dichloro-1,5-dihydro-2*H*-pyrrol-2-one (**11**)


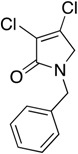


The title compound was made according to Procedure A following the reported method [14]. m.p. 102.1 °C; ^1^H NMR (CDCl_3_, 400 MHz): *δ* 3.92 (s, 2H, CH_2_), 4.67 (s, 2H, CH_2_), 7.26–7.39 (m, 5H, ArH); ^13^C NMR (CDCl_3_, 101): δ 47.0 (CH_2_), 52.8 (CH_2_), 125.5 (C), 128.2 (ArCH), 129.0 (ArCH), 135.8, 139.9 (C), 164.28 (C=O); IR (ATR): υ_max_ 2917.9, 2850.9, 1693.6, 1437.5, 1266.9, 966.1, 846.2, 739.1.

(4) 3,4-dichloro-1-(2-hydroxyphenyl)-1,5-dihydro-2*H*-pyrrol-2-one (**12**)


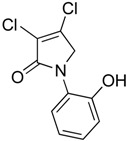


The title compound was prepared according to Procedure A from mucochloric acid (600 mg, 3.55 mmol), *ortho*-aminophenol (388 mg, 3.55 mmol) and sodium triacetoxyborohydride (2.26 g, 10.65 mmol). The reaction mixture was left to react for 48 h. The precipitated solid was filtered by vacuum filtration, and a white solid was obtained (280 mg; 32%). m.p. 171.6 °C; ^1^H NMR (DMSO-*d*_6_, 300 MHz): *δ* 4.61 (s, 2H, CH_2_), *δ* 6.85–6.97 (m, 2H, ArH), *δ* 7.17–7.28 (m, 2H, ArH), 9.86 (s, OH); ^13^C NMR (DMSO, 75.5 MHz): 55.4 (CH_2_), 117.2 (ArCH), 119.6 (ArCH), 124.3 (C), 124.4 (ArCH), 129.3 (ArCH), 129.6 (ArCH), 142.0 (ArC), 153.5 (C), 163.1 (C=O); IR (ATR): υ_max_ 3105.4, 1666.0, 1591.4, 1509.6, 1445.4, 1275.3, 1198.2, 930.5, 839.1; UV (MeOH): λ_max_ 280.0 nm (ε 2928 cm^−1^M^−1^); HRMS (ESI) *m*/*z* calcd. for C_10_H_7_Cl_2_N_1_O_2_Na_1_265.9746 [M + Na]^+^, found 265.9744.

(5) 3,4-dichloro-1-(3-hydroxyphenyl)-1,5-dihydro-2*H*-pyrrol-2-one (**13**)


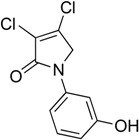


The title compound was prepared according to Procedure A following the reported method [14] from mucochloric acid (600 mg, 3.55 mmol), *meta*-aminophenol (388 mg, 3.55 mmol) and sodium triacetoxyborohydride (2.26 g, 10.65 mmol). The reaction mixture was left to react for 24 h. As the reaction progresses, a yellow precipitated solid was evident. The solid was filtered by vacuum filtration and a yellow solid was obtained (578 mg; 66%); m.p. 163.6 °C; ^1^H NMR (DMSO-*d*_6_, 400 MHz): *δ* 4.80 (s, 2H, CH_2_), *δ* 6.59 (d, *J* = 8.0 Hz, 1H, ArH), 7.04 (d, *J* = 9.3 Hz, 1H, ArH), 7.18 (t, *J* = 8.0 Hz, 1H, ArH), 7.28 (s, 1H, ArH), 9.71 (brs, OH); ^13^C NMR (DMSO, 100 MHz): 54.0 (CH_2_), 106.6 (ArCH), 109.8 (ArCH), 112.3 (ArCH), 124.3 (C), 130.2 (ArCH), 139.8 (C), 141.9 (C), 158.4 (C), 162.4 (C=O); UV (MeOH): λ_max_ 280.0 nm (ε 3854 cm^−1^M^−1^); HRMS (ESI) *m*/*z* calcd. for C_10_H_7_Cl_2_N_1_O_2_Na_1_ 265.9746 [M + Na]^+^, found 265.9745.

(6) 3,4-dichloro-1-(4-hydroxyphenyl)-1,5-dihydro-2*H*-pyrrol-2-one (**14**)


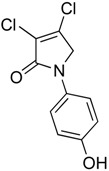


The title compound was prepared according to Procedure A from mucochloric acid (600 mg, 3.55 mmol), 4-aminophenol (388 mg, 3.55 mmol) and sodium triacetoxyborohydride (2.26 g, 10.65 mmol). The reaction mixture was left to react for 24 h. As the reaction progressed, a yellow precipitated solid was evident. The solid was filtered by vacuum filtration, and a yellow solid was obtained (500 mg; 58%); m.p. 126.0 °C; ^1^H NMR (DMSO-*d*_6_, 400 MHz): *δ* 4.77 (s, 2H, CH_2_), *δ* 6.81 (d, *J* = 8.9 Hz, 2H, ArH), *δ* 7.44 (d, *J* = 8.9 Hz, 2H, ArH), 9.8 (brs, 1H, OH); ^13^C NMR (DMSO, 100 MHz): 54.5 (CH_2_), 115.8 (ArCH), 122.2 (ArCH), 124.3 (C), 130.3 (ArC), 141.2 (ArC), 155.4 (C), 162.1 (C=O), IR (ATR): υ_max_ 3277.8, 1682.9, 1630.2, 1510.6, 1270.8, 1219.3, 1046.9, 934.0, 830.7; UV (MeOH): λ_max_ 280.0 nm (ε 1090.2 cm^−1^M^−1^); HRMS (ESI) *m*/*z* calcd. for C_10_H_7_Cl_2_N_1_O_2_Na_1_ 265.9746 [M + Na]^+^, found 265.9745.

(7) *N*-(3-carboxyphenyl)-3,4-dibromo-1,5-dihydro-2*H*-pyrrol-2-one (DHP phenyl acid-2) (**15**)


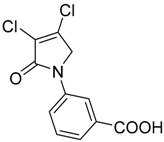


The title compound was synthesized according to Procedure A from mucochloric acid (1 g, 5.91 mmol), 3-aminobenzoic acid (0.81 g, 5.91 mmol) and sodium triacetoxyborohydride (3.76 g, 17.75 mmol) in 5:3 *v*/*v* dichloromethane/glacial acetic acid (12 mL). The reaction mixture was left to stir at room temperature for 18 h, during which time, a yellow precipitate was evident. The mixture was filtered under vacuum, and the filtered solid was purified by flash chromatography. The solid was then recrystallized in methanol after chromatography to yield the pure title product as a white solid (0.54g; 34%); m.p. 214–216 °C; ^1^H NMR (300 MHz, DMSO-*d*_6_) δ 4.93 (s, 2H, CH_2_), δ 7.55 (t, *J* = 7.98 Hz, 1H, ArH), δ 7.74 (tt, *J* = 7.98 and 1.48 Hz, 1H, ArH), δ 7.89–7.93 (m, 1H, ArH), δ 8.34 (t, *J* = 1.8 Hz, 1H, ArH), δ 13.13 (brs, 1H, COOH); ^13^C NMR (75.5 MHz, DMSO-*d*_6_) δ 54.0 (CH_2_), 120.0 (ArCH), 123.4 (ArCN), 125.8 (ArC), 129.9 (ArCH), 132.1 (ArCH), 138.9 (ArCH), 142.4 (2 × CCl), 162.7 (C=O), 167.3 (C=O); IR (ATR): υ_max_ 2969, 2824, 2654, 2539, 1699, 1586, 1491, 1433, 1382, 1313, 1272, 1158, 1051, 938, 899, 818, 757, 739, 677 cm^−1^; UV (THF): λ_max_ 236 nm (ε 20671 cm^−1^M^−1^), 288 (32,346); HRMS (ESI) *m*/*z* calcd. for C_11_H_7_Cl_2_NO_3_Na 293.9695 [M + Na]^+^, found 293.9698.

(8) *N*-(4-carboxyphenyl)-3,4-dichloro-1,5-dihydro-2*H*-pyrrol-2-one (**16**)


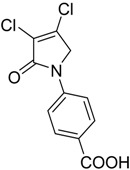


The title compound was synthesized according to Procedure A, by first dissolving mucochloric acid (1 g, 5.91 mmol) in 5:3 *v*/*v* dichloromethane/glacial acetic acid (12 mL). To this mixture, a solution of *p*-aminobenzoic acid (0.81 g, 5.91 mmol) in dichloromethane (8 mL) was added followed by sodium triacetoxyborohydride (3.76 g, 17.75 mmol). The reaction mixture was stirred at room temperature for 18 h, during which time a yellow precipitate was evident. The mixture was filtered under vacuum and washed with dichloromethane and distilled water to yield a yellow solid (0.6 g; 37%). m.p. 229 °C; ^1^H NMR (300 MHz, DMSO-*d*_6_) δ 4.91 (s, 2H, CH_2_), δ 7.84–7.88 (m, 2H, ArH), δ 7.96–8.01 (m, 2H, ArH), δ 12.8 (brs, 1H, COOH); ^13^C NMR (75.5 MHz, DMSO-*d*_6_) δ 53.9 (CH_2_), 118.3 (2 × ArCH), 124.2 (ArCN), 126.7 (ArC), 130.9 (2 × ArCH), 142.4 (CCl), 142.8 (CCl), 162.8 (C=O), 167.1 (C=O); IR (ATR): υ_max_ 2939, 2814, 2659, 2537, 1679, 1604, 1516, 1425, 1375, 1279, 1042, 934, 860, 769, 741, 718 cm^−1^; UV (THF): λ_max_ 236 nm (ε 5338 cm^−1^M^−1^), 289 (2736); HRMS (ESI) *m*/*z* calcd. for C_11_H_7_Cl_2_NO_3_Na 293.9695 [M + Na]^+^, found 293.9696.

(9) *N*-(3-tert-butylphenylcarbamate)-3,4-dichloro-1,5-dihydro-2*H*-pyrrol-2-one (**17**)


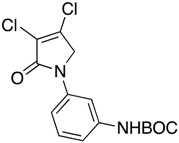


The title compound was prepared from mucochloric acid (1.0 g, 5.91 mmol), *N*-Boc-m-phenylenediamine (1.2 g, 5.91 mmol) and sodium triacetoxyborohydride (3.7 g, 17.75 mmol) in 5:3 *v*/*v* dichloromethane/glacial acetic acid (12 mL). The mixture was stirred at room temperature for 3.5 h. The reaction mixture was washed with water and brine and then extracted into ethyl acetate. The organic layer was dried over sodium sulphate and evaporated in vacuo. The solid was recrystallized in DCM and hexane to yield the title compound as a yellow solid (1.36 g, 67.17%). m.p. 131–132 °C; ^1^H NMR (300 MHz, DMSO-*d*_6_) δ 1.48 (s, 9H, 3 × CH_3_), δ 4.80 (s, 2H, CH_2_), δ 7.29 (m, 3H, ArH), 7.97 (s, 1H, ArH), δ 9.45 (s, 1H, NH); ^13^C NMR (100 MHz, DMSO-*d*_6_) δ 28.6 (3 × CH_3_), δ 54.1 (CH_2_), δ 79.6.0 (C-O), 113.2 (ArC), 115.1 (ArC), 124.2 ©, 129.6 (ArCH), 139.0 (ArCH), 140.7 (ArC), 153.2 (C=O), 162.4 (C=O); IR (ATR): υ_max_ 3305, 2977, 1695, 1604, 1498, 1421, 1388, 1285, 1227, 1151, 945, 876, 683, cm^−1^; HRMS (ESI) *m*/*z* calcd. for C_15_H_16_Cl_2_N_2_O_3_Na 365.0430 [M + Na]^+^, found 381.0428.

(10) *N*-(4′-tert-butylphenylcarbamate)-3,4-dichloro-1,5-dihydro-2*H*-pyrrol-2-one (**18**)


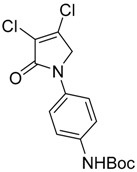


The title compound was prepared according to Procedure A from mucochloric acid (0.9 g, 5.36 mmol), *N*-Boc-*p*-phenylenediamine (1.1 g, 5.36 mmol) and sodium triacetoxyborohydride (3.38 g, 15.98 mmol) in 5:3 *v*/*v* dichloromethane/glacial acetic acid (12 mL). The mixture was stirred at room temperature for 3 h. The reaction mixture was washed with water and brine and then extracted into ethyl acetate. The organic layer was dried over sodium sulphate and evaporated in vacuo to yield the title compound as a dark red solid (1.16 g, 64%). m.p. 130–131 °C; ^1^H NMR (400 MHz, DMSO-*d*_6_) δ 1.48 (s, 9H, 3 × CH_3_), δ 4.8 (s, 2H, CH_2_), δ 7.46 (dd, *J* = 41.9 and 9 Hz, 4H, ArH), δ 9.38 (s, 1H, NH); ^13^C NMR (100 MHz, DMSO-*d*_6_) δ 28.5 (3 × CH_3_), δ 54.1 (CH_2_), δ 82.0 (C-O), δ 118.9 (2 × ArCH), 120.3 (ArC), 124.2 (ArC), 133.1 (2 × ArCH), 136.9 (CCl), 141.5 (CCl), 153.2 (C=O), 162.2 (C=O); IR (ATR): υ_max_ 3345, 2923, 2321, 1698, 1589, 1519, 1385, 1312, 1228, 1151, 1025, 932, 831, 758 cm^−1^; UV (ACN): λ_max_ 245 nm (ε 13,990 cm^−1^M^−1^); HRMS (ESI) *m*/*z* calcd. for C_15_H_16_Cl_2_N_2_O_3_Na 365.0430 [M + Na]^+^, found 381.0426.

(11) *N*-(3-aminophenyl)-3,4-dichloro-1,5-dihydro-2*H*-pyrrol-2-one (**19**)


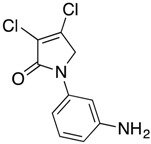


The title compound was prepared according to Procedure A using Compound **17**. The Boc group was cleaved by treating **17** (1.36 g) with trifluoroacetic acid (6 mL) at room temperature for 1 h followed by evaporation of TFA under high vacuum. The residue was neutralized and washed with saturated solution of sodium bicarbonate, and the solid obtained was filtered under vacuum to yield a red solid (0.75 g, 78%). m.p. 109 °C; ^1^H NMR (300 MHz, DMSO-*d*_6_) δ 4.75 (s, 2H, CH_2_), 5.21 (brs, 2H, NH_2_), δ 6.43 (dd, *J* = 6.42, 7.7 Hz, 1H, ArH), 6.78 (dd, *J* = 6.4, 7.7 Hz, 1H, ArH), 7.04–7.12 (m, 2H, ArH); ^13^C NMR (75.5 MHz, DMSO-*d*_6_) δ 54.0 (CH_2_), δ 105.4 (ArCH), 107.3 (ArCH), 111.4 (ArCH), 124.3 (C), 129.8 (ArCH), 139.4 (ArC), 141.6 (ArC), 149.0 (C), 162.2 (C=O); IR (ATR): υ_max_ 3340, 1687, 1466, 1389, 1268, 949, 758, HRMS (ESI) *m*/*z* calcd. for C_10_H_8_Cl_2_N_2_O^1^ 243.0086 [M + 1]^+^, found 243.0084.

(12) *N*-(4-aminophenyl)-3,4-dichloro-1,5-dihydro-2*H*-pyrrol-2-one (**20**)


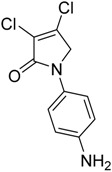


The title compound was prepared according to Procedure A from the Boc group, which was cleaved by treating intermediate **18** (0.53 g) with trifluoroacetic acid (5 mL) at room temperature for 1 h followed by evaporation of TFA under high vacuum. The residue was neutralized and washed with saturated solution of sodium bicarbonate, and the solid obtained was filtered under vacuum to yield a red solid (0.25 g, 64%). m.p. 106 °C; ^1^H NMR (300 MHz, DMSO-*d*_6_) δ 4.7 (s, 2H, CH_2_), δ 5.13 (brs, 1H, NH_2_), δ 6.55–6.6 (m, 2H, ArH), δ 7.24–7.29 (m, 2H, ArH); ^13^C NMR (75.5 MHz, DMSO-*d*_6_) δ 54.6 (CH_2_), δ 114.2 (2 × ArCH), 121.5 (ArC), 122.3 (2 × ArCH), 127.5 (ArC), 140.6 (CCl), 146.9 (CCl), 163.0 (C=O); IR (ATR): υ_max_ 3297, 3205, 2921, 1697, 1636, 1515, 1400, 1389, 1300, 1175, 1043, 928, 812 cm^−1^; UV (ACN): λ_max_ 247 nm (ε 10,817 cm^−1^M^−1^), 307 (5783); HRMS (ESI) *m*/*z* calcd. for C_10_H_8_Cl_2_N_2_ONa 264.9906 [M + Na]^+^, found 264.9909.

(13) 4-(3,4-dichloro-2-oxo-2,5-dihydro-1*H*-pyrrol-1-yl)butanoic acid (**21**)


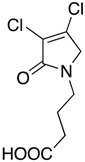


The title compound was synthesized according to Procedure A, by first dissolving mucochloric acid (1.0 g, 5.9 mmol) in 5:3 *v*/*v* dichloromethane/glacial acetic acid (12 mL). To this mixture, a solution of aminobutanoic acid (0.61 g, 5.9 mmol) in dichloromethane (8 mL) was added followed by sodium triacetoxyborohydride (3.76 g, 17.75 mmol) to yield a white solid (0.41 g, 30%). m.p. 106 °C; ^1^H NMR (300 MHz, DMSO-*d*_6_) δ 1.91 (m, CH_2_), δ 2.42 (m, CH_2_), δ 3.56 (t, *J* = 7.0 Hz), δ 4.07 (s, C5-CH_2_); ^13^C NMR (75.5 MHz, DMSO-*d*_6_) δ 23.42 (CH_2_), 30.82 (CH_2_), 42.40 (CH_2_), 53.56 (C5-CH_2_), 125.7 (C), 139.7 (C), 164.7 (C=O), 176.9 (C=O); IR (ATR): υ_max_ 3936, 2641, 1735, 1339, 1289, 1195, 859, 745 cm^−1^; HRMS (ESI) *m*/*z* calcd. for C_8_H_9_Cl_2_N_1_O_3_Na_1_259.9852 [M + Na]^+^, found 259.9850.

(14) 1-allyl-3,4-dichloro-1,5-dihydro-2*H*-pyrrol-2-one (**22**)


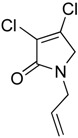


The title compound was prepared according to Procedure A from mucochloric acid (600 mg, 3.55 mmol), allylamine (202.7 mg, 3.55 mmol) and sodium triacetoxyborohydride (2.26 g, 10.65 mmol). The reaction mixture was left to react for 3 days. The crude mixture was extracted with dichloromethane (30 mL) and washed with water (15 mL) followed by brine (15 mL), dried over sodium sulphate, and the solvent was evaporated and a residue oil obtained, which was purified by flash column chromatography using a gradient eluent of 5% to 25% ethyl acetate and hexane. A yellow oil was obtained (244 mg, 36%); ^1^H NMR (CDCl_3_, 300 MHz): 4.01 (s, 2H, C5-CH_2_), 4.11 (d, *J* = 6.1 Hz, 2H, *N*-CH_2_), 5.20–5.80 (m, 2H, HC=CH_2_), 5.74–5.80 (m, 1H, HC=CH_2_); ^13^C NMR (CDCl_3_, 75.5MHz): 45.5 (CH_2_), 52.9 (C5-CH_2_), 119.0 (HC=CH_2_), 125.6 (CCl), 132.0 (HC=CH_2_), 139.8 (C) 164.0 (C); IR (ATR): υ_max_ 3290.1, 1685.5, 1621.0, 1402.1, 1270.5, 1157.0, 1100.6, 929.8, 841.8; UV (MeOH): λ_max_ 280.0 nm (ε 4928 cm^−1^M^−1^); HRMS (ESI) *m*/*z* calcd. for C_7_H_7_Cl_2_N_1_O_1_Na_1_ 213.9797 [M + Na]^+^, found 213.9794.

(15) 3,4-dichloro-1-phenyl-1,5-dihydro-2*H*-pyrrol-2-one (**23**)


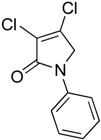


The title compound was prepared according to Procedure A from mucochloric acid (1g, 5.92 mmol), aniline (551.2 mg, 5.92 mmol) and sodium triacetoxyborohydride (3.76 g, 17.76 mmol). The reaction mixture was left to react for 48 h. The reaction mixture was extracted with dichloromethane (30 mL) and washed with water (15 mL) followed by brine (15 mL), dried over sodium sulphate, and the solvent was evaporated. The obtained semi solid was triturated with methanol, and the precipitated solid was collected by vacuum filtration and dried over silica in the desiccator. A white small needle solid was obtained (680 mg, 99%). m.p. 201.2 °C; ^1^H NMR (DMSO-*d*_6_, 300 MHz): *δ* 4.86 (s, 2H, CH_2_), 7.20 (t, *J* = 9.0 Hz, 1H, ArH), 7.43 (t, *J* = 9.00 Hz, 2H, ArH), 7.73 (d, *J* = 9.0 Hz, 2H, ArH); ^13^C NMR (DMSO, 75.5MHz): 54.0 (CH_2_), 119.5 (ArCH), 124.3 (C), 125.1 (ArC)), 129.6 (ArCH), 139.0 (C), 142.0 (C), 162.5 (C=O); IR (ATR): υ_max_ 3060.4, 2918.3, 1688.4, 1628.9, 1500.0, 1437.1, 1298.7, 1153.3, 1049.2, 927.4, 755.6; UV (MeOH): λ_max_ 280.0 nm (ε 3854.4 cm^−1^M^−1^); HRMS (ESI) *m*/*z* calcd. for C_10_H_7_Cl_2_N_1_O_1_Na_1_ 249.9797 [M + Na]^+^, found 249.9794. 

(16) 3,4-dibromo-1-phenyl-1,5-dihydro-2*H*-pyrrol-2-one (**24**)


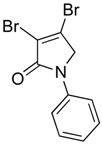


The title compound was prepared according to Procedure A from mucobromic acid (1.20 g, 4.65 mmol), aniline (433.3 mg, 4.65 mmol) and sodium triacetoxyborohydride (3.60 g, 13.95 mmol). The reaction mixture was left to stir for 48 h. The precipitated solid was filtered by vacuum filtration, and a white solid was obtained (440 mg; 30%); m.p. 160.7 °C; ^1^H NMR (DMSO-*d*_6_, 300 MHz): *δ* 4.88 (s, 2H, CH_2_), 7.18 (t, *J* = 7.5 Hz, 1H, ArH), 7.43 (t, *J* = 7.5 Hz, 2H, ArH), 7.72 (d, *J* = 8.7 Hz, 1H, ArH); ^13^C NMR (DMSO, 75.5 MHz): 57.3 (CH_2_), 119.4 (ArCH), 119.8 (ArC), 125.0 (C), 129.5 (ArCH), 136.4 (C), 138.8 (C), 163.3 (C); IR (ATR): υ_max_ 3059.5, 1692.5, 1589.5, 1421.5, 1374.4, 1145.1, 1035.1, 885.4, 753.7; UV (MeOH): λ_max_ 280.0 nm (ε 7829 cm^−1^M^−1^); HRMS (ESI) *m*/*z* calcd. for C_10_H_7_Br_2_N_1_O_1_Na_1_ 337.8787 [M + Na]^+^, found 337.8789.

(17) 3,4-dibromo-1-(3-hydroxyphenyl)-1,5-dihydro-2*H*-pyrrol-2-one (**25**)


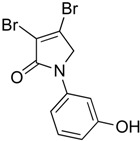


The title compound was prepared according to Procedure A from mucobromic acid (1.20 g, 4.65 mmol), *meta*-aminophenol (507.8 mg, 4.65 mmol) and sodium triacetoxyborohydride (3.60 g, 13.95 mmol). The reaction mixture was left to stir for 48 h. The precipitated solid was filtered by vacuum filtration, and a light brown solid was obtained (780 mg, 50%); m.p. 164.0 °C; ^1^H NMR (DMSO-*d*_6_, 300 MHz): *δ* 4.80 (s, 2H, CH_2_), 6.57 (m, 2H, ArH), 7.05–7.27 (m, 2H, ArH), *δ* 9.59 (s, 1H, OH); ^13^C NMR (DMSO, 75.5 MHz): *δ* 57.4 (CH_2_), 106.6 (ArCH), 109.8 (ArCH), 112.2 (ArCH), 119.8 (C), 130.2 (ArCH), 136.4 (C), 139.9 (C), 158.3 (C), 163.2 (C=O); IR (ATR): υ_max_ 3257.5, 1664.0, 1597.9, 1456.1, 1392.4, 1255.0, 1213.9, 1039.0, 929.8, 867.1; UV (MeOH): λ_max_ 290.0 nm (ε 5893 cm^−1^M^−1^); HRMS (ESI) *m*/*z* calcd. for C_10_H_7_Br_2_N_1_O_2_Na_1_ 353.8736 [M + Na]^+^, found 353.8733.

(18) *N*-(3-carboxyphenyl)-3,4-dichloro-1,5-dihydro-2*H*-pyrrol-2-one (**26**)


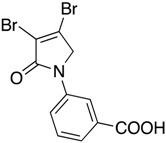


The title compound was prepared from mucobromic acid (2.0 g, 7.75 mmol), *meta*-aminobenzoic acid (1.06 g, 7.75 mmol) and sodium triacetoxyborohydride (4.93 g, 23.26 mmol). The reaction mixture was left to stir for 24 h. The precipitated solid was filtered by vacuum filtration to afford the product as pale yellow solid (0.36 g, 26%). m.p. 186 °C; ^1^H NMR (300 MHz, DMSO-*d*_6_) δ 4.93 (s, 2H, CH_2_), δ 7.55 (t, *J* = 8.0 Hz, 1H, ArH), δ 7.73 (tt, *J* = 8.0 and 1.47 Hz, 1H, ArH), δ 7.89–7.93 (m, 1H, ArH), δ 8.33 (t, *J* = 1.8 Hz, 1H, ArH), δ 13.09 (brs, 1H, COOH); ^13^C NMR (75.5 MHz, DMSO-*d*_6_) δ 57.3(CH_2_), 119.9 (ArCH), 123.3 (ArCN), 125.7 (ArC), 129.8 (ArCH), 136.9 (ArCH), 139.0 (ArCH), 146.0 (2 × CBr), 163.5 (C=O), 167.4 (C=O); IR (ATR): υ_max_ 2821, 2551, 2321, 1698, 1584, 1490, 1425, 1380, 1312, 1289, 1227, 1151, 1032, 939, 901, 840, 757, 673 cm^−1^; UV (THF): λ_max_ 239 nm (ε 15,683cm^−1^M^−1^), 291 (6639); HRMS (ESI) *m*/*z* calcd. for C_11_H_7_Br_2_NO_3_Na 381.8685 [M + Na]^+^, found 381.8685.

(19) *N*-(4-carboxyphenyl)-3,4-dichloro-1,5-dihydro-2*H*-pyrrol-2-one (**27**)


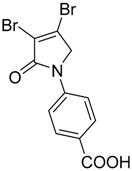


The title compound was synthesized from mucochloric acid (3 g, 11.63 mmol), *p*-aminobenzoic acid (1.59 g, 11.63 mmol) and sodium triacetoxyborohydride (7.39 g, 34.9 mmol) in 5:3 *v*/*v* dichloromethane/glacial acetic acid (30 mL). The reaction mixture was stirred and heated at 30 °C for 3 h during which time a precipitate was evident. The mixture was filtered under vacuum, and the solid was recrystallized in 1:9 acetone/methanol to get the desired product as a white solid (0.296 g, 7%). m.p. 221 °C; ^1^H NMR (300 MHz, DMSO-*d*_6_) δ 4.9 (s, 2H, CH_2_), δ 7.83 (dd, *J* = 37.8 and 8.7 Hz, 4H, ArH), δ 12.8 (brs, 1H, COOH); ^13^C NMR (75.5 MHz, DMSO-*d*_6_) δ 57.2 (CH_2_), 118.2 (2 × ArCH), 120.0 (ArCN), 126.6 (ArC), 130.9 (2 × ArCH), 137.4 (CBr), 142.5 (CBr), 163.7 (C=O), 167.1 (C=O); IR (ATR): υ_max_2811, 2659, 2535, 2112, 1679, 1601, 1516, 1423, 1371, 1275, 1188, 1145, 1017, 929, 889, 757, 704 cm^−1^; UV (THF): λ_max_ 245 nm (ε 14,965cm^−1^M^−1^), 288 (15,575); HRMS (ESI) *m*/*z* calcd. for C_11_H_7_Br_2_NO_3_Na 381.8685 [M + Na]^+^, found 381.8684.

(20) *N*-(4′-tert-butylphenylcarbamate)-3,4-dibromo-1,5-dihydro-2*H*-pyrrol-2-one (**28**)


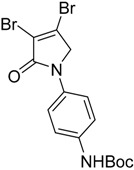


The title compound was synthesized by reacting mucobromic acid (1 g, 3.87 mmol), *N*-Boc-p-phenylenediamine (0.8 g, 3.87 mmol) and sodium triacetoxyborohydride (2.46 g, 11.63 mmol) in 5:3 *v*/*v* dichloromethane/glacial acetic acid (10 mL). The reaction mixture was left to stir at room temperature for 18 h. The mixture was washed with water and brine and then extracted into ethyl acetate. The organic layer was dried over sodium sulphate and chromatographed on silica gel to yield the desired product as a yellow solid (0.6 g, 36%). ^1^H NMR (300 MHz, CDCl_3_) δ 1.54 (s, 9H, 3 × CH_3_), δ 4.50 (s, 2H, CH_2_), δ 6.51 (s, 1H, NH), δ 7.39–7.42 (m, 2H, ArH), δ 7.54–7.59 (m, 2H, ArH). ^13^C NMR (75.5 MHz, CDCl_3_) δ 28.3 (3 × CH_3_), 57.2 (CH_2_), 80.8 (C-O), 119.2 (2 × ArCH), 119.9 (ArC), 121.5 (ArC), 132.9 (2 × ArCH), 133.3 (CBr), 135.5 (CBr), 152.6 (C=O), 163.1 (C=O); IR (ATR): υ_max_ 3348, 3099, 2973, 1768, 1688, 1605, 1518, 1430, 1364, 1283, 1232, 1146, 1020, 846, 739, 680 cm^−1^; HRMS (ESI) *m*/*z* calcd. for C_15_H_16_Br_2_N_2_O_3_Na 452.9420 [M + Na]^+^, found 452.9422.

(21) *N*-(4-aminophenyl)-3,4-dibromo-1,5-dihydro-2*H*-pyrrol-2-one (**29**)


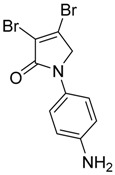


The title compound was synthesized by following the same method used to synthesize Compound **19** to afford a yellow solid (0.13 g, 28%). m.p. 163 °C; ^1^H NMR (300 MHz, DMSO-*d*_6_) δ 4.70 (s, 2H, CH_2_), δ 5.16 (brs, 1H, NH_2_), δ 6.55–6.6 (m, 2H, ArH), 7.25–7.28 (m, 2H, ArH); ^13^C NMR (75.5 MHz, DMSO-*d*_6_) δ 57.0 (CH_2_), 115.4 (2 × ArCH), 121.6 (2 × ArCH), 127.0 (ArC), 129.4 (ArC), 132.4 (CBr), 144.2 (CBr), 164.2 (C=O); IR (ATR): υ_max_ 3305, 3208, 2920, 2287, 1693, 1611, 1512, 1442, 1383, 1280, 1150, 1023, 900, 830 cm^−1^; UV (ACN): λ_max_ 243 (ε 8973 cm^−1^M^−1^), 306 (3430); HRMS (ESI) *m*/*z* calcd. for C_10_H_9_Br_2_N_2_O 330.9076 [M + H]^+^, found 330.9075.

(22) 3,4-dichloro-1-(4-hydroxyphenethyl)-1,5-dihydro-2*H*-pyrrol-2-one (**30**)


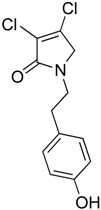


The title compound was prepared according to Procedure A from mucochloric acid (700 mg, 4.14 mmol) and tyramine (568.4 mg, 4.14 mmol) and was left to react for 3 days. The crude mixture was poured into icy water, and the solid was collected and washed twice with ether. A light green solid was obtained (280 mg; 25%). m.p. 157.8 °C; ^1^H NMR (CDCl_3_, 300 MHz): *δ* 2.72 (t, *J* = 7.2 Hz, 2H, CH_2_), *δ* 3.55 (t, *J* = 7.2 Hz, 2H, CH_2_), *δ* 4.2 (s, 2H, C5-CH_2_), 6.67 (d, *J* = 8.4 Hz, 2H, ArH), *δ* 7.00 (d, *J* = 8.4 Hz, 2H, ArH), 9.22 (s, 1H, OH). ^13^C NMR (DMSO, 75.5): 33.3 (CH_2_), 44.6 (CH_2_), 53.7 (C5-CH_2_), 115.7 (ArCH), 124.1 ©, 129.0 (C), 130.0 (ArCH), 140.9 (C), 156.3 (C), 163.4 (C=O); IR (ATR): υ_max_ 3237.6, 1670.5, 1513.3, 1451.15, 1279.3, 1219.1, 1169.4, 827.6; UV (MeOH): λ_max_ 277.0 nm (ε 19075.9 cm^−1^M^−1^), 320 (2585.1); HRMS (ESI) *m*/*z* calcd. for C_12_H_11_Cl_2_N_1_O_2_Na_1_294.0059 [M + Na]^+^, found 294.0060.

(23) 1-(2-(1*H*-indol-3-yl)ethyl)-3,4-dichloro-1,5-dihydro-2*H*-pyrrol-2-one (**31**)


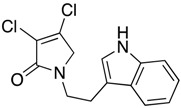


The title compound was prepared according to Procedure A from mucochloric acid (200 mg, 1.18 mmol) and tryptamine (189.7 mg, 1.18 mmol) and was left to react for 24 h. The crude mixture was extracted with dichloromethane (30 mL) and washed with water (15 mL) followed by brine (15 mL), dried over sodium sulphate, and the solvent was evaporated. The oil residue was then triturated with methanol, and the precipitated solid was filtered to yield a green solid (145 mg; 42%). m.p. 159.3 °C; ^1^H NMR (DMSO, 400 MHz,): *δ* 2.96 (t, *J* = 7.3 Hz, CH_2_), 3.67 (*t*, *J* = 7.3 > N-CH_2_), 7.07 (s, 1H, ArH), *δ* 7.16 (t, *J* = 7.7, 14.8 Hz, 1H, ArH), *δ* 7.22 (t, *J* = 7.0, 14.8 Hz, 1H, ArH), *δ* 7.40 (d, *J* = 8.1 Hz, 1H), *δ* 7.61 (d, *J* = 8.1 Hz, 1H, ArH), 10.84 (1H, NH). ^13^C NMR (DMSO, 100 MHz): *δ* 24.2 (CH_2_), 43.6 (CH_2_), 54.0 (C5-CH_2_), 111.3 ©, 111.9 (ArCH), 118.6 (ArCH), 118.8 (ArCH), 121.5 (ArCH), 123.3 (ArCH), 124.2 (C), 127.5 (ArC), 136.7 (ArC), 140.9 (C), 163.5 (C=O); IR (ATR): υ_max_ 3277.5, 3058.0, 2918.1, 1772.8, 1678.8, 1452.1, 1364.8, 1298.4, 1099.1, 1036.7, 974.5, 852.9, 738.8; UV (MeOH): λ_max_ 275.0 nm (ε 3463.2 cm^−1^M^−1^), 220.0 nm (15204.8); HRMS (ESI) *m*/*z* calcd. for C_14_H_12_Cl_2_N_2_O_1_Na_1_ 317.02189 [M + Na]^+^, found 317.02188.

(24) *N*-butyl-4-(3,4-dichloro-2-oxo-2,5-dihydro-1*H*-pyrrol-1-yl)benzamide (**32**)


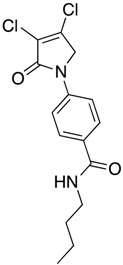


The title compound was prepared from acid **19** (200 mg, 0.74 mmol) and butylamine (53.8 mg, 0.74 mmol) according to the general Procedure B. The solvent of the reaction mixture was evaporated then triturated with methanol, and the precipitated solid was filtered. A beige solid was obtained (40 mg; 17%). m.p. 168.5 °C; ^1^H NMR (DMSO-*d*_6_, 600 MHz): *δ* 0.99 (t, *J* = 7.4 Hz, 3H, CH_3_), 1.23–1.34 (m, 4H, 2 × CH_2_), 1.50 (m, 2H, CH_2_), 4.89 (s, 2H, C5-CH_2_), 7.80 (d, *J* = 8.9 Hz, 2H, ArH), 7.89 (d, *J* = 8.9 Hz, 2H, ArH), 8.41 (s, 1H, NH). ^13^C NMR (DMSO-*d*_6_, 150.9 MHz): *δ* 14.11 (CH_3_), 20.11 (CH_2_), 31.71 (CH_2_), 53.92 (C5-CH_2_), 118.26 (ArCH), 124.2 (C), 129.60 (ArCH), 130.8 (C), 141.0 (C), 142.5 (C), 162.7 (C=O), 165.7 (C=O); IR (ATR): υ_max_ 3314.7, 3078.0, 2955.3, 1696.7, 1606.9, 1508.1, 1381.5, 1152.3, 835.7, 763.2; UV (MeOH): λ_max_ 300.0 nm (ε 10,897.6 cm^−1^M^−1^); HRMS (ESI) *m*/*z* calcd. for C_15_H_16_Cl_2_N_2_O_2_Na_1_ 349.0481 [M + Na]^+^, found 349.0482.

(25) 4-(3,4-dichloro-2-oxo-2,5-dihydro-1*H*-pyrrol-1-yl)-*N*-hexylbenzamide (**33**)


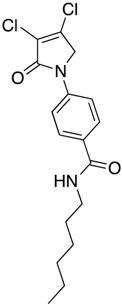


The title compound was prepared from acid **19** (200 mg, 0.74 mmol mmol) and hexylamine (74.4 mg, 0.74 mmol) according to the general Procedure B. The solvent of the reaction mixture was evaporated then triturated with methanol, and the precipitated solid was filtered. A white solid was obtained (75 mg; 29%); m.p. 159.5 °C; ^1^H NMR (DMSO-*d*_6_, 400 MHz): *δ* 0.88 (t, *J* = 6.9 Hz, 3H, CH_3_), 1.18–1.21 (m, 6H, 3 × CH_2_), 1.28–1.53 (m, 2H, CH_2_), 3.06–3.09 (m, 2H, CH_2_), 4.90 (s, 2H, CH_2_), 7.81 (d, *J* = 8.4 Hz, 2H, ArH), 7.90 (d, *J* = 8.4 Hz, 2H, ArH), 8.43 (s, NH). ^13^C NMR (DMSO-*d*_6_, 100 MHz): *δ* 22.5 (CH_3_), 26.7 (CH_2_), 29.6 (CH_2_), 31.5 (CH_2_), 39.7 (CH_2_), 45.9 (CH_2_), 54.0 (C5-CH_2_), 120.0 (ArCH), 124.2 (C), 128.6 (ArCH), 130.8 (C), 140.9 (C), 142.4 (C), 162.7 (C=O, 165.7 (C=O). IR (ATR): υ_max_ 3331.8, 2929.2, 1691.9, 1630.4, 1503.6, 1377.3, 1270.6, 1151.8, 927.3, 846.7; UV (MeOH): λ_max_ 280.0 nm (ε 6522.5 cm^−1^M^−1^); HRMS (ESI) *m*/*z* calcd. for C_17_H_20_Cl_2_N_2_O_2_Na_1_ 377.0794 [M + H]^+^, found 377.0792.

(26) *N*-benzyl-4-(3,4-dichloro-2-oxo-2,5-dihydro-1*H*-pyrrol-1-yl)benzamide (**34**)


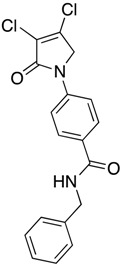


The title compound was prepared from 4-(3,4-dichloro-2-oxo-2,5-dihydro-1*H*-pyrrol-1-yl)benzoic acid (**19**) (200 mg, 0.74 mmol mmol) and benzylamine (117.8 mg, 1.1 mmol) according to the general Procedure B. The precipitated solid was collected by filtration and washed with 1 mL of water, followed by hexane, then triturated from methanol. A white solid was obtained (230 mg; 76%). m.p. 175.3 °C; ^1^H NMR (DMSO-*d*_6_, 400 MHz): *δ* 3.96 (s, 2 H, CH_2_), 4.90 (s, 2H, C5-CH_2_), 7.21–7.46 (m, 6H, ArH and NH), 7.80 (d, *J* = 8.8 Hz, 2H, ArH), 7.96 (d, *J* = 8.8, 2H, ArH). ^13^C NMR (DMSO-*d*_6_, 400 MHz): *δ* 54.0 (C5-CH_2_), 67.5 (CH_2_), 118.2 (ArCH), 124.2 (C), 127.7 (ArC), 128.3 (ArC), 128.4 (ArCH), 128.7 (ArCH), 128.9 (ArCH), 130.8 (C), 142.4 (C), 162.8 (C=O), 167.8 (C=O). IR (ATR): υ_max_ 2824.5, 2641.0, 1699.5, 1604, 1510.0, 1366.2, 1305.9, 1152.1, 782.0; UV (MeOH): λ_max_ 285.0 nm (ε 3272.6 cm^−1^M^−1^); HRMS (ESI) *m*/*z* calcd. for C_18_H_15_Cl_2_N_2_O_2_ 361.0505 [M + H]^+^, found 361.0501. 

(27) Synthesis of *N*-(2-(1*H*-indol-3-yl)ethyl)-4-(3,4-dichloro-2-oxo-2,5-dihydro-1*H*-pyrrol-1-yl)benzamide (**35**)


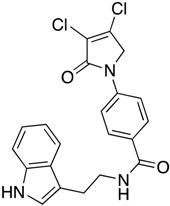


The title compound was prepared from 4-(3,4-dichloro-2-oxo-2,5-dihydro-1*H*-pyrrol-1-yl)benzoic acid (**19**) (300 mg, 1.10 mmol) and tryptamine (176.7 mg, 1.1 mmol) according to the general Procedure B. The precipitated solid was collected by filtration and washed with (1 mL) water, followed by hexane, then the solid was triturated from methanol to yield a pale pink solid (400 mg, 88%); m.p. 149.8 °C; ^1^H NMR (DMSO-*d*_6_, 400 Hz): *δ* 2.52 (m, 2H, CH_2_), 3.32 (m, 2H, CH_2_), 4.88 (S, 2H, C5-CH_2_), 6.99 (t, *J* = 7.0 Hz, 1H, ArH), *δ* 7.08 (t, *J* = 7.0 Hz, 1H, ArH)), *δ* 7.22 (s, 1H, NH), *δ* 7.36 (d, *J* = 8.0 Hz, 1H, ArH), *δ* 7.55 (d, *J* = 8.0 Hz, 1H, ArH), 7.75 (d, *J* = 8.8 Hz, 2H, ArH), 7.94 (d, *J* = 8.8 Hz, 2H, ArH), 10.94 (s, CONH). ^13^C NMR (DMSO-*d*_6_, 100 MHz): *δ* 23.4 (CH_2_), 46.1 (CH_2_), 53.8 (CH_2_), 83.01 (ArC), 109.82 (ArCH), 112.04 (ArCH), 118.53 (ArCH), 119.03 (ArCH), 120.15 (ArCH), 121.7 (ArCH), 123.8 ©, 130.6 (ArCH), 136.7 (ArCH), 141.2 (ArC), 142.37 (ArC), 142.85 (ArC), 153.0 (C), 166.35 (C=O), 167.4 (C=O); IR (ATR): υ_max_ 3223.4, 2902.4, 1700.1, 1603.1, 1370.0, 1248, 1038.6, 843.4, 781.0, UV (MeOH): λ_max_ 280.0 nm (ε 3802.5 cm^−1^M^−1^); HRMS (ESI) *m*/*z* calcd. for C_21_H_17_Cl_2_N_3_O_2_Na_1_ 436.0590 [M + Na]^+^, found 436.0583.

(28) 4-(3,4-dichloro-2-oxo-2,5-dihydro-1*H*-pyrrol-1-yl)-*N*-(2-morpholinoethyl)benzamide (**36**)


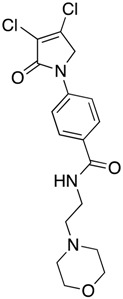


The title compound was prepared from 4-(3,4-dichloro-2-oxo-2,5-dihydro-1*H*-pyrrol-1-yl)benzoic acid (**19**) (200 mg, 0.74 mmol mmol) and 2-morpholinoethan-1-amine (95.7 mg, 0.74 mmol) according to Procedure B. After the completion of the reaction, the crude mixture was dissolved in ethanol, and few drops of diethyl ether were added dropwise. Then, the black solid impurity was filtered and discarded. The yellow filtrate was evaporated to dryness then recrystallized from ethanol, and a yellow small needle crystal was obtained (7 mg; 3%). m.p. 129.1 °C; ^1^H NMR (DMSO-*d*_6_, 600 MHz): *δ* 3.05–3.13 (m, 4H, 2 X CH_2_), 3.55 (d, *J* = 12 Hz, 2H, CH_2_), 3.68 (d, *J* = 6.0 Hz, 2H, CH_2_), 3.79 (t, *J* = 12.0 Hz, 2H, CH_2_), 3.98 (d, *J* = 12.0, 2H, CH_2_), 4.91 (s, 2H, C5-CH_2_), 7.84 (d, *J* = 8.6, 2H, ArH), 7.98 (d, *J* = 8.6 Hz, 2H, ArH), 8.87 (s, NH). ^13^C NMR (DMSO-*d*_6_, 100 MHz): *δ* 34.2 (CH_2_), 46.0 (CH_2_), 51.7 (CH_2_), 54.0 (CH_2_), 63.6 (CH_2_), 118.3 (ArCH), 124.2 ©, 129.0 (ArCH), 131.0 (C), 141.4 (C), 142.7 (C), 162.8 (C=O); IR (ATR): υ_max_ 3307.4, 2923.8, 1699.1, 1603.7, 1509.0, 1370.2, 1110.5, 782.0; UV (MeOH): λ_max_ 285.0 nm (ε 6378.6 cm^−1^M^−1^); HRMS (ESI) *m*/*z* calcd. for C_17_H_20_Cl_2_N_3_O_3_ 384.0876 [M + H]^+^, found 384.0879.

(29) 4-(3,4-dichloro-2-oxo-2,5-dihydro-1*H*-pyrrol-1-yl)-*N*-(3-morpholinopropyl)benzamide (**37**)


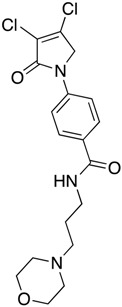


The title compound was prepared from 4-(3,4-dichloro-2-oxo-2,5-dihydro-1*H*-pyrrol-1-yl)benzoic acid (**19**) (300 mg, 1.1 mmol mmol) and 3-morpholinopropan-1-amine (160 mg, 1.1 mmol) according to the general Procedure B. The precipitated solid was filtered, washed with ether and hexane to yield a light beige solid (180 mg; 50%). m.p. 129.1 °C; ^1^H NMR (DMSO-*d*_6_, 400 MHz): *δ* 1.70–1.74 (m, 2H, CH_2_), 2.34–2.41 (m, 10 H, 5 × CH_2_), 2.53 (t, *J* = 5.4, 10.0, 2H, CH_2_), 4.88 (s, 2H, C5-CH_2_), 7.72 (d, *J* = 8.8, 2H, ArH), 7.91 (d, *J* = 8.8, 2H ArH and NH), ^13^C NMR (DMSO-*d*_6_, 100 MHz): *δ* 23.91 (CH_2_), 38.8 (CH_2_), 53.3 (CH_2_), 55.97 (CH_2_), 66.34 (CH_2_), 117.9 (ArCH), 124.8 ©, 129.9 (ArCH), 134.2 (ArC), 139.9 (C), 141.44 (C), 163.18 (C=O), 173.09 (C=O). IR (ATR): υ_max_ 3372.1, 3078.2, 2934.0, 1695.2, 1531.4, 1370.7, 1110.8, 1042.2, 778.6; UV (MeOH): λ_max_ 285.0 nm (ε 2695.1 cm^−1^M^−1^); HRMS (ESI) *m*/*z* calcd. for C_18_H_22_Cl_2_N_3_O_3_ 398.1033 [M + H]^+^, found 398.1030.

(30) 4-(3,4-dichloro-2-oxo-2,5-dihydro-1*H*-pyrrol-1-yl)-*N*-(3-(piperidin-1-yl)propyl)benzamide (**38**)


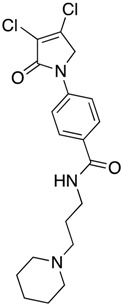


The title compound was prepared from 4-(3,4-dichloro-2-oxo-2,5-dihydro-1*H*-pyrrol-1-yl)benzoic acid (**19**) (200 mg, 0.74 mmol mmol) and 3-(piperidin-1-yl)propan-1-amine (104.6 mg, 0.74 mmol) according to the general Procedure B. The solid product was collected by filtration, washed with water and hexane to yield a beige solid (64 mg; 27%). m.p. 165.5 °C; ^1^H NMR DMSO-*d*_6_, 600 MHz): *δ* 1.24–1.38 (m, 8H, 4 × CH_2_), 1.46–1.48 (m, 4H, 2 × CH_2_), 1.98–2.00 (m, 2H, CH_2_), 2.01–2.3 (m, 2H, CH_2_), 4.90 (s, 2H, C5-CH_2_), 5.32 (s, NH), 7.79 (d, *J* = 8.2 Hz, 2H, ArH), 7.95 (d, *J* = 8.2 Hz, 2H, ArH). ^13^C NMR (DMSO-*d*_6_, 100 MHz): *δ* 22.3 (CH_2_), 25.5 (CH_2_), 26.4 (CH_2_), 29.1 (CH_2_), 31.3 (CH_2_), 39.7 (CH_2_), 46.4 (CH_2_), 118.1 (ArCH), 124.8 ©, 128.0 (ArC), 129.5 (ArCH), 130.5 (C), 140.9 (C), 163.2 (C=O), 167.8 (C=O). IR (ATR): υ_max_ 3355.8, 2936.3, 16887.5, 1540.6, 1375.5, 1042.7, 850.0, 775.0; UV (MeOH): λ_max_ 285.0 nm (ε 7022.6 cm^−1^M^−1^); HRMS (ESI) *m*/*z* calcd. for C_19_H_24_Cl_2_N_3_O_2_ 396.1240 [M + H]^+^, found 396.1234.

(31) *N*-(4-(3,4-dichloro-2-oxo-2,5-dihydro-1*H*-pyrrol-1-yl)phenyl)butyramide (**39**)


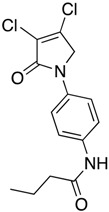


The title compound was prepared from 1-(4-aminophenyl)-3,4-dichloro-1,5-dihydro-2*H*-pyrrol-2-one (**23**) (200 mg, 0.82 mmol) and butyryl chloride (87.7 mg, 0.82 mmol) according to the general Procedure C. After completion of the reaction, the crude mixture was evaporated to complete dryness, followed by trituration from methanol, then washed with 1 mL of ether twice; the solid was collected by filtration and dried in high vac. An off-white solid was obtained (89 mg, 31%). m.p. 195.5 °C; ^1^H NMR (DMSO-*d*_6_, 400 MHz): *δ* 0.91 (t, *J* = 7.3 Hz, 3H, CH_3_), 1.58–1.64 (m, 2H, CH_2_), 2.26 (t, *J* = 7.3 Hz, 2H), 4.82 (s, 2H, C5-CH_2_), 7.62 (brs, 4 H, ArH), 9.92 (s, NH). ^13^C NMR (DMSO-*d*_6_, 100 MHz): *δ* 14.1 (CH_2_), 19.0 (CH_2_), 54.1 (CH_2_), 120.0 (ArCH), 120.1 (ArCH), 124.3 ©, 134.0 (C), 136.6 (C), 141.6 (C), 162.2 (C), 171.5 (C=O); IR (ATR): υ_max_ 3343.2, 2961.6, 1677.3, 1522.0, 1408.4, 1283.0, 820.6; UV (MeOH): λ_max_ 290.0 nm (ε 8377.5 cm^−1^M^−1^); HRMS (ESI) *m*/*z* calcd. for C_14_H_24_Cl_2_N_2_O_2_Na_1_ 335.0325 [M + Na]^+^, found 335.0323.

(32) *N*-(4-(3,4-dichloro-2-oxo-2,5-dihydro-1*H*-pyrrol-1-yl)phenyl)hexanamide (**40**)


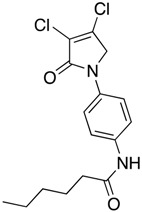


The title compound was prepared from 1-(4-aminophenyl)-3,4-dichloro-1,5-dihydro-2*H*-pyrrol-2-one (**23**) (200 mg, 0.82 mmol) and hexanoyl chloride (110.7 mg, 0.82 mmol) according to Procedure C. After completion of the reaction, the crude mixture was evaporated to complete dryness, followed by trituration from methanol, then the solid was washed with 1 mL of ether twice. The solid was collected by filtration and dried in high vac. An off-white solid was obtained (164 mg; 52%). m.p. 225.7 °C; ^1^H NMR (DMSO-*d*_6_, 300 MHz): *δ* 0.93 (t, *J* = 7.0 Hz, 3H, CH_3_), 1.38 (d, *J* = 3.6 Hz, 4H, 2 × CH_2_), 1.73–1.79 (m, 2H, CH_2_), 2.38 (t, *J* = 7.47 Hz, 2H, CH_2_), 4.49 (s, 2H, C5-CH_2_), 7.18 (s, NH), 7.59 (brs, 4H, ArH). ^13^C NMR (CDCl_3_, 75.5 MHz): 14.3 (CH_2_), 22.4 (CH_2_), 25.2 (CH_2_), 31.4 (CH_2_), 36.8 (CH_2_), 54.1 (CH_2_), 119.9 (ArCH), 120.1 (ArCH), 124.3 (C), 133.8 (C), 136.7 (C), 141.6 (C), 162.2 (C), 171.7 (C); IR (ATR): υ_max_ 3358.6, 2949.4, 1677.3, 1515.9, 1413.1, 1515.9, 1284.3, 1042.8, 853.0; UV (MeOH): λ_max_ 290.0 nm (ε 8394.3 cm^−1^M^−1^); HRMS (ESI) *m*/*z* calcd. for C_16_H_18_Cl_2_N_2_O_2_Na_1_ 363.0638 [M + Na]^+^, found 363.0636.

(33) *N*-(4-(3,4-dichloro-2-oxo-2,5-dihydro-1*H*-pyrrol-1-yl)phenyl)octanamide (**41**)


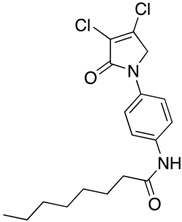


The title compound was prepared from 1-(4-aminophenyl)-3,4-dichloro-1,5-dihydro-2*H*-pyrrol-2-one (**23**) (200 mg, 0.82 mmol) and octanoyl chloride (133.8 mg, 0.82 mmol) according to Procedure C. After completion of the reaction, the crude mixture was evaporated to complete dryness, followed by trituration from methanol and then washed with 1 mL of ether twice. The solid was collected by filtration and dried in high vac. A white solid was obtained (140 mg; 44%). m.p. 194.1 °C; ^1^H NMR (CDCl_3_, 300 MHz): 0.91 (t, *J* = 6.8 Hz, 3H, CH_3_), 1.31–1.40 (m, 4H, 2 × CH_2_), 1.58 (brs, 4H, 2 × CH_2_), 1.75 (m, 2 H, CH_2_), 2.38 (t, *J* = 7.7 Hz, 2H, CH_2_), 4.50 (s, 2H, C5-CH_2_), 7.24 (s, NH), 7.58 (brs, 4H, ArH). ^13^C NMR (CDCl_3_, 75.5 MHz): 14.4 (CH_2_), 22.5 (CH_2_), 25.6 (CH_2_), 28.9 (CH_2_), 29.1 (CH_2_), 31.6 (CH_2_), 36.8 (CH_2_), 54.1 (CH_2_), 119.9 (ArCH), 120.1 (ArCH), 124.3 (C), 133.8 (C), 136.7 (C), 141.6 (C), 162.2 (C), 171.7 (C=O); IR (ATR): υ_max_ 3338.9, 2915.7, 1678.1, 1521.3, 1285.3, 1156.1, 931.4, 822.9; UV (MeOH): λ_max_ 290.0 nm (ε 5982.4 cm^−1^M^−1^); HRMS (ESI) *m*/*z* calcd. for C_18_H_22_Cl_2_N_2_O_2_Na_1_ 391.0951 [M + Na]^+^, found 391.0952.

(34) 2-(4-bromophenyl)-*N*-(4-(3,4-dichloro-2-oxo-2,5-dihydro-1*H*-pyrrol-1-yl)phenyl)acetamide (**42**)


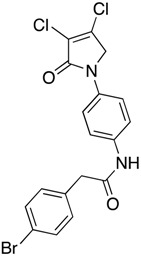


The title compound was prepared according to method C from amine 1-(4-aminophenyl)-3,4-dichloro-1,5-dihydro-2*H*-pyrrol-2-one (**23**) (200 mg, 0.82 mmol) and 2-(4-bromophenyl)acetic acid (110.7 mg, 0.82 mmol). The latter was first converted to the corresponding acid chloride prior to amide coupling by treatment with thionyl chloride (2 h) on reflux, followed by complete dryness in high vac. After completion of the reaction, the crude mixture was evaporated to complete dryness, followed by trituration from methanol, and the obtained solid was then washed twice with 1 mL of ether. The solid was collected by filtration and dried in high vac. A white solid (100 mg; 32%) was obtained; m.p. 159.2 °C; ^1^H NMR (CDCl_3_, 300 MHz): 3.64 (s, 2H, CH_2_), 4.82 (s, 2H, C5-CH_2_), 7.30 (d, *J* = 8.2 Hz, 2H, ArH), 7.52 (d, *J* = 8.2 Hz, 2H, ArH), 7.63 (brs, 4H, ArH), 10.33 (s, NH). ^13^C NMR (CDCl_3_, 75.5 MHz): 45.95 (CH_2_), 54.09 (CH_2_), 120.06 (ArCH), 120.21 (ArCH), 124.3 (C), 131.6 (ArCH), 131.9 (ArCH), 134.1 (C), 135.85 (C), 136.40 (C), 141.7 (C), 162.3 (C), 169.0 (C); IR (ATR): υ_max_ 3279.8, 2978.7, 1690.5, 1516.5, 1379.5, 1153.7, 927.8, 824.9; UV (MeOH): λ_max_ 285.0 nm (ε 3375.7 cm^−1^M^−1^); HRMS (ESI) *m*/*z* calcd. for C_18_H_13_Br_1_Cl_2_N_2_O_2_Na_1_ 460.9430 [M + Na]^+^, found 460.9431.

### 4.3. Biology

#### 4.3.1. Quorum Sensing Inhibition Assay for PAMH602

The *P. aeruginosa* MH602 PlasB::gfp (ASV) reporter strain was used. An overnight culture was prepared in Luria-Bertani (LB10) media supplemented with gentamycin (40 µM). This bacterial culture solution was diluted (1 in 100) with LB10 supplemented with gentamycin (15 µM). Stock solutions of the synthesized compounds were prepared at 20 mM in DMSO. Compounds were pipetted into each well with final concentrations of 250 µM, 125 µM and 62.5 µM (in triplicate) with a final volume of 200 µL with the prepared bacterial culture. The negative control was prepared containing 200 µL of the bacterial culture without the tested compounds. The halogenated furanone (Fu-30) **5** and TP-5 were used as positive controls. The plates were incubated at 37 °C for 15 h. The plates were measured for GFP expression (fluorescence: excitation 485 nm, emission 535 nm) using a microplate reader (Wallac Victor, Perkin-Elmer), and the cell growth was also assessed by recording the OD at 600 nm.

#### 4.3.2. Pyocyanin Assay

An overnight culture of *P. aeruginosa* PAO1 was diluted 1 in 100 with LB10 medium. To a 5-mL test tube were added the tested compounds (compounds prepared from a DMSO stock of 20 mM), and 2.5 mL of the prepared culture was added, giving final concentrations of 250 µM and 32 µM. An equivalent amount of DMSO was prepared for the positive control containing the bacterial culture without the tested compounds. The same medium without bacterial culture in an equivalent amount of DMSO was used as the background reading. The cultures were grown with shaking at 37 °C for 17 h. The final cell density was measured by reading the absorbance at 600 nm (OD_600_), and the solutions were then centrifuged for 5000 rpm. The clear supernatant was then transferred by pipetting into a plastic 96-well plate, and the absorbance was measured at 695 nm.

### 4.4. Docking

The crystal structure of LasR complexed with OdDHL (PDB: 2UV0) was used. The protein was prepared before docking by removing the other subunits, and only subunit E was used for docking based on a previous control docking. The binding site and cavity were prepared as follows. Water molecules and ligands including the LasR bound ligand in the binding pocket were removed, and the crystal structure was protonated. The binding pocket was chosen from the ‘receptor cavities’ tool, and this defined the binding site sphere in the proposed pocket. Alternate conformers were identified (Ser20 and Ser131), but were outside the binding pocket sphere. The geometry of the protonated OdDHL ligand was optimized with the CHARMM force field [28], and it was docked back to the prepared protein using the Genetic Optimization for Ligand Docking (GOLD) algorithm, Versions 5.2.1, 5.2.2, and 5.4.0 (Cambridge Crystallographic Data Centre, U.K.) [29]. The number of the docking runs was set to 100, while the ‘detect cavity’ and ‘early determination’ were set to ‘false’, but all other parameters were set to their defaults. The GoldScore was specified as the fitness score function. After the docking run, ligand poses were analysed based on their clusters using the RMSD of heavy atoms and within each cluster; poses were ranked in order of decreasing GoldScore value. The largest clusters at RMSD of around 2 Å were usually considered. The best pose of the largest cluster for OdDHL when docked back to the prepared protein was selected. The ligand from the crystal structure was superimposed with the selected pose, and an acceptable RMSD was obtained of 0.94 Å (heavy atoms).

The tested compounds were sketched, protonated and their energy minimized as above with the CHARMm force field [28]. Docking was then performed and analysed as described above. Receptor ligand interactions of selected poses were analysed using the ‘view interactions’ tool. Different types of interactions including hydrogen bonds, hydrophobic and π-interactions were examined and compared to the crystal structure. Any other attractive forces, repulsive or unfavourable interactions were noted.

## Figures and Tables

**Table 1:** Percentage inhibition of GFP fluorescence and the reduction of OD by the synthesized lactams against *P. aeruginosa* MH602.

QSI Activity against *P. aeruginosa* MH602			
Concentration (µM)			
Compound	250	125	62.5
**9**	^b^ 71.4 ± 1.0	^a^ 51.0 ± 1.8	^a^ 47.8 ± 0.8
**10**	^b^ 68.0 ± 0.5	^a^ 50.6 ± 0.5	^a^ 44.3 ± 7.0
**11**	^b^ 62.1 ± 3.4	^a^ 39.4 ± 2.5	^a^ 38.2 ± 5.0
**12**	^b^ 83.0 ± 2.8	^a^ 82.0 ± 2.6	^a^ 69.4 ± 0.3
**13**	^b^ 78.3 ± 2.3	^a^ 74.6 ± 0.8	^a^ 65.7 ± 0.3
**14**	^a^ 58.0 ± 3.3	^a^ 47.8 ± 1.3	^a^ 35.2 ± 5.7
**15**	^a^ 35.7 ±1.8	^a^ 25.92 ± 2.5	^a^ 15.35 ± 0.8
**16**	^a^ 19.8 ± 3.3	^a^ 13.25 ± 3.9	^a^ 6.85 ± 1.8
**19**	^a^ 80.4 ± 1.1	^a^ 79.5 ± 0.6	^a^ 78.1 ± 1.6
**20**	^a^ 58.82 ± 5.8	^a^ 36.97 ± 3.2	^a^ 22.82 ± 3.2
**21**	^a^ 17.23 ± 3.0	^a^ 13.5 ± 3.9	^a^ 9.27 ± 2.3
**22**	^a^ 76.4 ± 1.2	^a^ 60.5 ± 0.5	^a^ 48.2 ± 2.0
**23**	^b^ 81.7 ± 0.1	^a^ 78.7 ± 0.5	^a^ 76.5 ± 0.4
**24**	^b^ 80.7 ± 3.0	^a^ 77.5 ± 4.1	^a^ 69.7 ± 0.9
**25**	^a^ 55.8 ± 1.2	^a^ 54.2 ± 2.8	^a^ 37.1 ± 1.6
**26**	^a^ 28.17 ± 3.1	^a^ 16.25 ± 1.9	^a^ 9.23 ± 1.1
**27**	^a^ 13.33 ± 3.0	^a^ 6.2 ± 3.5	NA
**29**	^a^ 42.55 ± 3.3	^a^ 26.15 ± 3.1	^a^ 13.55 ± 2.6
**30**	^a^ 74.0 ± 1.7	^a^ 43.1 ± 1.1	^a^ 32.1 ± 3.5
**31**	^a^ 43.6 ± 6.5	^a^ 40.2 ± 6.2	^a^ 33.5 ± 2.8
**32**	^a^ 52.5 ± 6.2	^a^ 41.7 ± 4.1	NA
**33**	^a^ 32.3 ± 4.6	^a^ 3.5 ± 5.8	NA
**34**	^a^ 54.2 ± 5.9	^a^ 30.9 ± 6.0	^a^ 25.3 ± 1.0
**35**	^a^ 53.5 ± 5.6	^a^ 28.7 ± 3.2	^a^ 14.4 ± 4.7
**36**	^a^ 33.5 ± 0.5	^a^ 20.8 ± 2.1	NA
**37**	^a^ 37.1 ± 2.1	^a^ 29.1 ± 2.7	^a^ 4.0 ± 1.88
**38**	^a^ 30.0 ± 3.9	^a^ 21.5 ± 1.6	NA
**39**	^a^ 41.4 ± 2.8	^a^ 32.2 ± 3.7	^a^ 16.7 ± 3.9
**40**	^a^ 36.0 ± 3.0	^a^ 31.7 ± 2.0	^a^ 4.6 ± 0.7
**41**	^a^ 53.6 ± 3.1	^a^ 32.7 ± 4.0	^a^ 16.9 ± 5.5
**42**	^a^ 34.5 ± 2.0	^a^ 30.2 ± 4.4	^a^ 12.2 ± 2.9
^d^ **TP-5**	^a^ 34.4 ± 5.2	^a^ 30.4 ± 3.2	^a^ 2.3 ± 0.3
^d^ **Fu-30 (5)**	^c^ 84.8 ± 2.9	^c^ 82.5 ± 2.5	^c^ 74.4 ± 3.6

^a^ Bacterial growth inhibition ≤15%; ^b^ growth inhibition between 15 and 30%; ^c^ growth inhibition greater than 30%; ^d^ positive control; ± the standard deviation of the mean for three independent experiments. In each independent experiment, compounds were tested in triplicate. NA = no activity and no growth inhibition (supplementary materials Table S1).

## Supplementary Materials

The growth inhibition, full docking and spectral data are provided in the Supplementary.
