# Supplementary material for: Design and Synthesis of Lactams Derived from Mucochloric and Mucobromic Acids as Pseudomonas aeruginosa Quorum Sensing Inhibitors
Source: Molecules. 2018 May 7;23(5):1106. doi: 10.3390/molecules23051106 (PMC6100351; doi:10.3390/molecules23051106)

Supplementary Materials

Design and Synthesis of Lactams Derived from Mucochloric and Mucobromic Acids as *Pseudomonas aeruginosa* Quorum Sensing Inhibitors

Basmah Almohaywi[1,2], Aditi Taunk[1], Daniel S. Wenholz[1], Shashidhar Nizalapur[1], Nripendra Nath Biswas[1], Kitty K. K. Ho[1], Scott A. Rice[3], George Iskander[1], David Black[1], Renate Griffith[4] and Naresh Kumar[*1]

Table of Contents

Table S1. Growth inhibition (OD 600 reduction) at different concentrations. 3

Table S2. Docking of lactams to the LasR receptor protein of *P. aeruginosa*. 4

Spectra Data ^{1}H NMR and ^{13}C NMR Spectra 6

2) 3,4-dichloro-1-hexyl-1,5-dihydro-2*H*-pyrrol-2-one (Compound 10) 7
3) 3,4-dichloro-1-(2-hydroxyphenyl)-1,5-dihydro-2H-pyrrol-2-one (Compound 12) 8
4) 3,4-dichloro-1-(3-hydroxyphenyl)-1,5-dihydro-2*H*-pyrrol-2-one (Compound 13) 9
5) 3,4-dichloro-1-(4-hydroxyphenyl)-1,5-dihydro-2*H*-pyrrol-2-one (Compound 14) 10
6) N-(3-carboxyphenyl)-3,4-dibromo-1,5-dihydro-2H-pyrrol-2-one (DHP phenyl acid-2) (Compound 15) 11
7) N-(4-carboxyphenyl)-3,4-dichloro-1,5-dihydro-2H-pyrrol-2-one (DHP phenyl acid-1) (Compound 16) 12
8) N-(3-tert-butylphenylcarbamate)-3,4-dichloro-1,5-dihydro-2H-pyrrol-2-one (Compound 17) 13
9) N-(4'-tert-butylphenylcarbamate)-3,4-dichloro-1,5-dihydro-2H-pyrrol-2-one (Compound 18) 14
10) N-(3-aminophenyl)-3,4-dichloro-1,5-dihydro-2H-pyrrol-2-one (compound 19) 15
11) N-(4-aminophenyl)-3,4-dichloro-1,5-dihydro-2H-pyrrol-2-one (Compound 20) 16
12) 4-(3,4-dichloro-2-oxo-2,5-dihydro-1*H*-pyrrol-1-yl)butanoic acid (Compound 21) 17
13) 1-allyl-3,4-dichloro-1,5-dihydro-2*H*-pyrrol-2-one (Compound 22) 18
......18
14) 3,4-dichloro-1-phenyl-1,5-dihydro-2*H*-pyrrol-2-one (Compound 23) 19
15) 3,4-dibromo-1-phenyl-1,5-dihydro-2*H*-pyrrol-2-one (Compound 24) 20
16) 3,4-dibromo-1-(3-hydroxyphenyl)-1,5-dihydro-2*H*-pyrrol-2-one (Compound 25) 21
17) N-(3-carboxyphenyl)-3,4-dichloro-1,5-dihydro-2H-pyrrol-2-one (Compound 26) 22
18) N-(4-carboxyphenyl)-3,4-dichloro-1,5-dihydro-2H-pyrrol-2-one 23
(Compound 27) 23
19) N-(4'-tert-butylphenylcarbamate)-3,4-dibromo-1,5-dihydro-2H-pyrrol-2-one (Compound 28) 24
20) N-(4-aminophenyl)-3,4-dibromo-1,5-dihydro-2H-pyrrol-2-one (DHP phenyl amine-2) (Compound 29) 25

22) 1-(2-(1*H*-indol-3-yl)ethyl)-3,4-dichloro-1,5-dihydro-2*H*-pyrrol-2-one (Compound 31)....27
23) *N*-butyl-4-(3,4-dichloro-2-oxo-2,5-dihydro-1*H*-pyrrol-1-yl)benzamide (Compound 32)....28
24) *N*-hexyl-4-(3,4-dichloro-2-oxo-2,5-dihydro-1*H*-pyrrol-1-yl)benzamide (Compound 33)....29
_25)_ *N*-benzyl-4-(3,4-dichloro-2-oxo-2,5-dihydro-1*H*-pyrrol-1-yl)benzamide (Compound 34)....30
26) *N*-(2-(1*H*-indol-3-yl)ethyl)-4-(3,4-dichloro-2-oxo-2,5-dihydro-1*H*-pyrrol-1-yl)benzamide (Compound 35) 31
27) 4-(3,4-dichloro-2-oxo-2,5-dihydro-1*H*-pyrrol-1-yl)-*N*-(2-morpholinoethyl)benzamide (Compound 36)....32
28) 4-(3,4-dichloro-2-oxo-2,5-dihydro-1*H*-pyrrol-1-yl)-*N*-(3-morpholinopropyl)benzamide (Compound 37) 33
30) *N*-(4-(3,4-dichloro-2-oxo-2,5-dihydro-1*H*-pyrrol-1-yl)phenyl)butyramide (Compound 39)....35
31) *N*-(4-(3,4-dichloro-2-oxo-2,5-dihydro-1*H*-pyrrol-1-yl)phenyl)hexanamide (Compound 40)....36
32) N-(4-(3,4-dichloro-2-oxo-2,5-dihydro-1H-pyrrol-1-yl)phenyl)octanamide (Compound 41)....37
33) 2-(4-bromophenyl)-*N*-(4-(3,4-dichloro-2-oxo-2,5-dihydro-1*H*-pyrrol-1-yl)phenyl)acetamide (Compound 42)....38

Table S1. Growth inhibition (OD 600 reduction) at different concentrations.

	% reduction of bacterial growth		
**Compound**	**250 μM**	**125 μM**	**62.5 μM**
**9**	23.5 ± 1.0	11.8 ± 1.8	0
**10**	22.5 ± 0.5	9.1 ± 0.5	0
**11**	18.1 ± 3.4	12.0 ± 2.5	0
**12**	27.0 ± 5.7	9.2 ± 2.6	0
**13**	21.3 ± 3.6	6.1 ± 0.8	0
**14**	13.6 ± 3.3	11.6 ± 3.3	0
**15**	0	0	0
**16**	0	0	0
**19**	6.83 ± 0.6	0	0
**20**	4.10 ± 3.7	0	0
**21**	3.00 ± 3.26	0	0
**22**	25.2 ± 1.8	14.7 ± 0.89	0
**23**	21.2 ± 2.1	15.9 ± 0.008	9.06 ± 0.001
**24**	15.9 ± 3.0	12.5 ± 4.1	3.30 ± 0.005
**25**	14.7 ± 0.6	0	0
**26**	0	0	0
**27**	0	0	0
**29**	0	0	0
**30**	13.4 ± 1.7	5.9 ± 1.0	0
**31**	12.3 ± 6.5	4.7 ± 6.2	0
**32**	12.0 ± 4.5	7.5 ± 5	0
**33**	7.5 ± 2.5	5.2 ± 5.8	0
**34**	5.5 ± 3.9	0	0
**35**	6.0 ± 4.4	0	0
**36**	7.8 ± 0.5	3.9 ± 2.1	0
**37**	7.7 ± 5.4	7.0 ± 1.2	0
**38**	4.6 ± 3.4	0	0
**39**	0	0	0
**40**	0	0	0
**41**	0	0	0
**42**	0	0	0
**TP-5**	0		
**5**	86.5 ± 2.6	53.2 ± 4.5	44.2 ± 1.6

Table S2. Docking of lactams to the LasR receptor protein of *P. aeruginosa*.

Entry	GoldScore	[a]Pose No.	H-bond interactions	electrostatic interactions	[b]Hydrophobic and π interactions
**9**	48.12	1	Arg61, Trp60	Tyr64[d]	Ala105, Ala127, Leu36, Phe101, Trp88, Val76
**10**	53.36	1	Arg61	Tyr64[d]	Ala105, Ala127, Leu36, Leu110, Trp88, Val76
**11**	53.33	1	Arg61	TYR64 [d]	Ala70, Ala50, Ile52, Val76
**12**	48.00	3	Trp60, Tyr56	—	Leu36, Phe101, Trp88, Tyr64
**13**	53.6	1	Arg61, Trp60	Leu110[c]	Leu36, Trp88, Tyr56, Tyr93,
**14**	52.26	1	Leu110, Trp60,	—	Ala105, Ala127, Leu36, Phe101, Trp88, Val76,
**15**	58.90	6	Arg61, TRP60, TYR93	Leu110[c], Trp88, Tyr64[e]	Ile92, Leu36, Tyr56
**16**	58.33	1	Arg61, Leu110	Asp73[d], Trp88, Tyr64[e]	Leu36, Tyr56, Val76
**19**	54.25	1	Arg61	Asp73[d], Tyr64[e]	Ala127, Leu36, Val76
**20**	52.67	1	Arg61	Asp73[d], Tyr64[e]	Ala127, Leu36, Val76
**21**	50.47	15	Arg61	Asp73[d], Tyr64[e]	Ala127, Leu36, Val76
**22**	43.31	1	Trp60	—	Ala127, Trp88, Val76
**23**	51.06	4	Trp60	—	Ala105, Ala127, Leu36, Leu110, Phe101, Trp88, Tyr64
**24**	52.39	1	Arg61	Asp73[d], Tyr64[e]	Ala127, Leu36, Val76
**25**	55.22	12	Trp60, Arg61, ,Tyr93	Leu110[c], Tyr64[e]	Trp88, Tyr56, Leu36
**26**	57.87	7	Arg61, Trp60	Trp88[e]	Ala127, Leu36, Tyr93, Val76
**27**	59.58	1	Arg61, Leu110, Tyr93	Asp73[d]	Ala127, Leu36, Tyr56
29	*52.32*	1	Arg61, Trp60	—	Ala50, , Ala70, Ala105, Leu110, Trp88, Tyr47
30	62.25	1	Arg61, THR 115	Asp65[c], Asp73[d]	Ala50, , Ala70, Ala105, Tyr47, Trp88, Leu110,
**31**	60.95	7	Arg61, Leu110	Tyr64[e]	Ala105, Ala127, Leu36, Trp88,
**32**	65.87	1	Trp60	—	Leu36, Leu40, Leu125, Trp88, Tyr64, , Tyr47
**33**	73.79	1	Arg61	Leu110[c]	Leu36, Leu40, Leu125, Tyr64, Tyr93, Ile92, Tyr47
**34**	74.59	1	Trp60	—	Ala50, Ala127, Leu36, Leu40 Trp88, Tyr64,

**35**	79.83	1	Arg61	Leu110[c]	Ala50, Ala70, Leu36, Leu40, Ile92, Tyr47, Tyr56, Tyr93, Val76
**36**	66.85	1	Thr115, Arg61	Leu110[c], Thr75[f]	Ala50, Ala70, Ala105, Leu36, Tyr47, Tyr56, Tyr64, Val76
**37**	74.03	1	Arg61, Trp60, Tyr93	Leu110[c]	Ala50, Ala127, Leu40, Leu36, Tyr64, Val76
38	64.20	1	Arg61	Leu110[c]	Ala50, Leu40, , Leu36, Leu125, Ile92, Tyr47, Trp88, Tyr93, Tyr56
**39**	62.76	1	Trp60	Asp73[d]	Ala127, Leu36, Leu40, Leu125, Trp88, Tyr47, Tyr64
**40***	68.74	1	Trp60	—	Ala127, Cys79, Leu36, Leu125, Trp88, Tyr47, Tyr64, , Val76
**41***	73.63	1	Tyr64	Arg61[f], Leu110[c]	Ile92, Leu36, Leu125, Leu40, Tyr47, Tyr56, Tyr93, Val76
**42**	79.36	5	Trp60, Tyr64	Asp73[d], Leu110[c], Leu125[c]	Ala50, Leu36, Leu140, Tyr56, Tyr93,
**5**	43.27	1	Arg61, Trp60	Asp73[d], Tyr64[e]	Ala105, Leu36, leu110, Phe101, Trp60
**3**[g]	68.32	2	Asp37, Ser129, Trp60, Tyr56	—	Ala127, Cys79, Leu125
**3**[h]	—		Tyr56, Trp60, Asp73	—	Trp88

[a]The highest-ranked pose of the largest cluster was selected for analysis.
[b]Hydrophobic interactions include π-π stacked and T-shaped, alkyl and π-alkyl and π-sigma; other interactions include [c]halogen and [d]π-anion and [e] π-lone pair;[f] unfavorable interaction; [g] Docked OdDHL, [h] OdDHL crystal structure.

Spectra Data ^{1}H NMR and ^{13}C NMR Spectra

1) 1-butyl-3,4-dichloro-1,5-dihydro-2*H*-pyrrol-2-one (Compound 9)

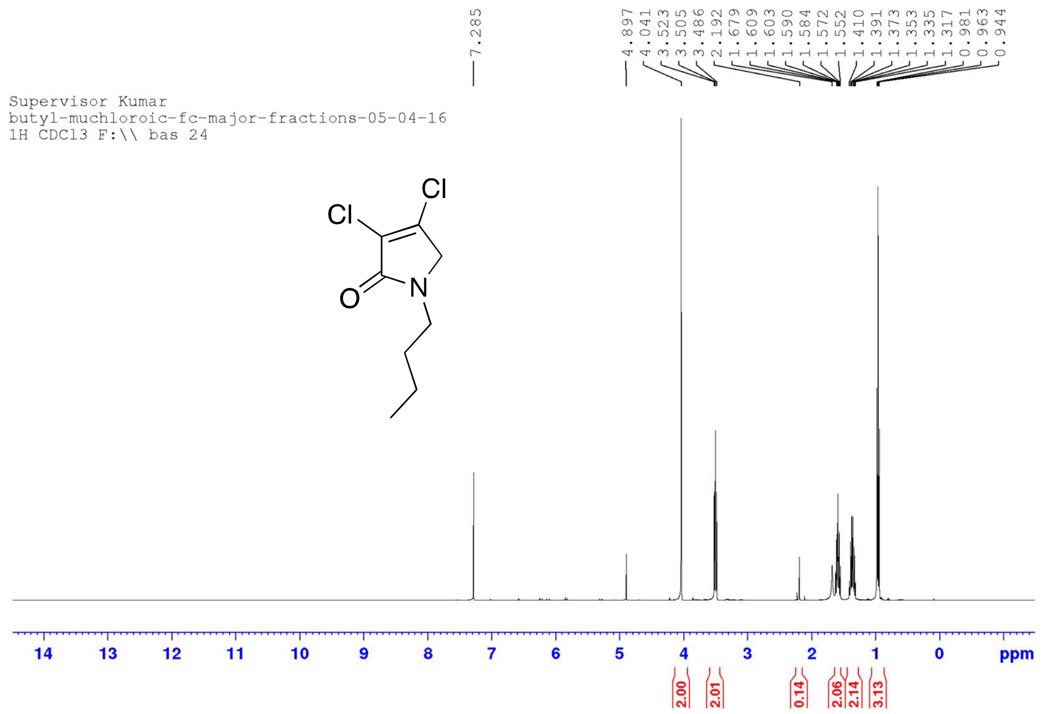

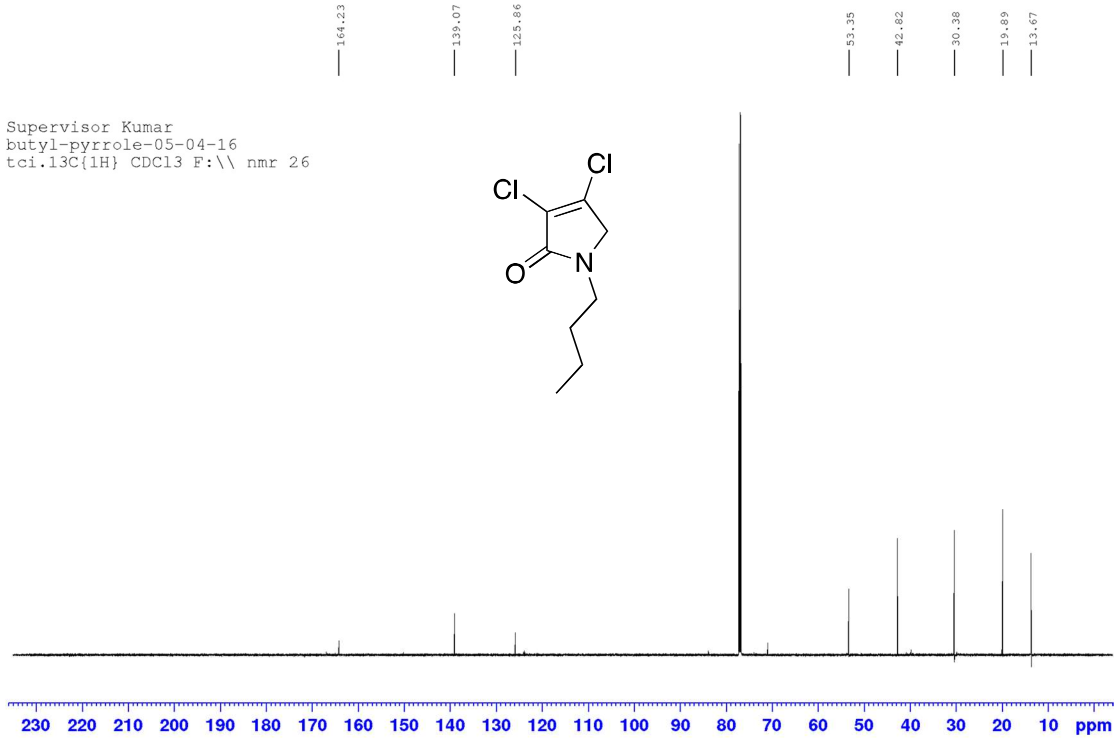

2) 3,4-dichloro-1-hexyl-1,5-dihydro-2*H*-pyrrol-2-one (Compound 10)

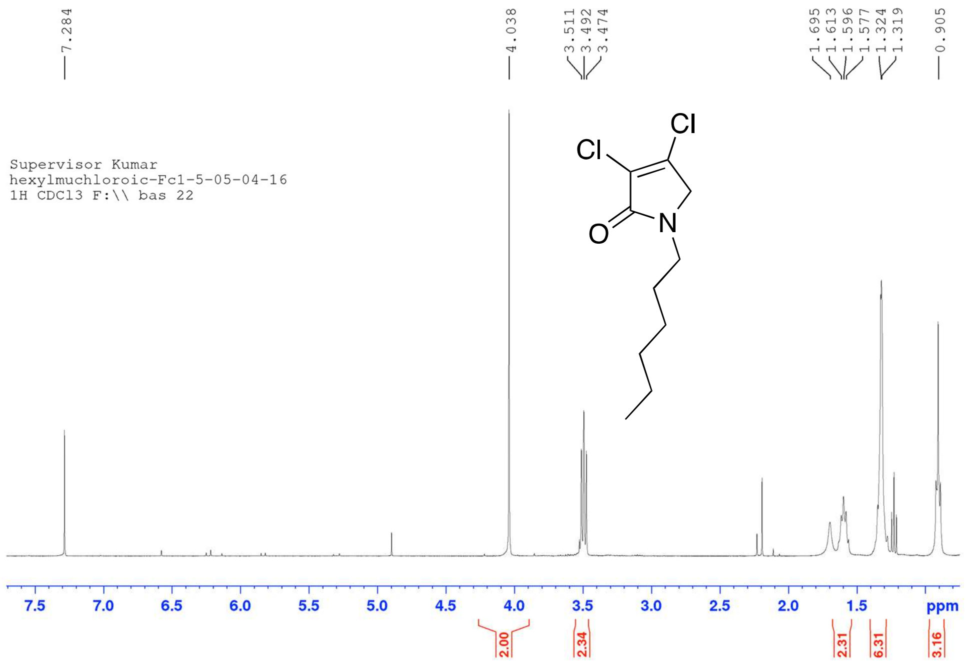

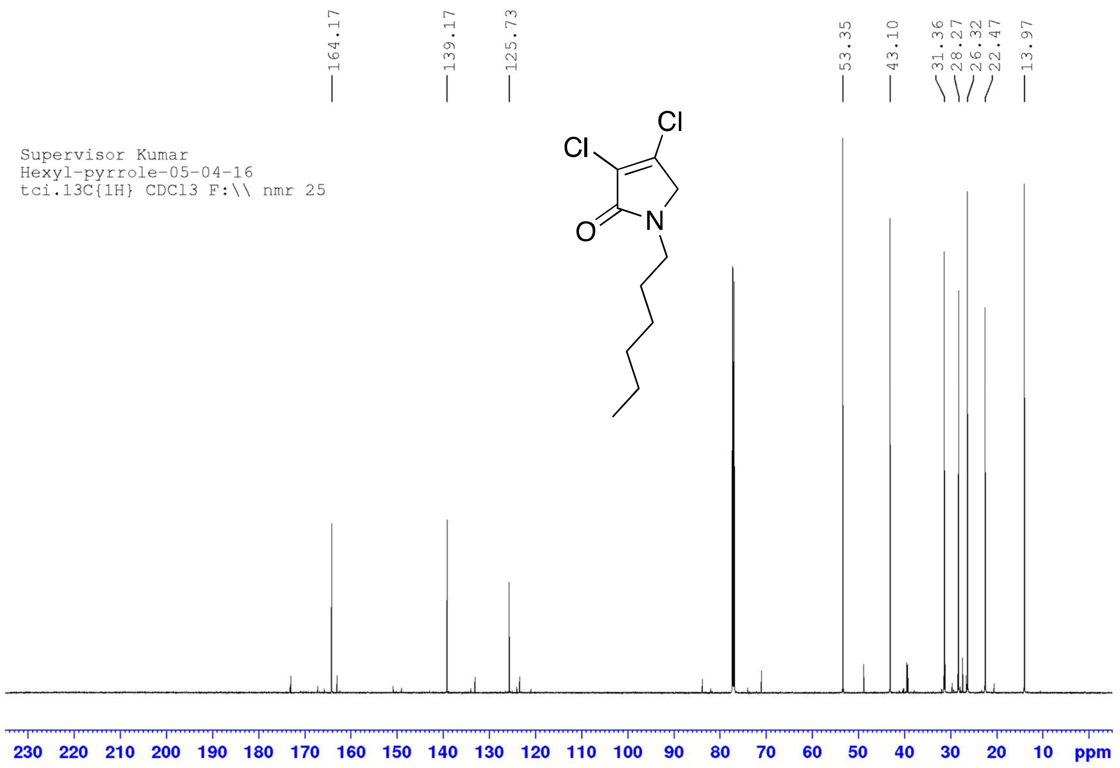

3) 3,4-dichloro-1-(2-hydroxyphenyl)-1,5-dihydro-2H-pyrrol-2-one (Compound 12)

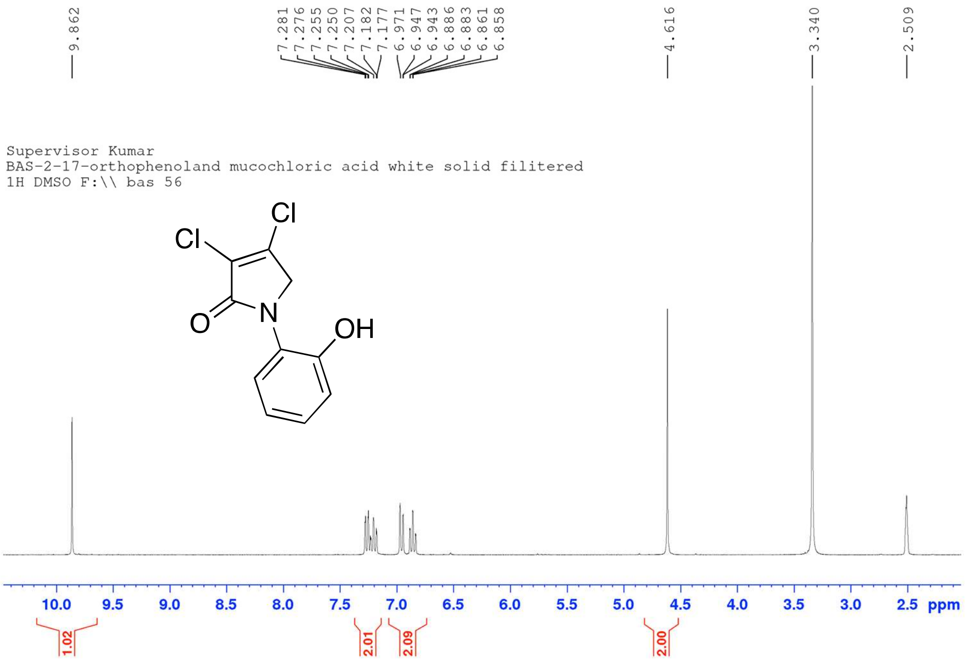

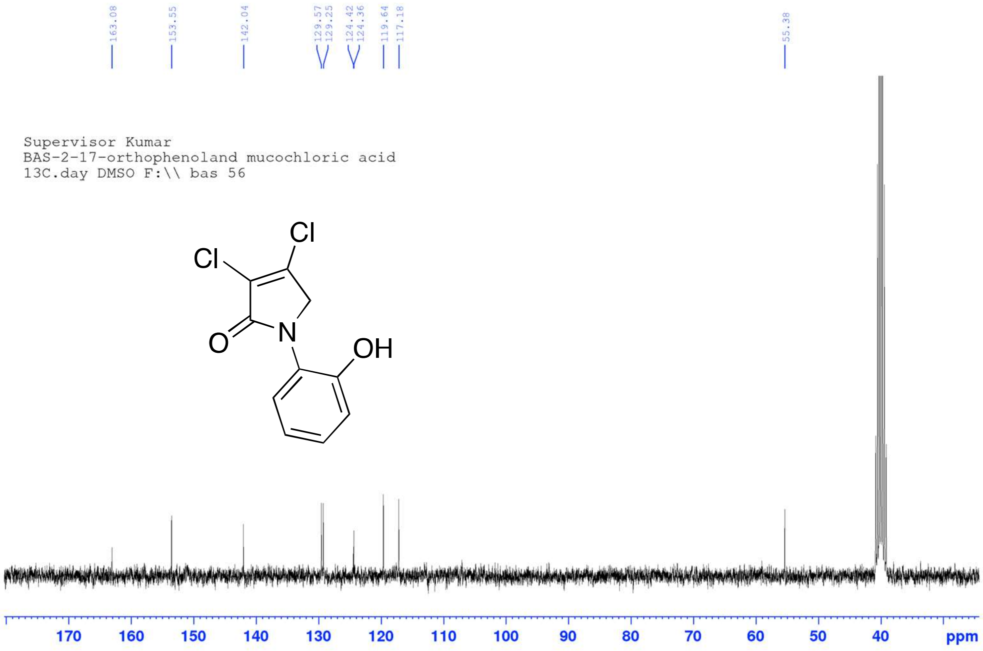

4) 3,4-dichloro-1-(3-hydroxyphenyl)-1,5-dihydro-2*H*-pyrrol-2-one (Compound 13)

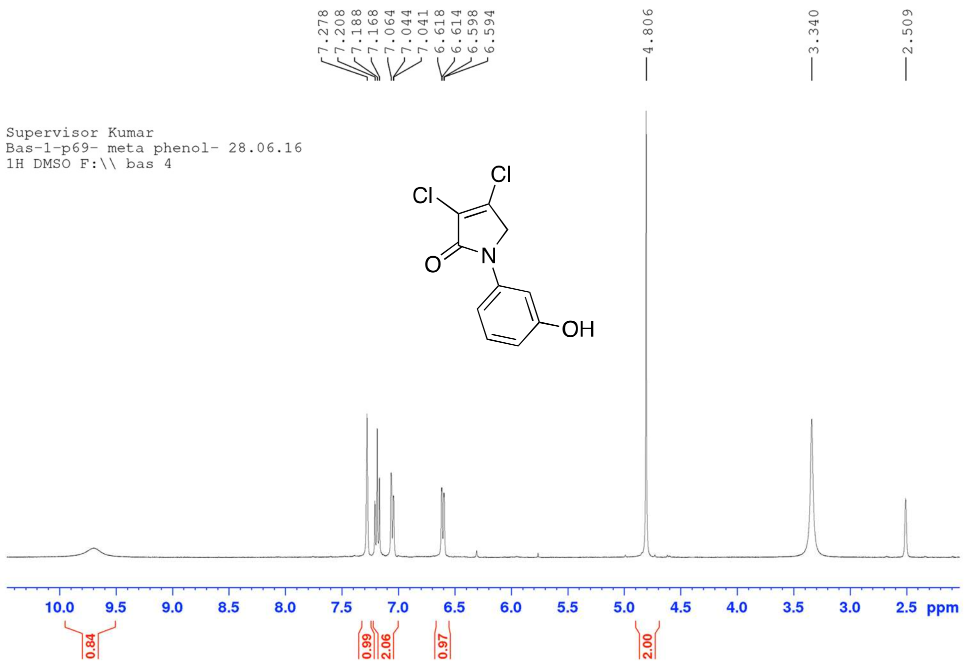

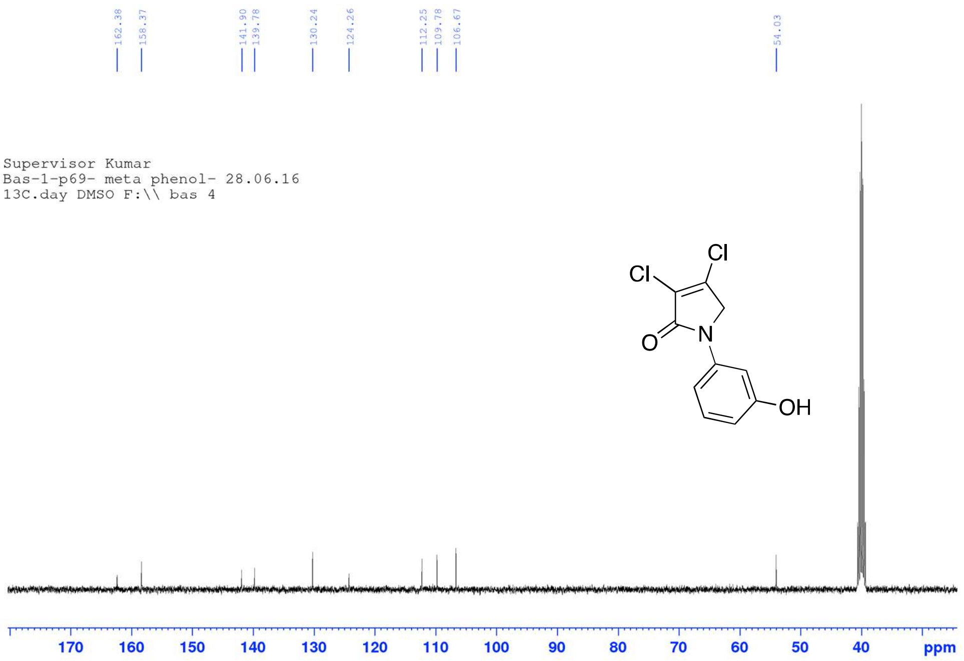

5) 3,4-dichloro-1-(4-hydroxyphenyl)-1,5-dihydro-2*H*-pyrrol-2-one (Compound 14)

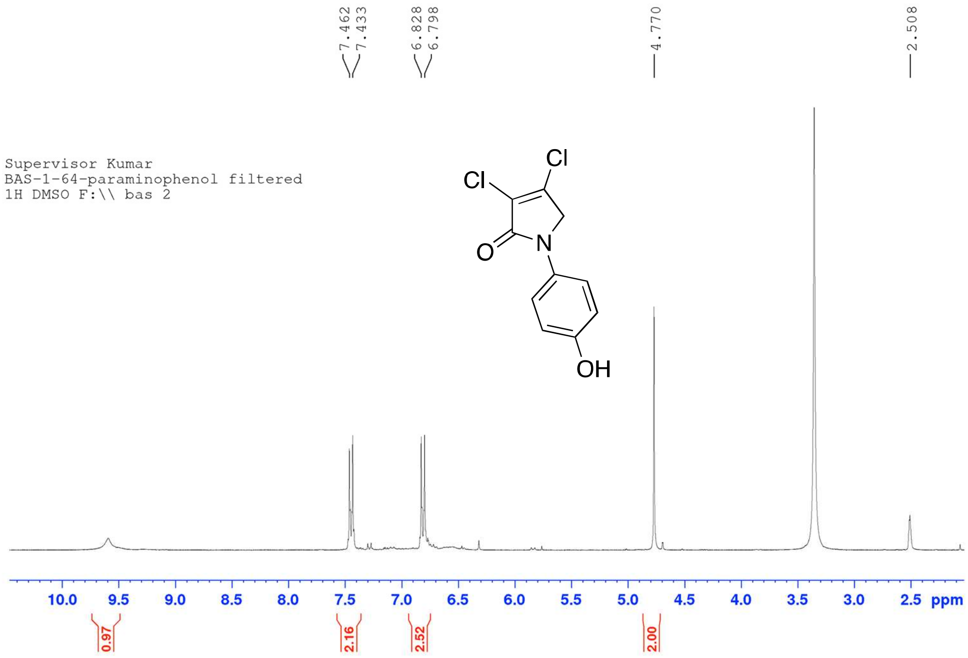

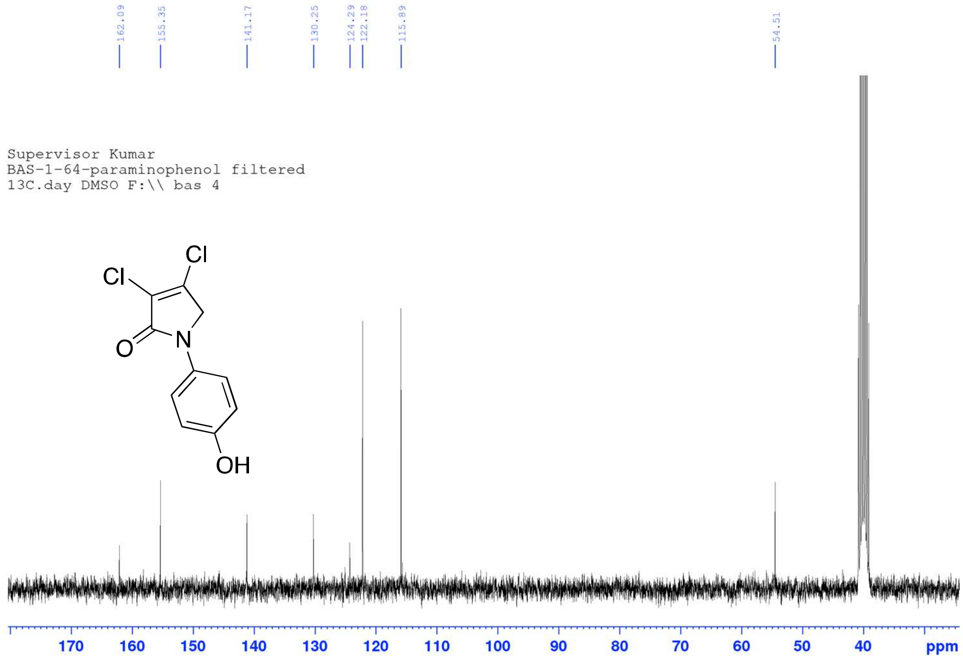

6) **N-(3-carboxyphenyl)-3,4-dibromo-1,5-dihydro-2H-pyrrol-2-one (DHP phenyl acid-2) (Compound 15)**

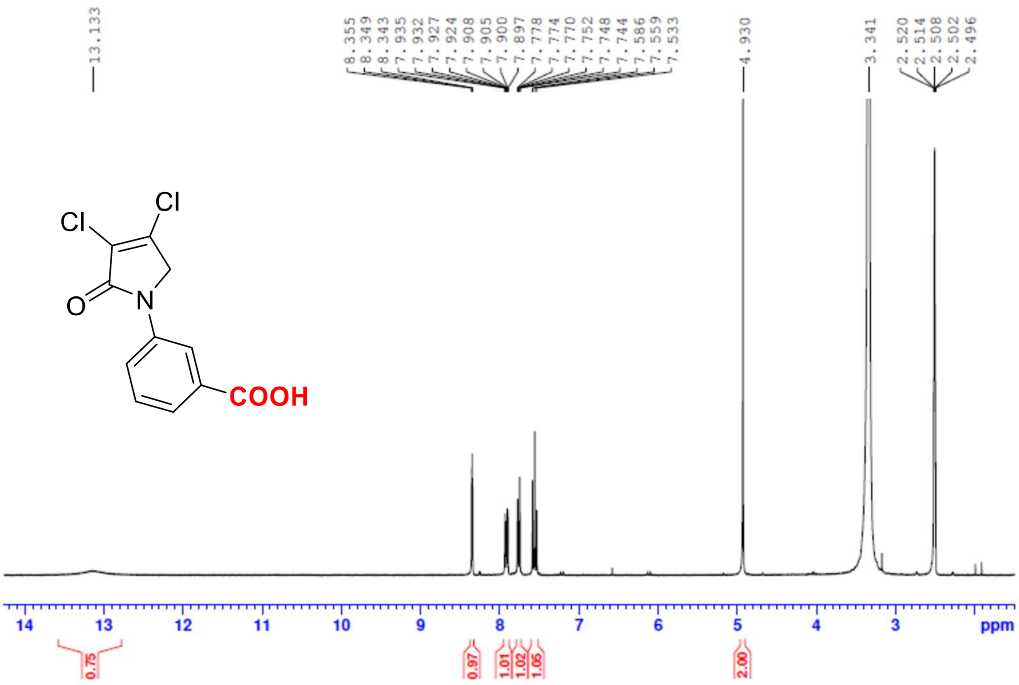

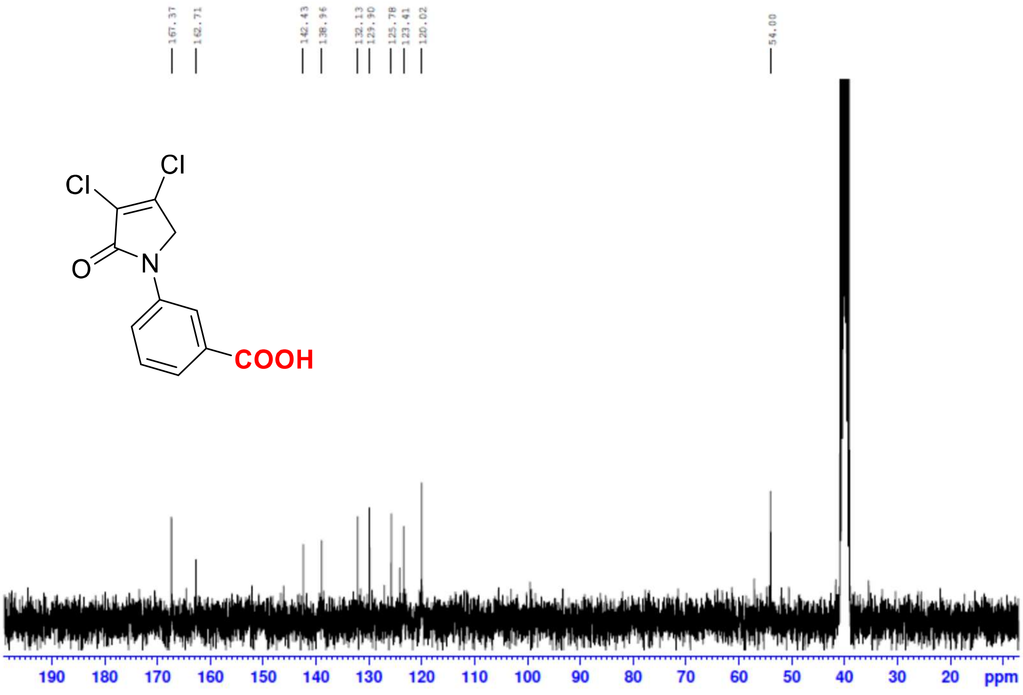

7) N-(4-carboxyphenyl)-3,4-dichloro-1,5-dihydro-2H-pyrrol-2-one (DHP phenyl acid-1) (Compound 16)

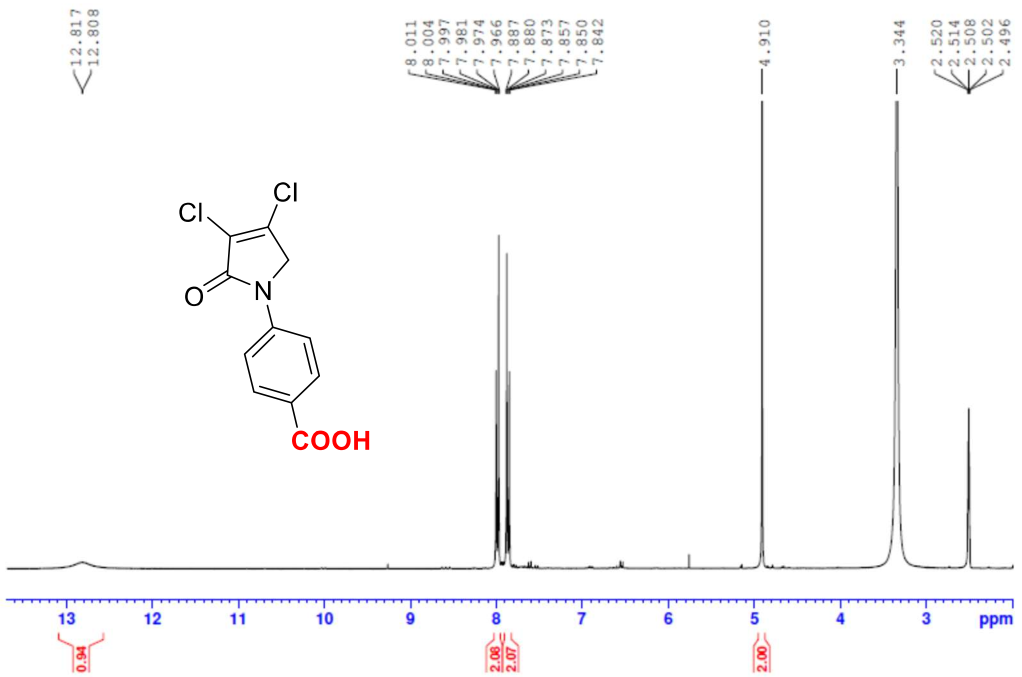

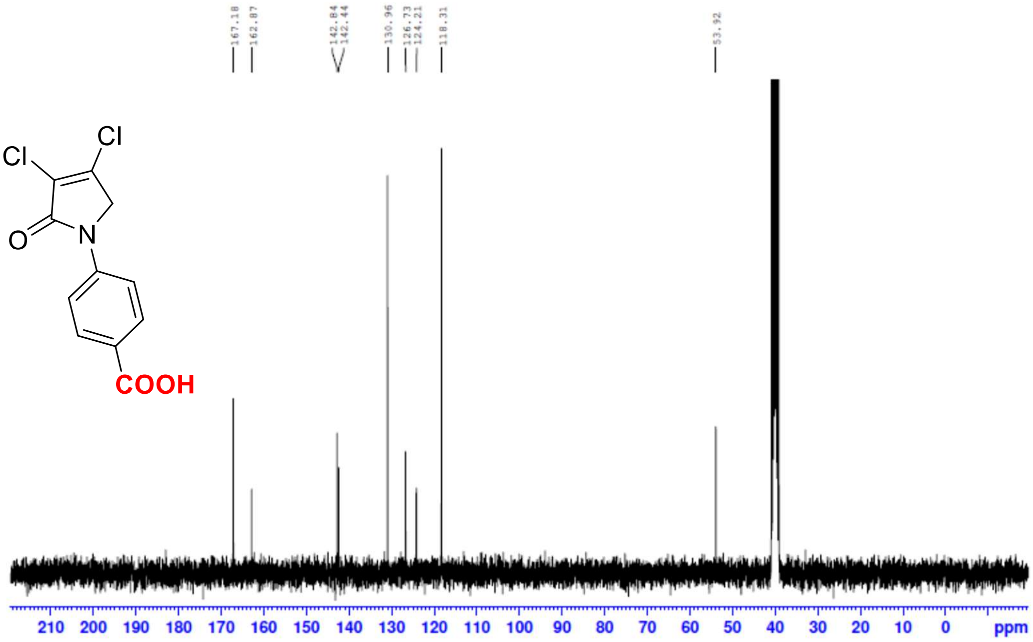

8) N-(3-tert-butylphenylcarbamate)-3,4-dichloro-1,5-dihydro-2H-pyrrol-2-one (Compound 17)

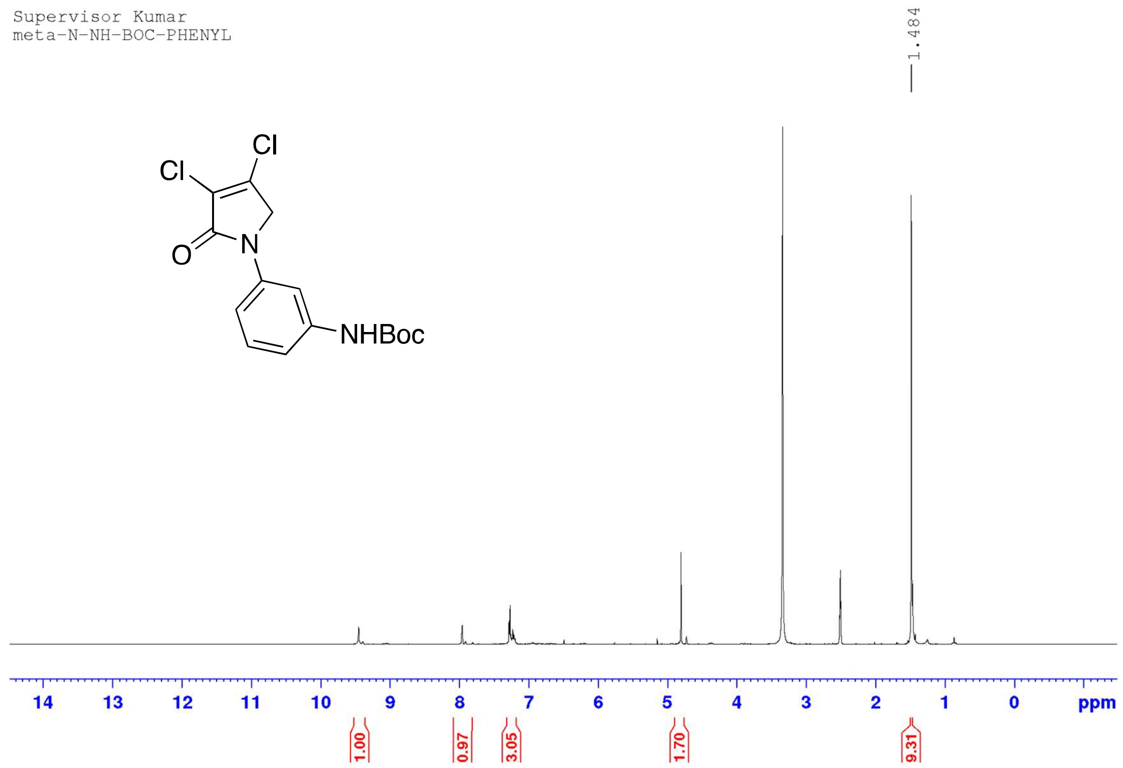

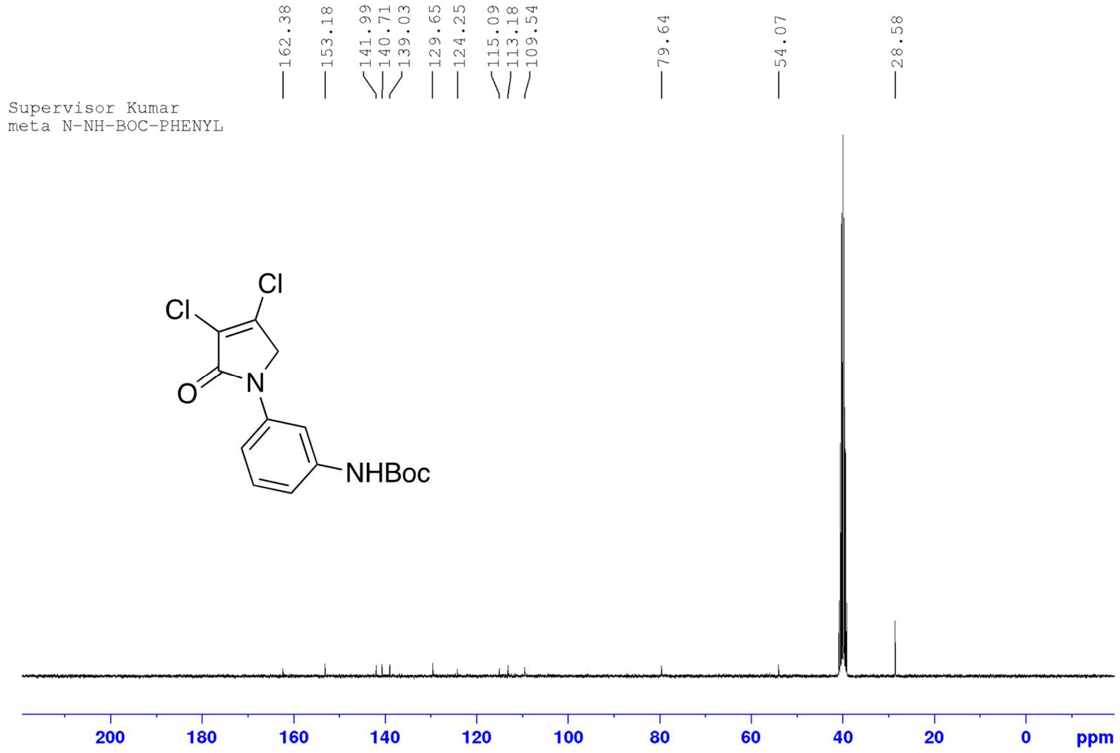

9) N-(4′-tert-butylphenylcarbamate)-3,4-dichloro-1,5-dihydro-2H-pyrrol-2-one (Compound 18)

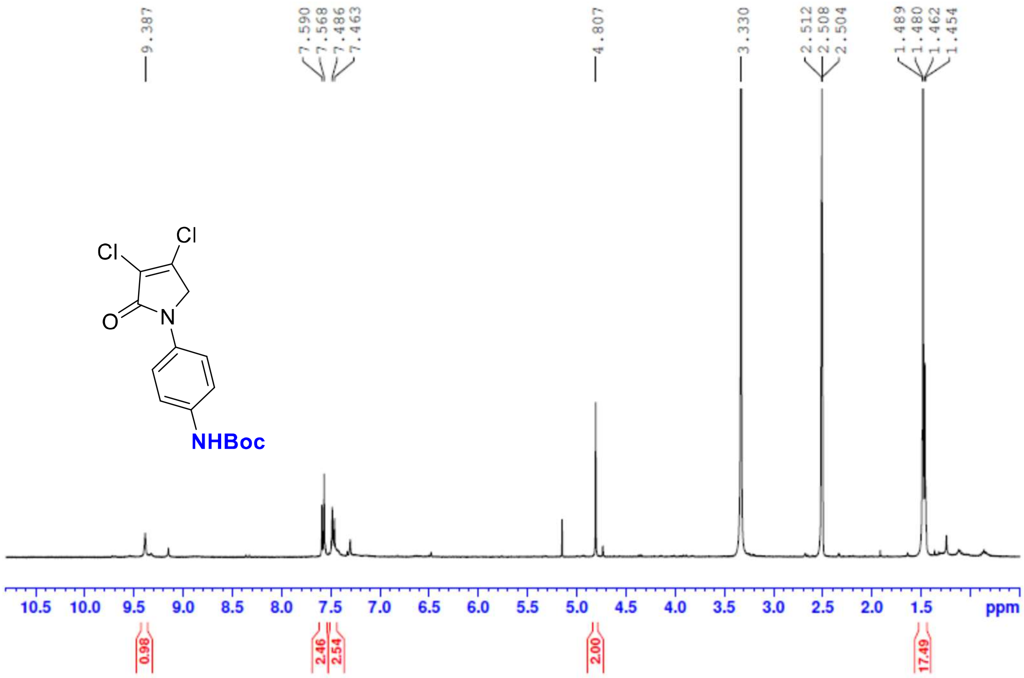

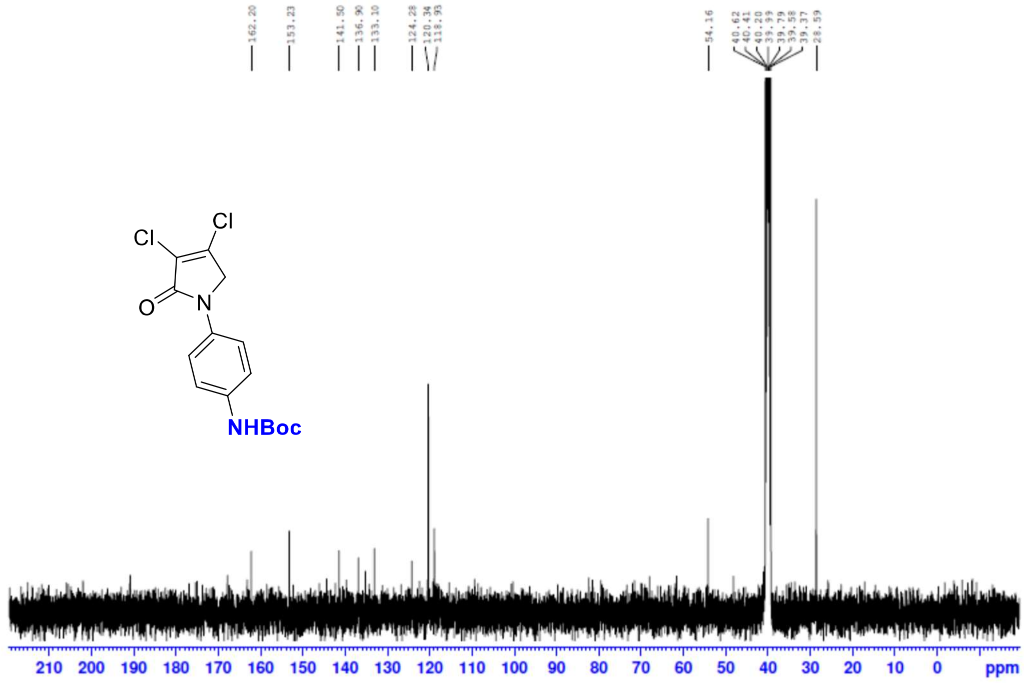

10) N-(3-aminophenyl)-3,4-dichloro-1,5-dihydro-2H-pyrrol-2-one (compound 19)

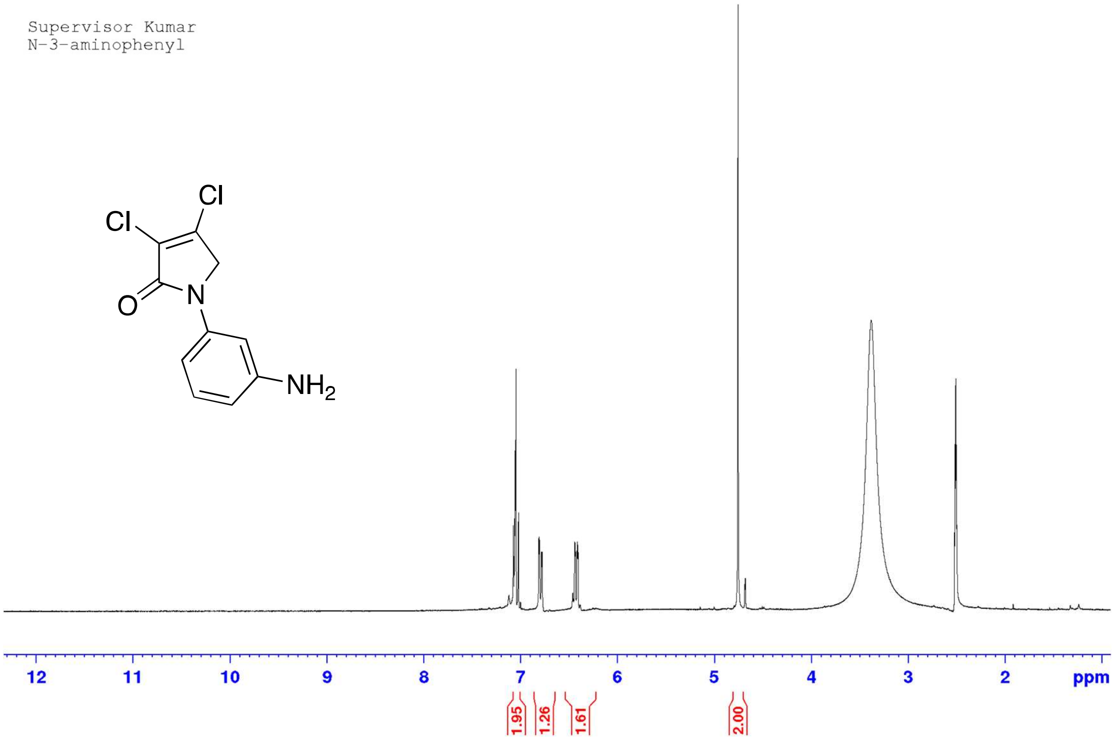

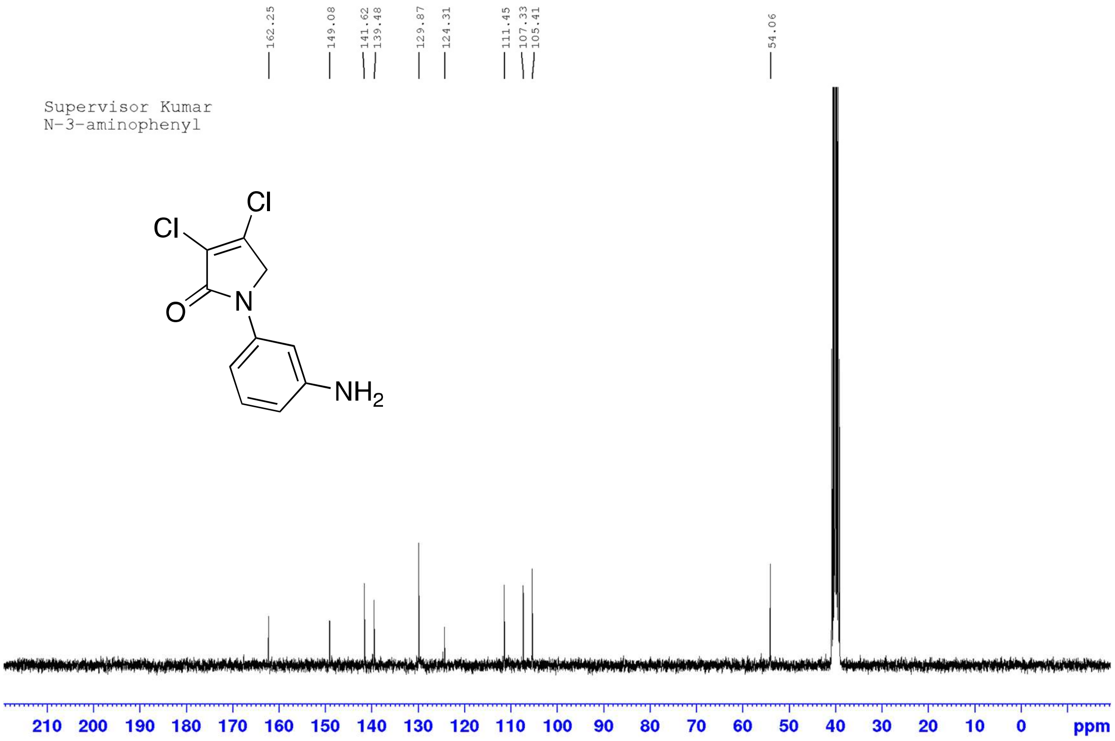

11) N-(4-aminophenyl)-3,4-dichloro-1,5-dihydro-2H-pyrrol-2-one (Compound 20)

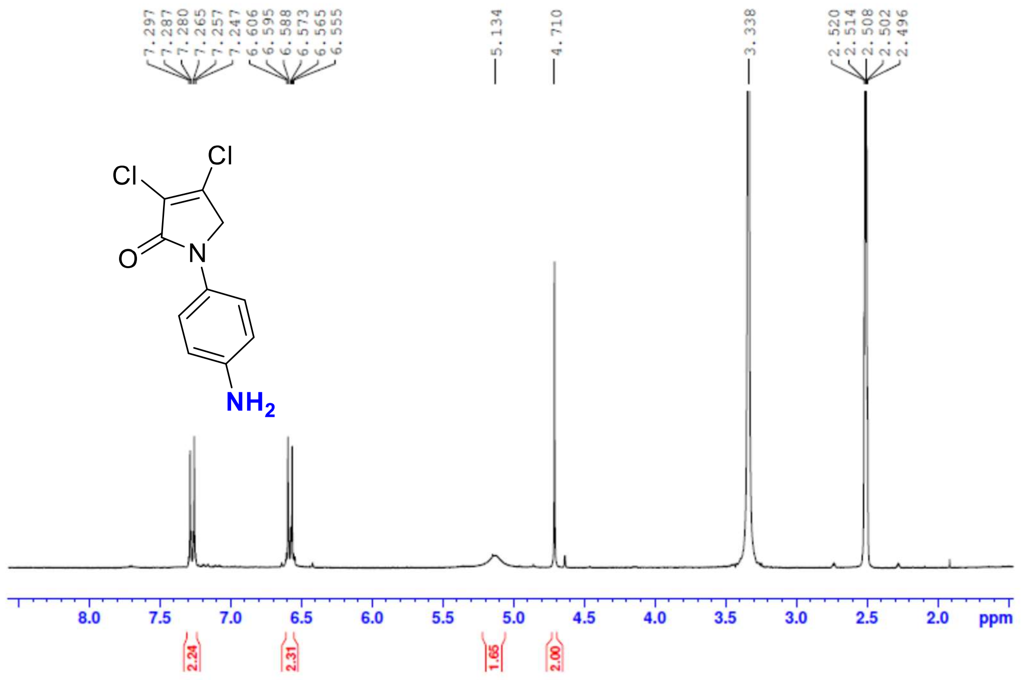

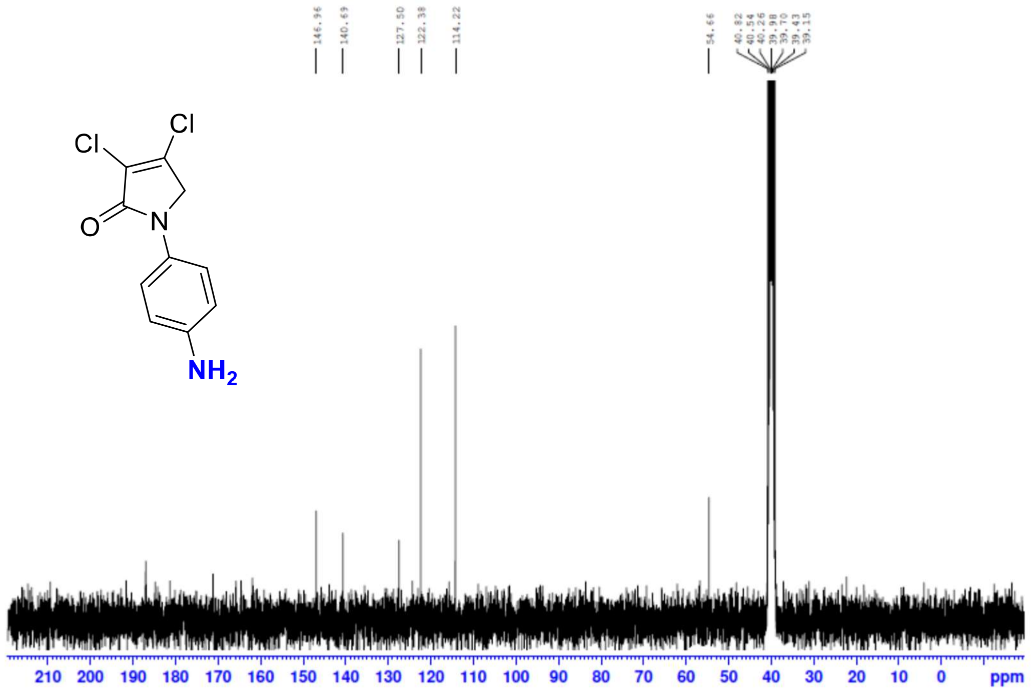

12) 4-(3,4-dichloro-2-oxo-2,5-dihydro-1*H*-pyrrol-1-yl)butanoic acid (Compound 21)

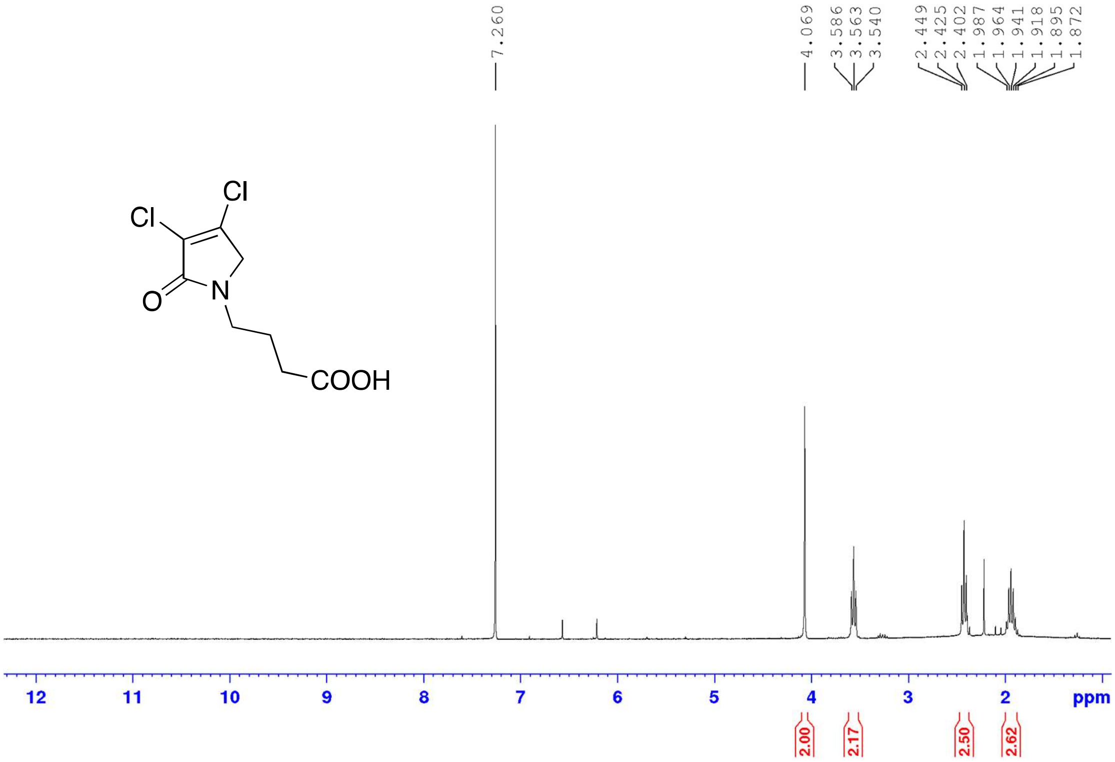

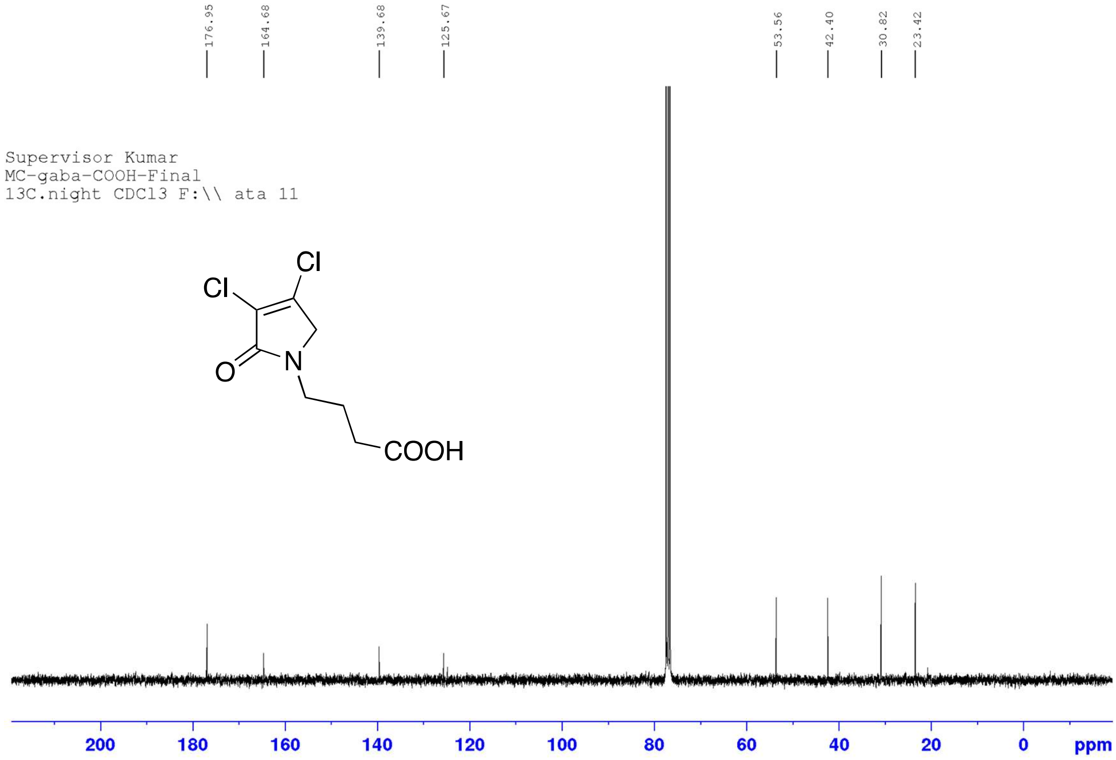

13) 1-allyl-3,4-dichloro-1,5-dihydro-2*H*-pyrrol-2-one (Compound 22)

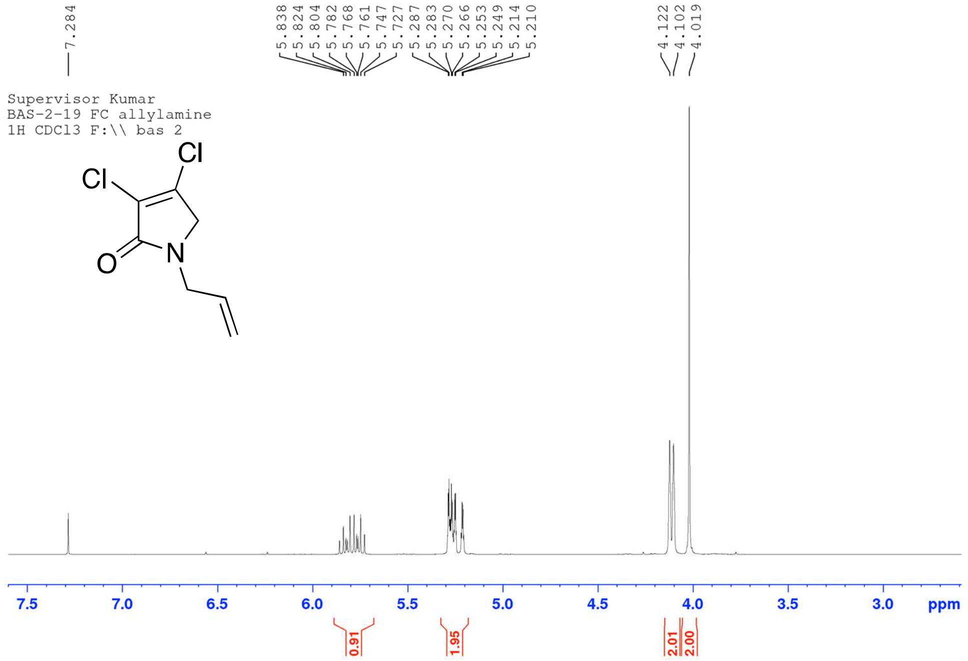

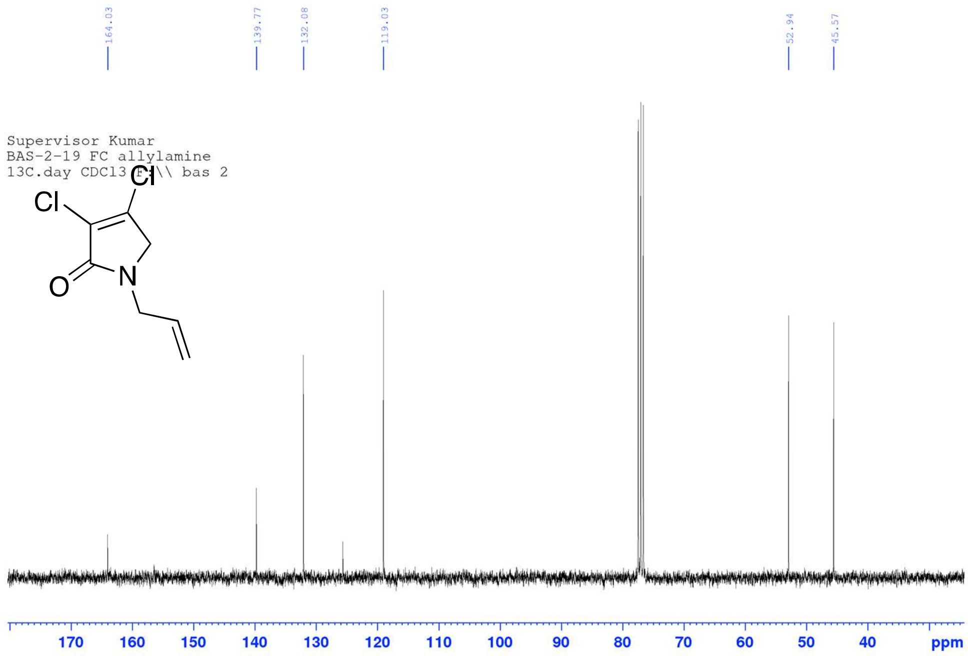

14) 3,4-dichloro-1-phenyl-1,5-dihydro-2*H*-pyrrol-2-one (Compound 23)

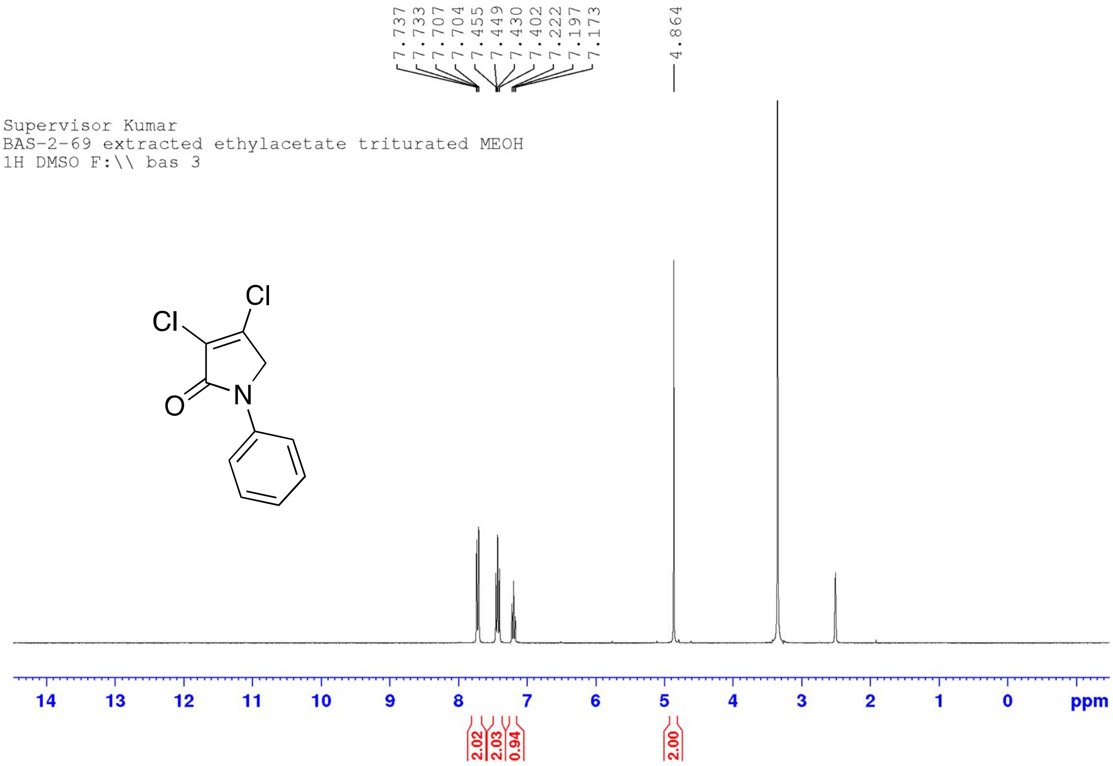

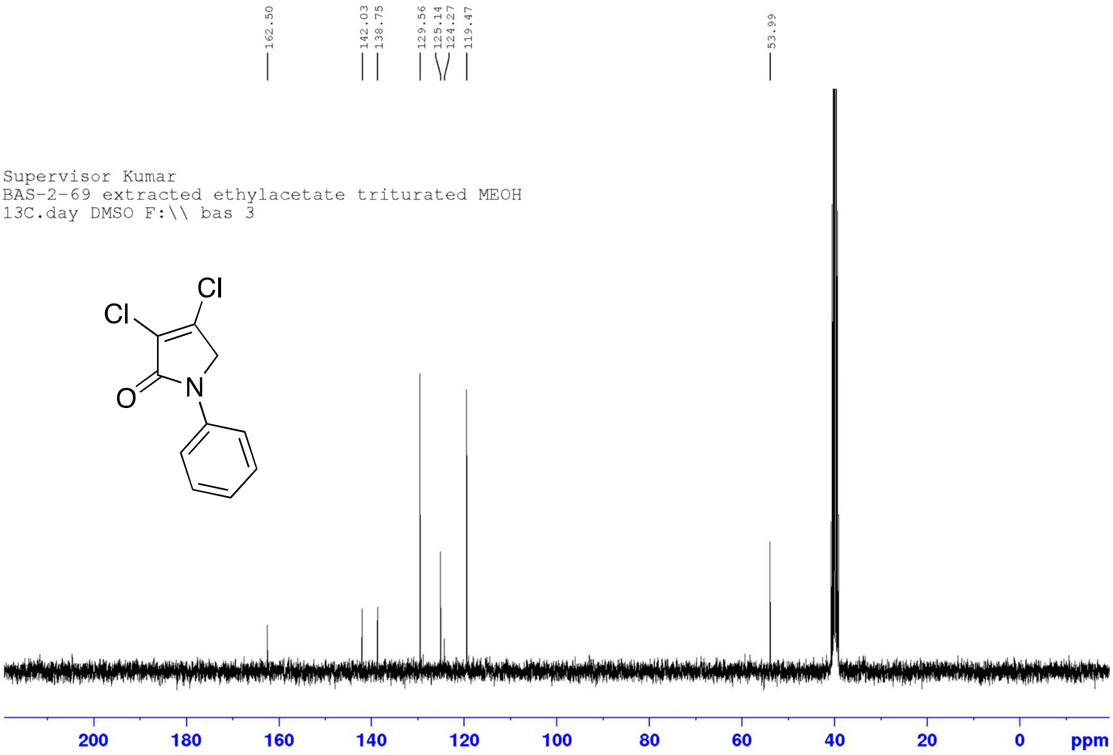

15) 3,4-dibromo-1-phenyl-1,5-dihydro-2*H*-pyrrol-2-one (Compound 24)

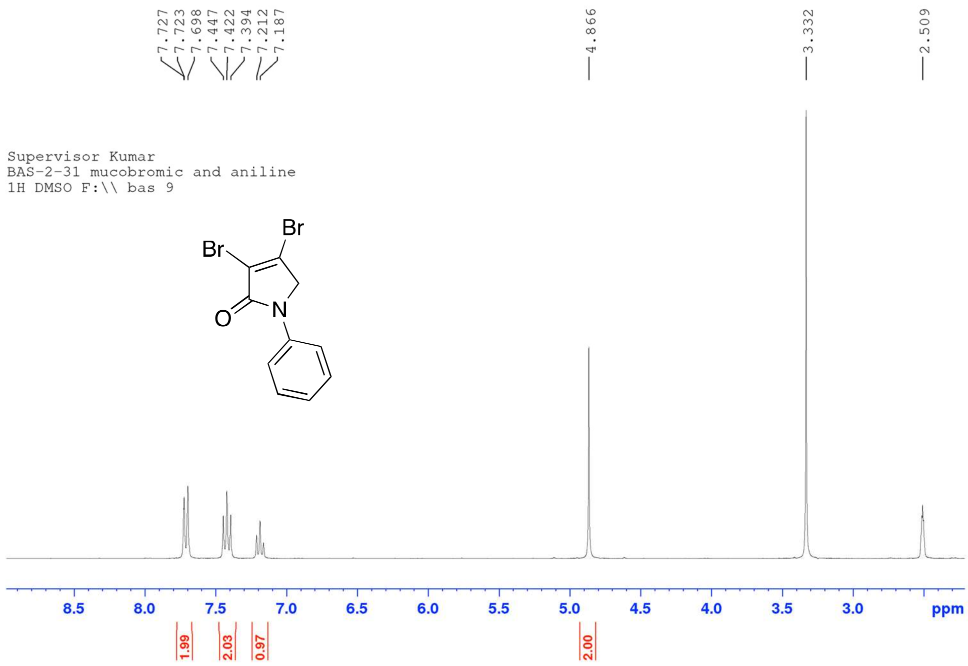

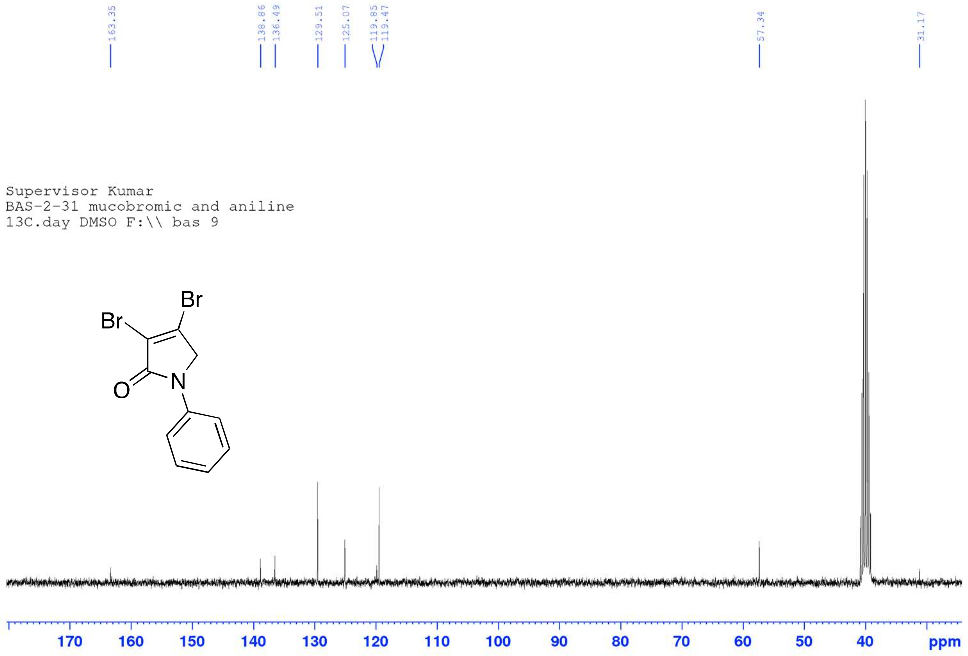

16) 3,4-dibromo-1-(3-hydroxyphenyl)-1,5-dihydro-2*H*-pyrrol-2-one (Compound 25)

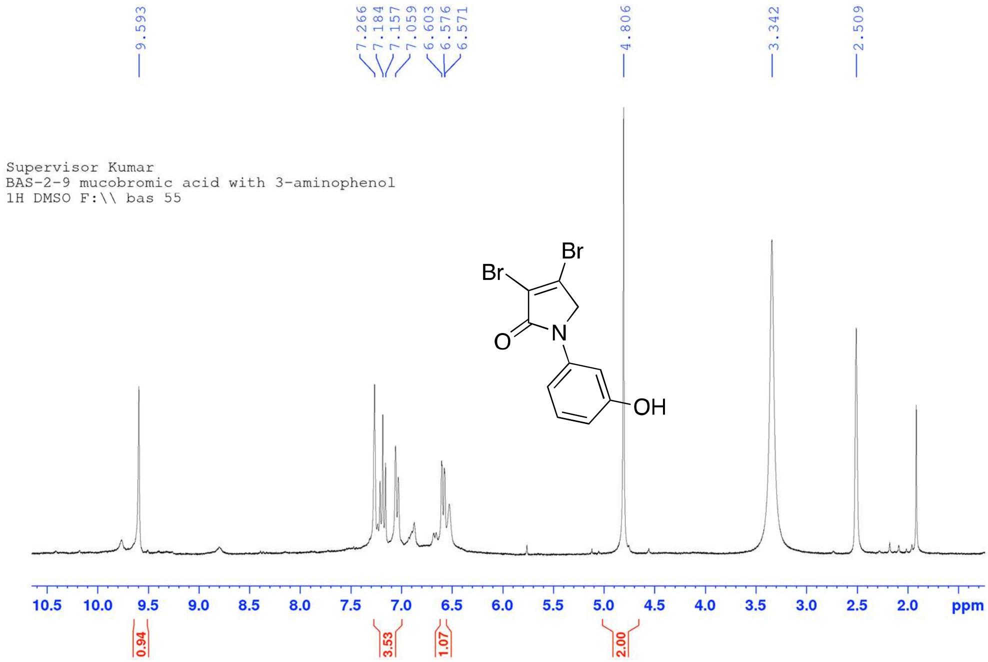

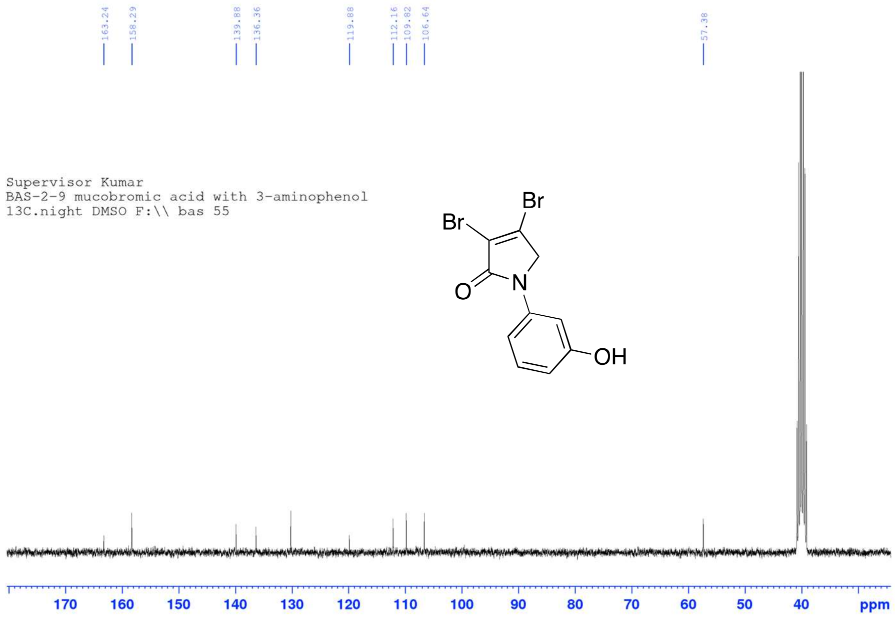

17) N-(3-carboxyphenyl)-3,4-dichloro-1,5-dihydro-2H-pyrrol-2-one (Compound 26)

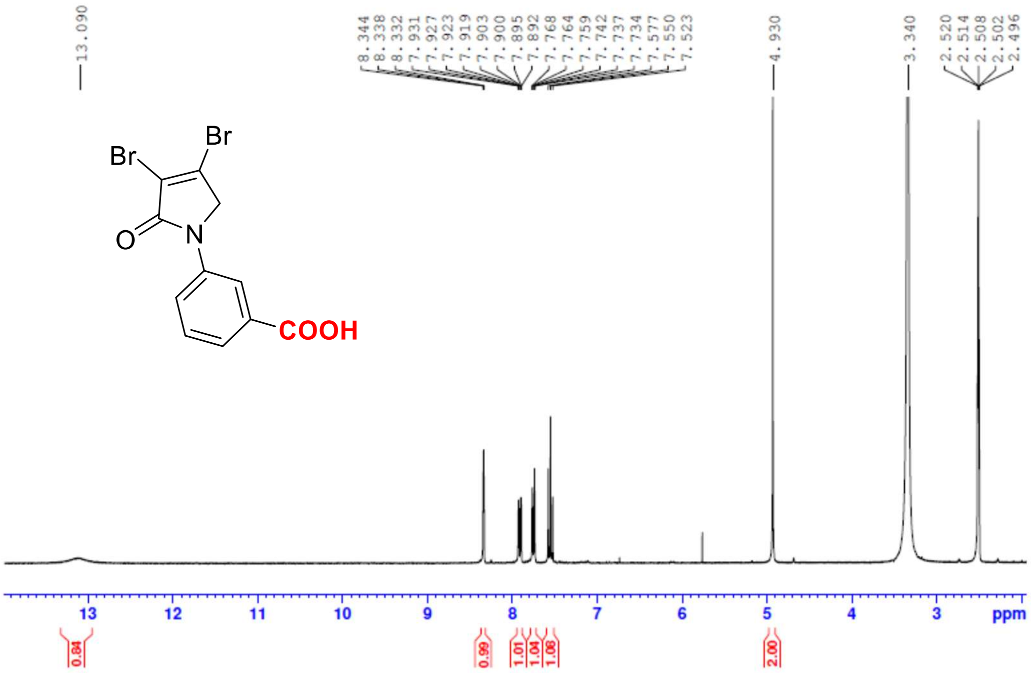

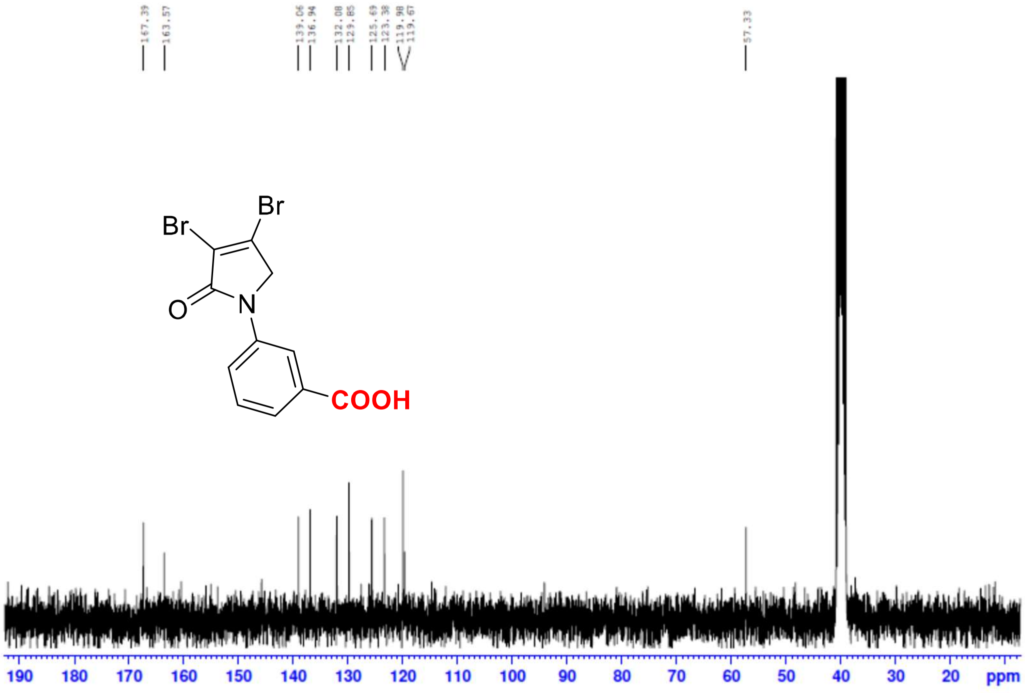

18) N-(4-carboxyphenyl)-3,4-dichloro-1,5-dihydro-2H-pyrrol-2-one (Compound 27)

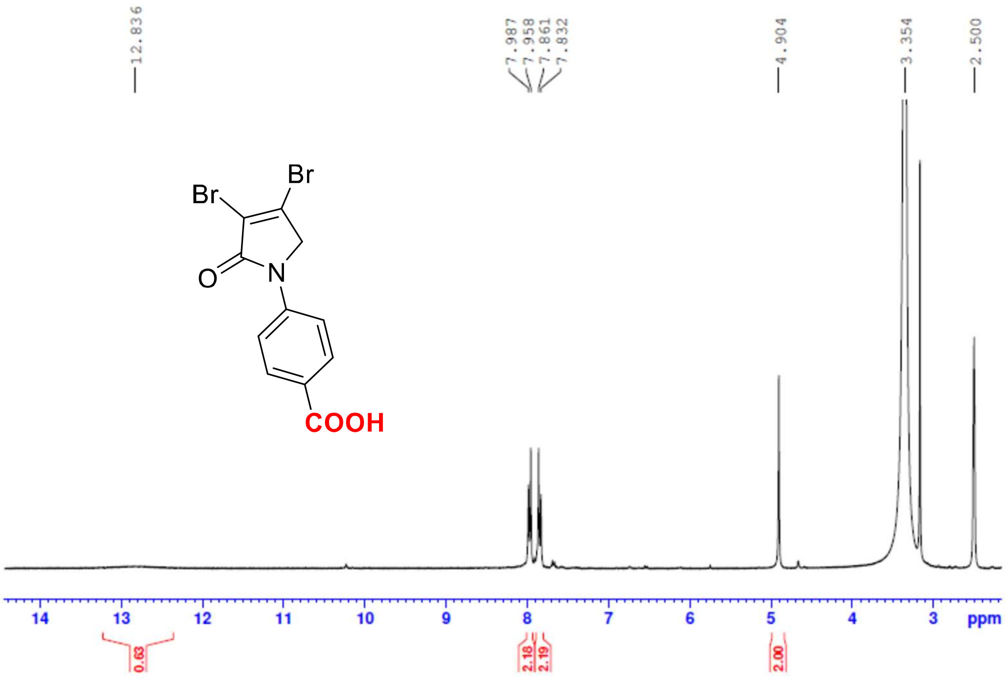

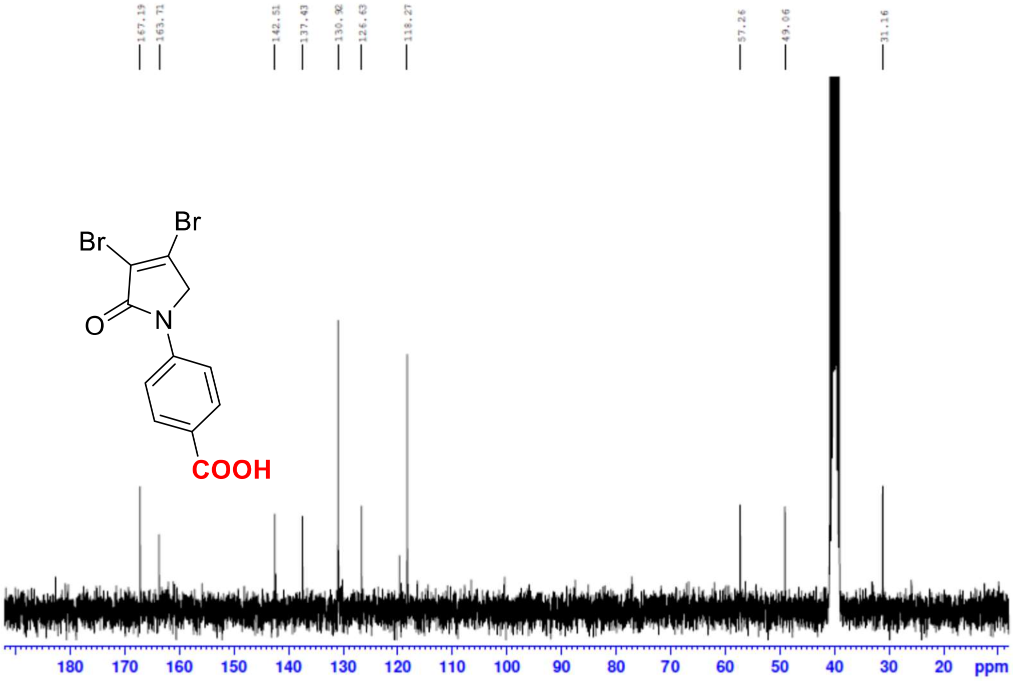

19) N-(4′-tert-butylphenylcarbamate)-3,4-dibromo-1,5-dihydro-2H-pyrrol-2-one (Compound 28)

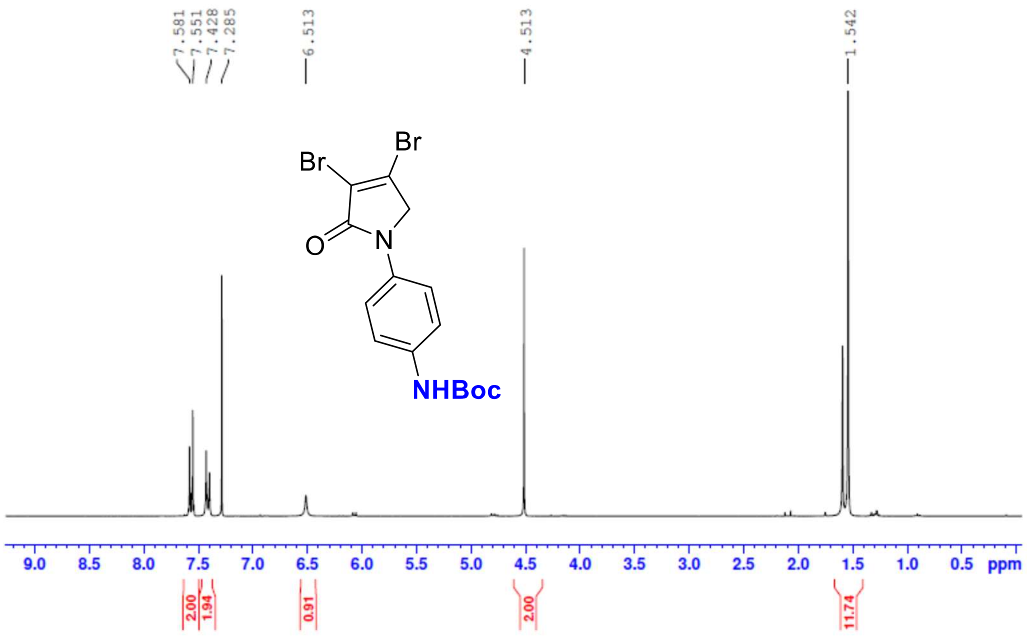

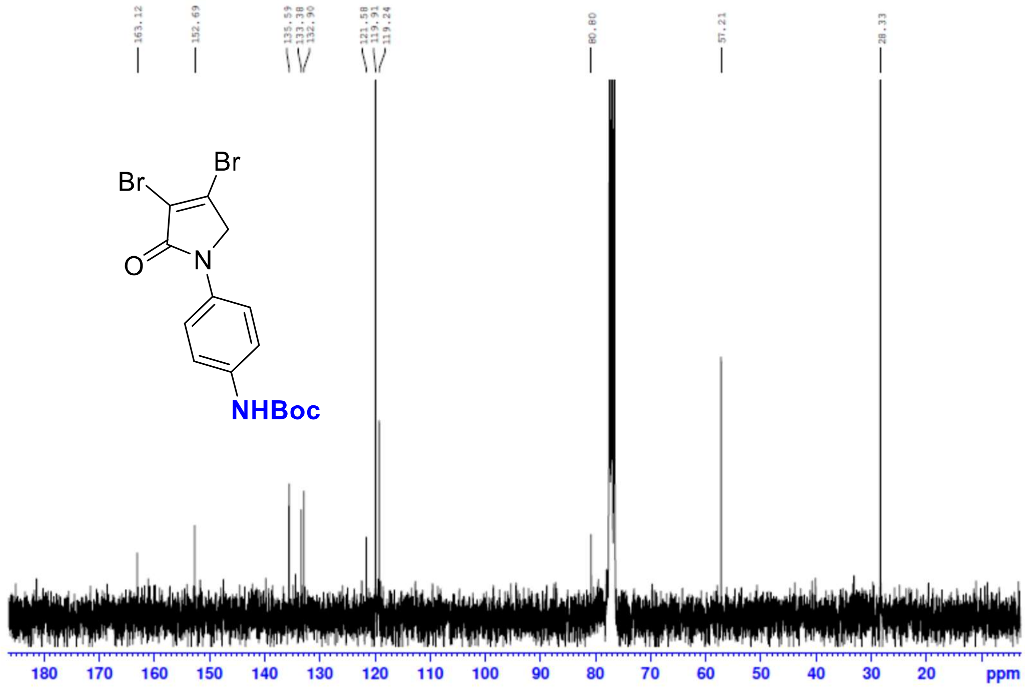

20) N-(4-aminophenyl)-3,4-dibromo-1,5-dihydro-2H-pyrrol-2-one (DHP phenyl amine-2) (Compound 29)

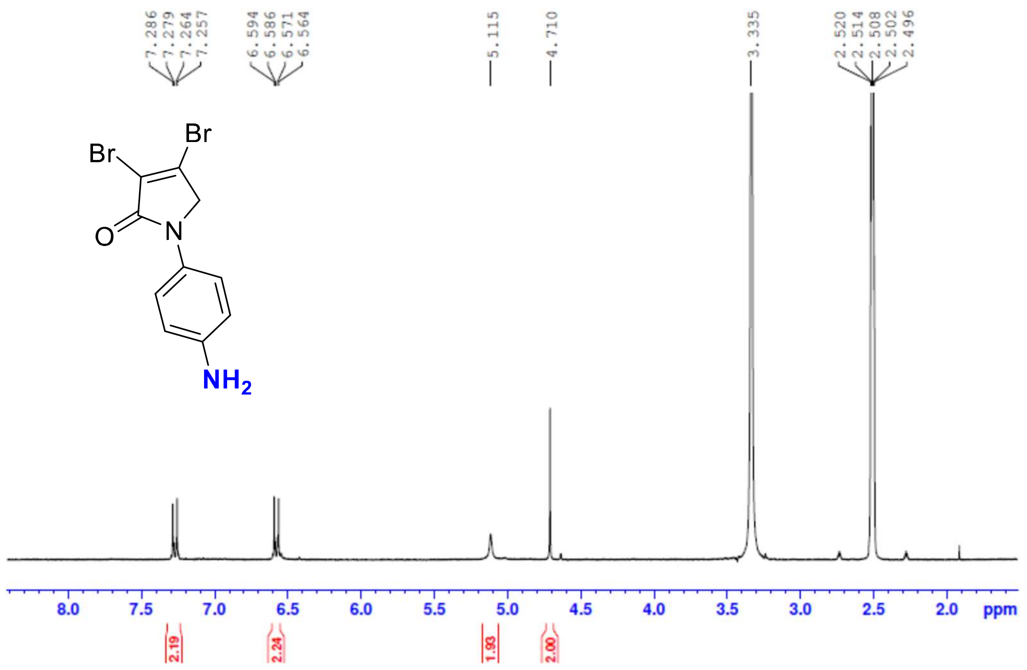

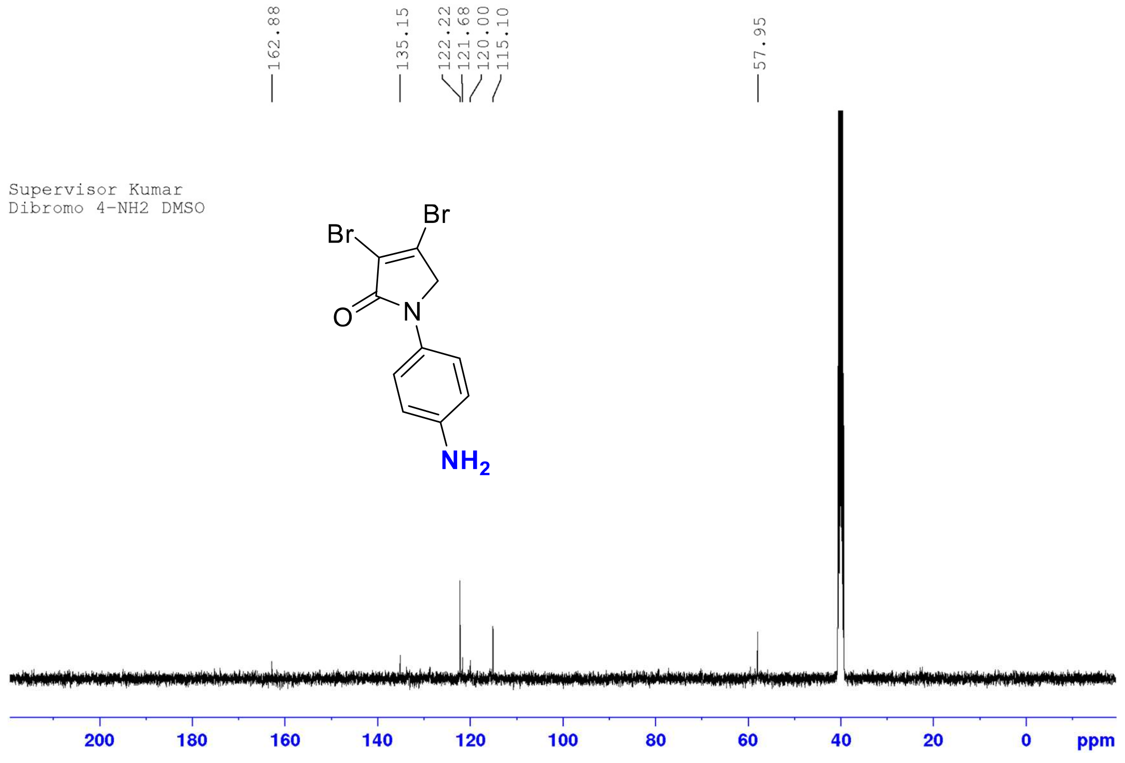

21) 3,4-dichloro-1-(4-hydroxyphenethyl)-1,5-dihydro-2*H*-pyrrol-2-one (Compound 30)

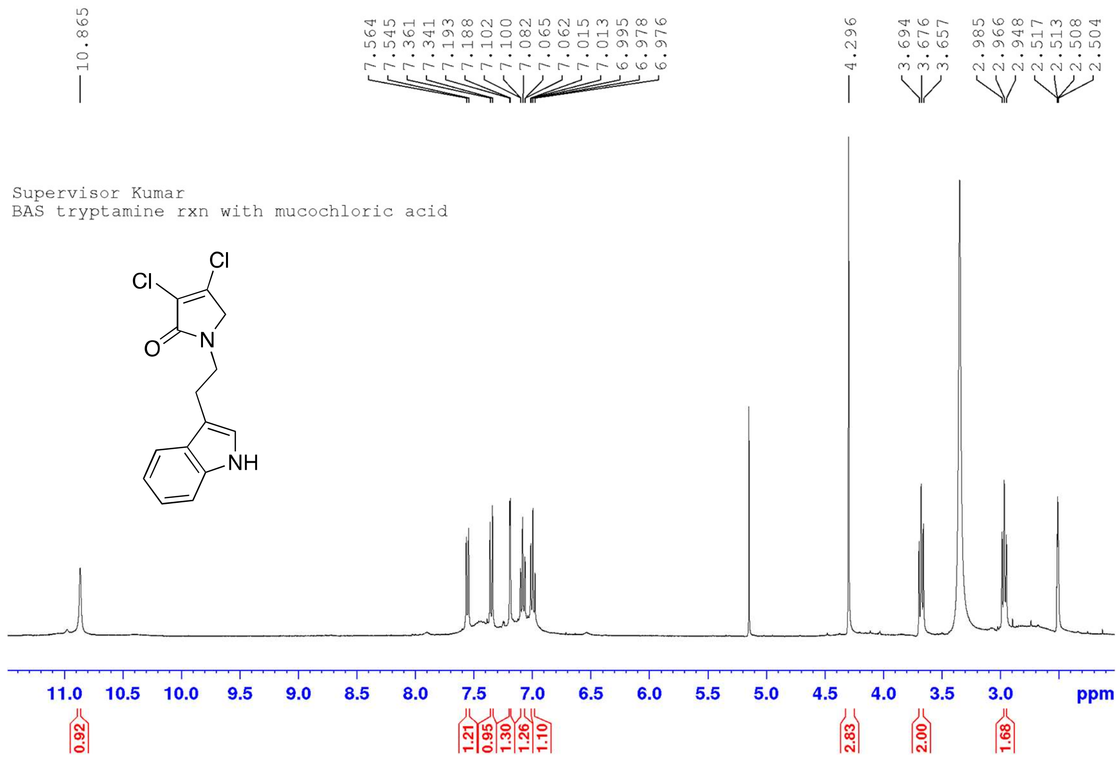

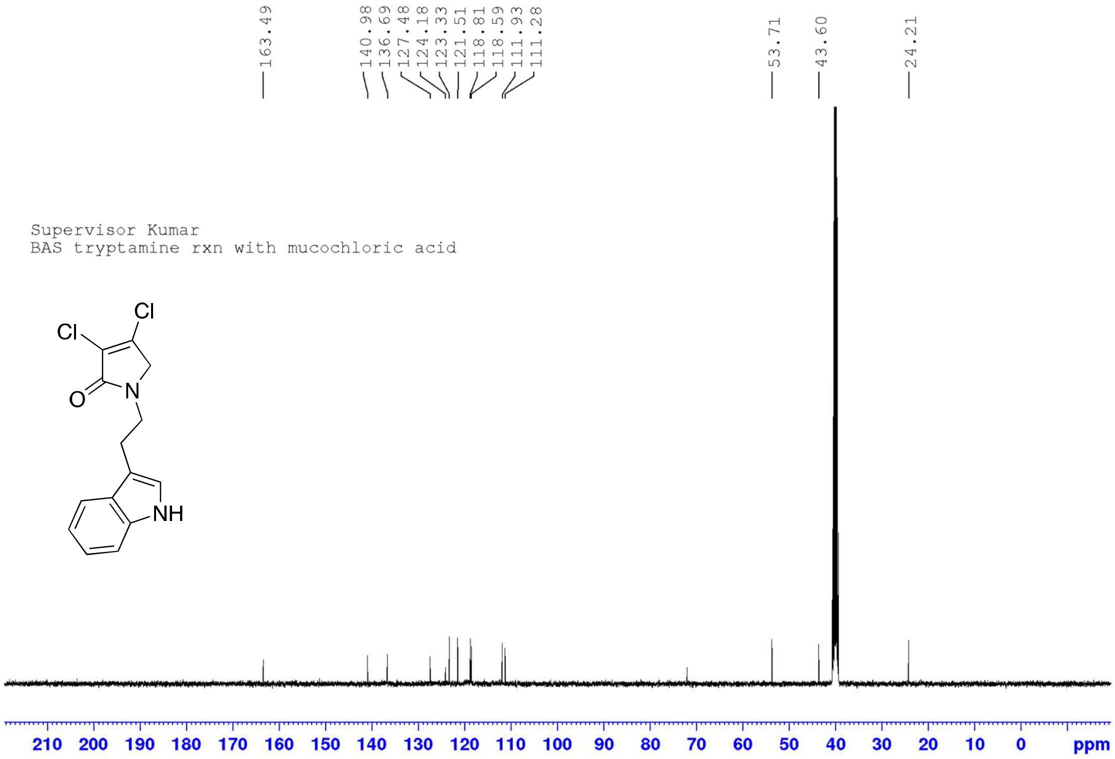

22) 1-(2-(1*H*-indol-3-yl)ethyl)-3,4-dichloro-1,5-dihydro-2*H*-pyrrol-2-one (Compound 31)

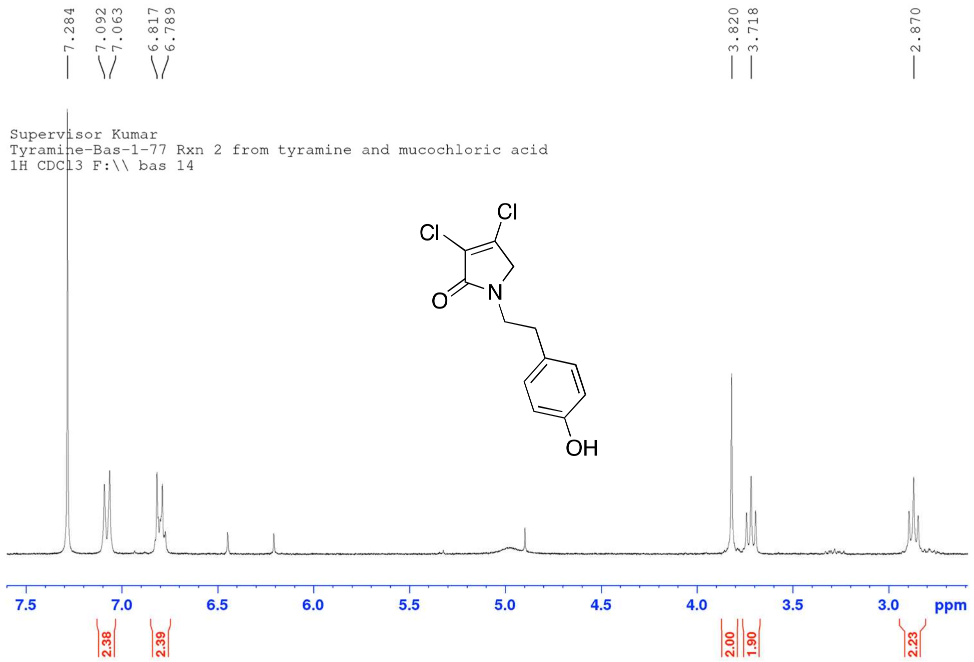

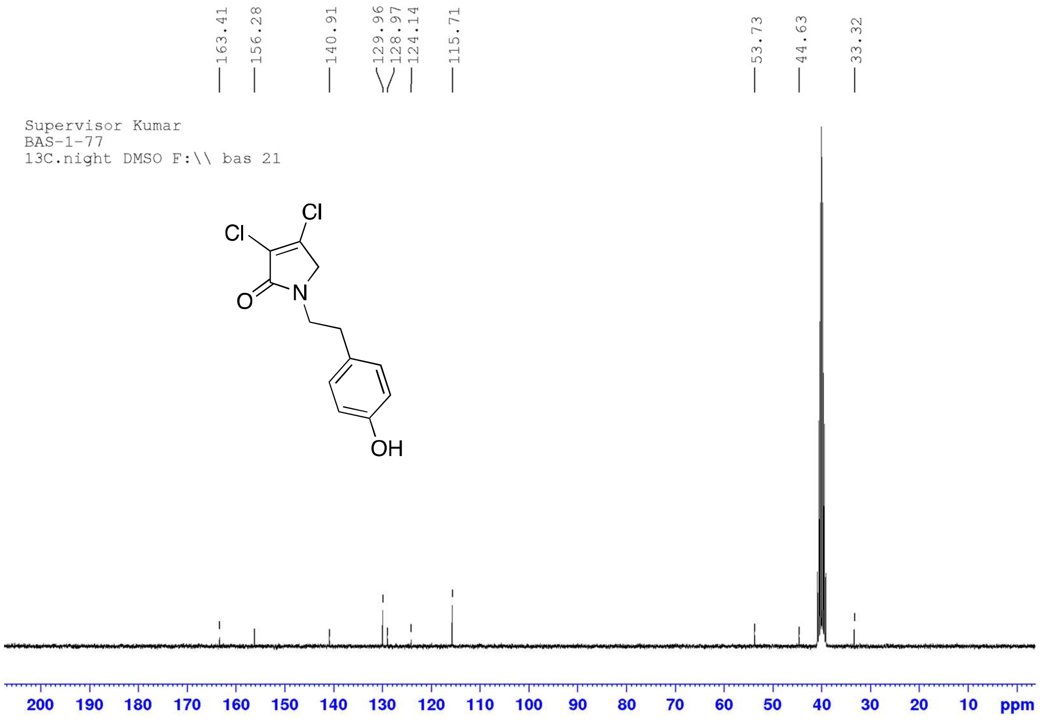

23) *N*-butyl-4-(3,4-dichloro-2-oxo-2,5-dihydro-1*H*-pyrrol-1-yl)benzamide (Compound 32)

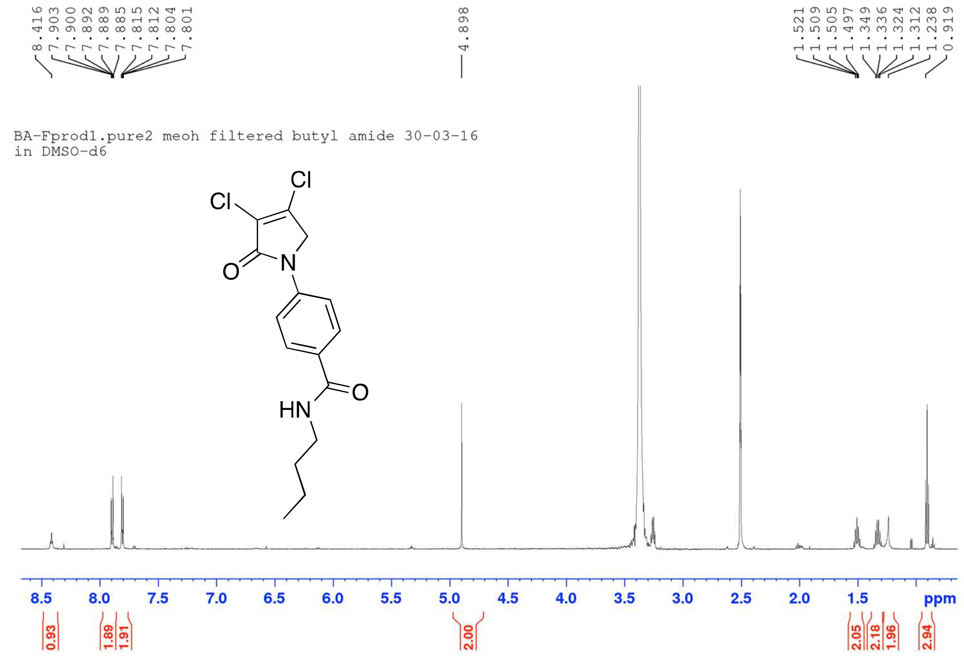

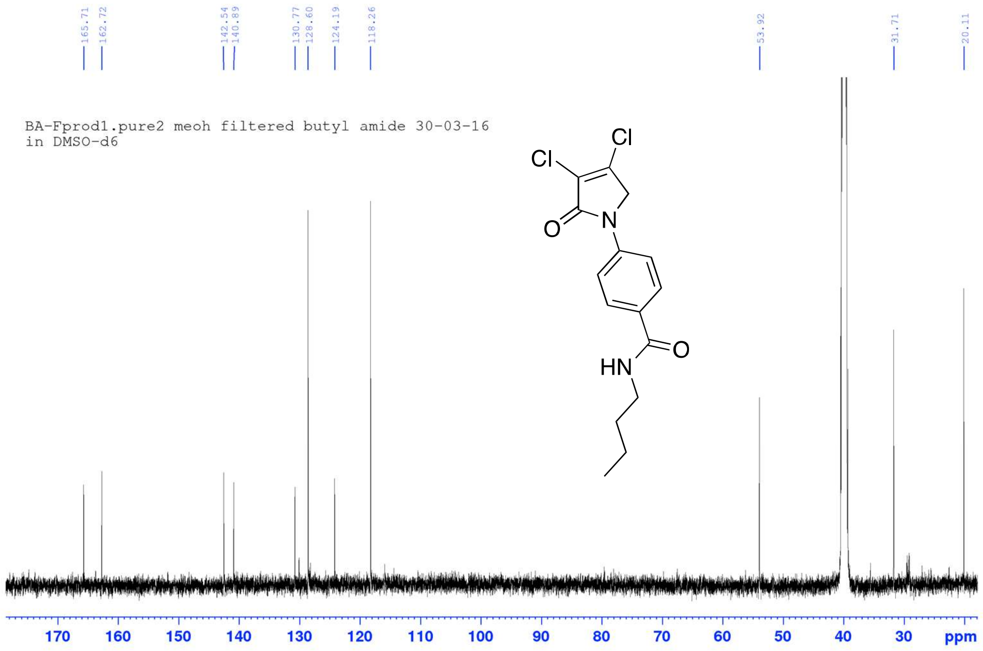

24) *N*-hexyl-4-(3,4-dichloro-2-oxo-2,5-dihydro-1*H*-pyrrol-1-yl)benzamide (Compound 33)

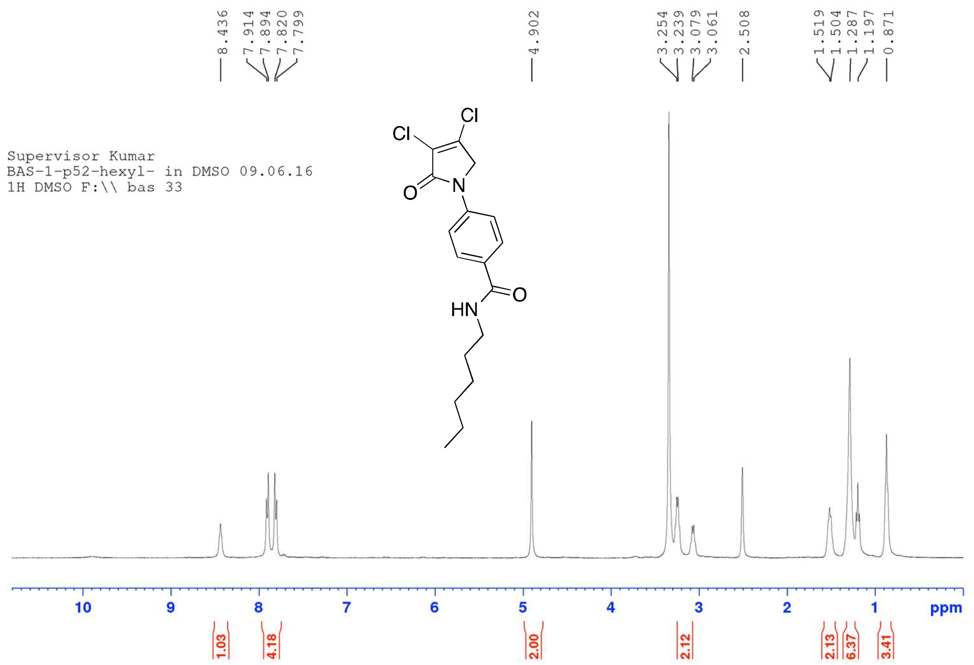

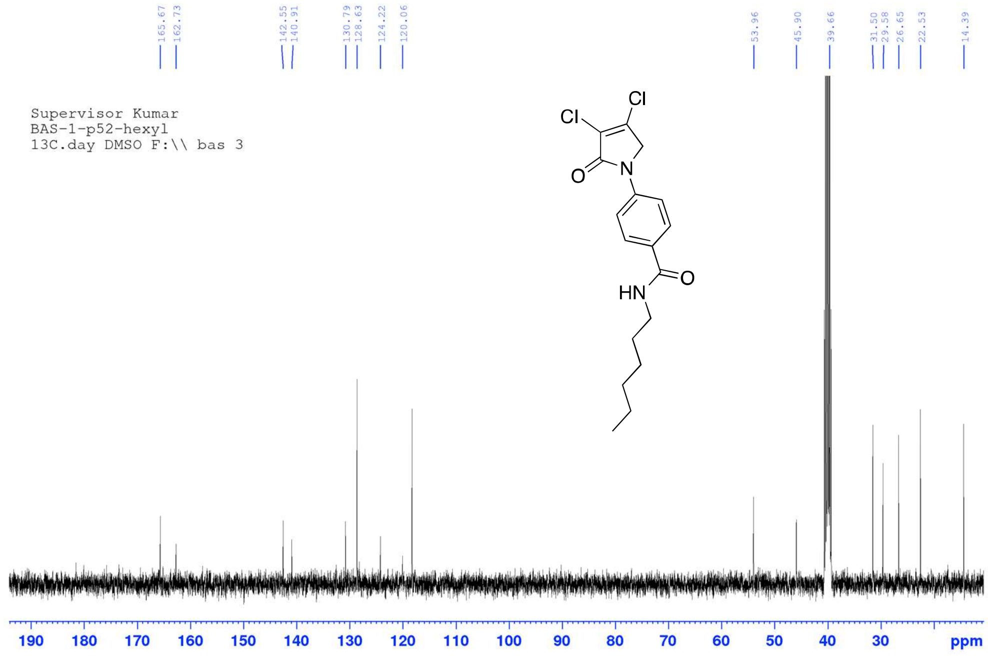

25) *N*-benzyl-4-(3,4-dichloro-2-oxo-2,5-dihydro-1*H*-pyrrol-1-yl)benzamide (Compound 34)

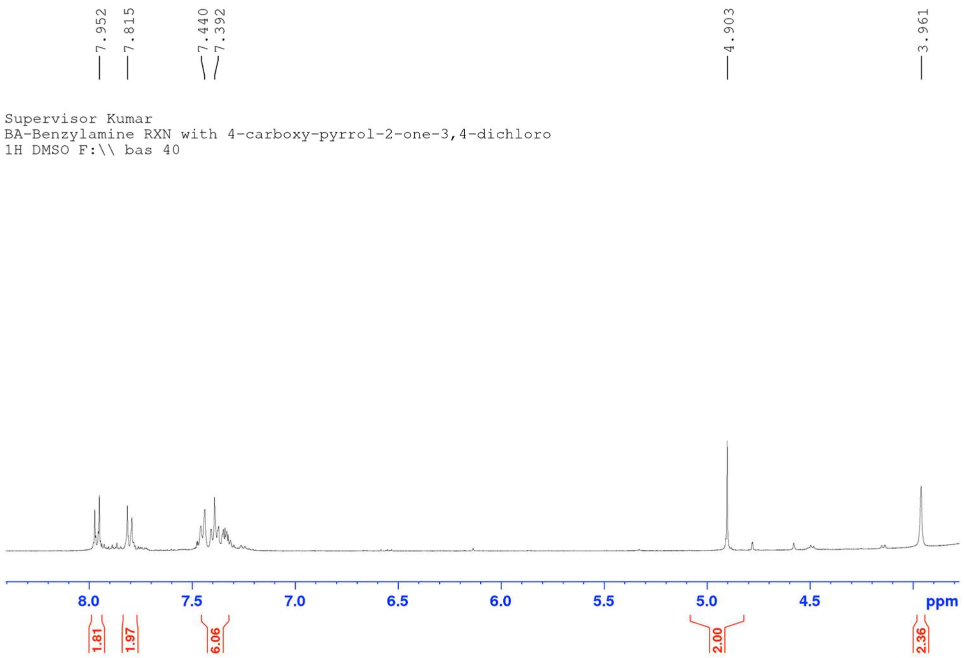

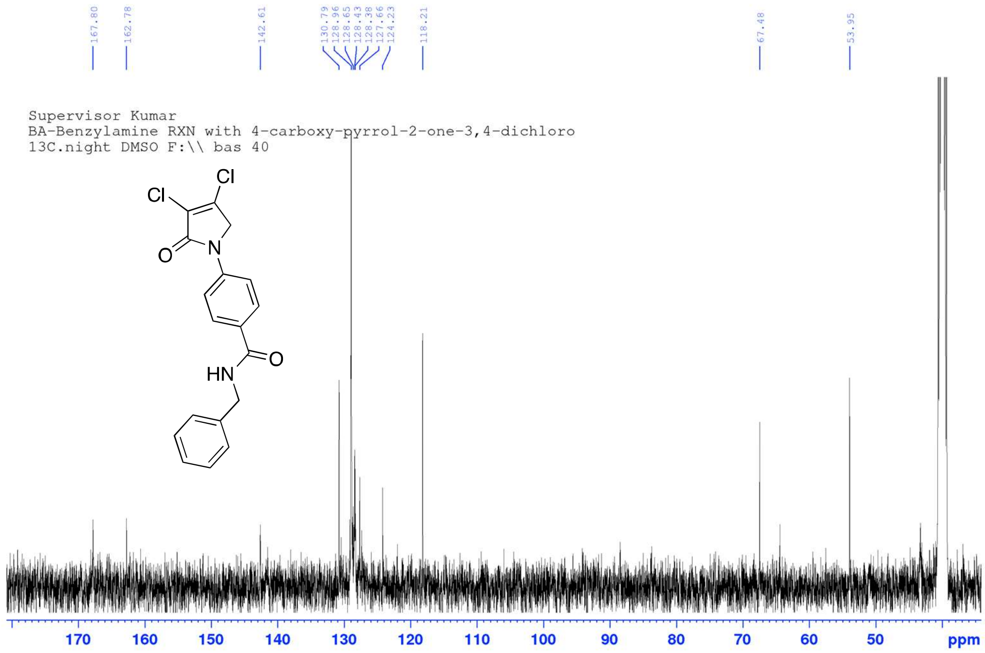

26) *N*-(2-(1*H*-indol-3-yl)ethyl)-4-(3,4-dichloro-2-oxo-2,5-dihydro-1*H*-pyrrol-1-yl)benzamide (Compound 35)

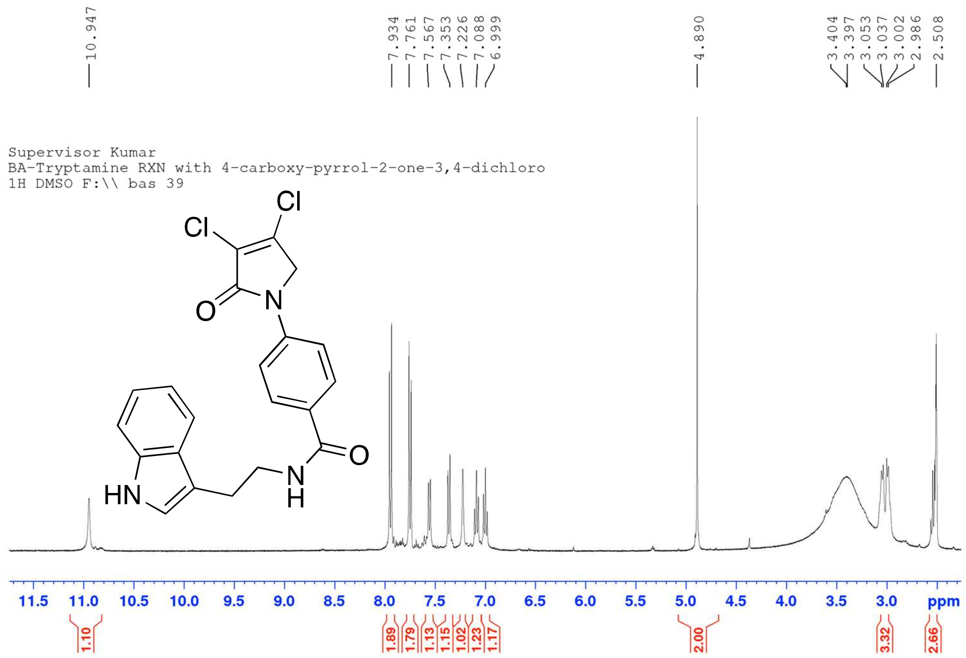

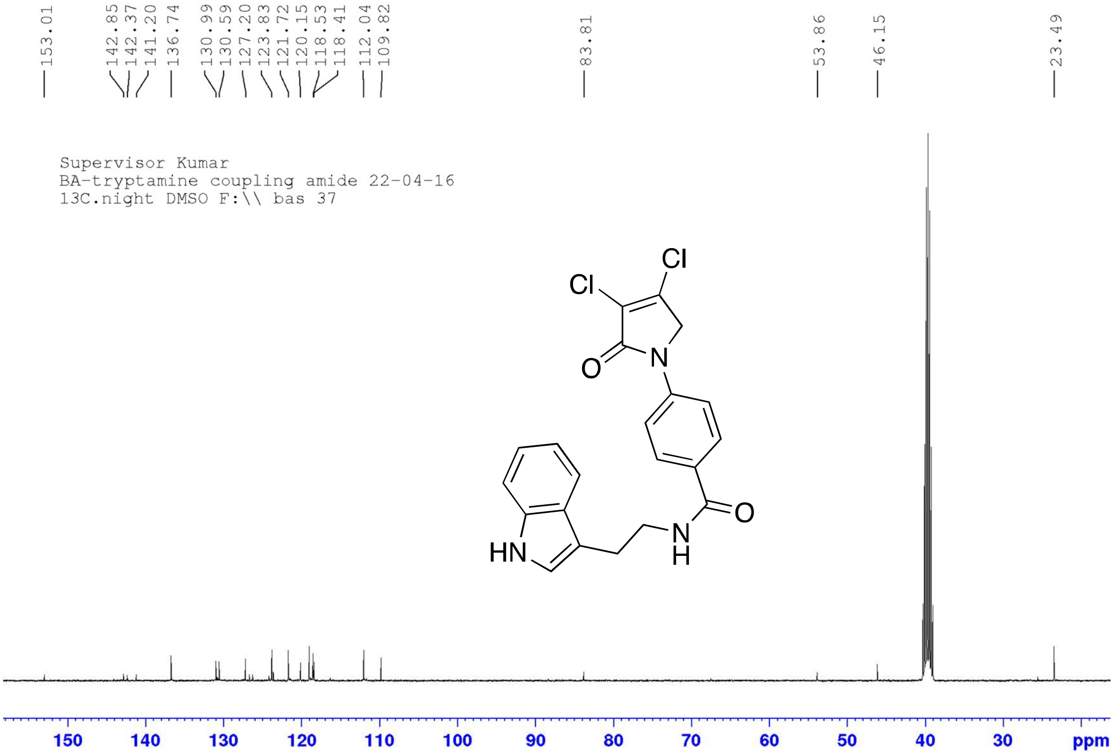

27) 4-(3,4-dichloro-2-oxo-2,5-dihydro-1*H*-pyrrol-1-yl)-*N*-(2-morpholinoethyl)benzamide (Compound 36)

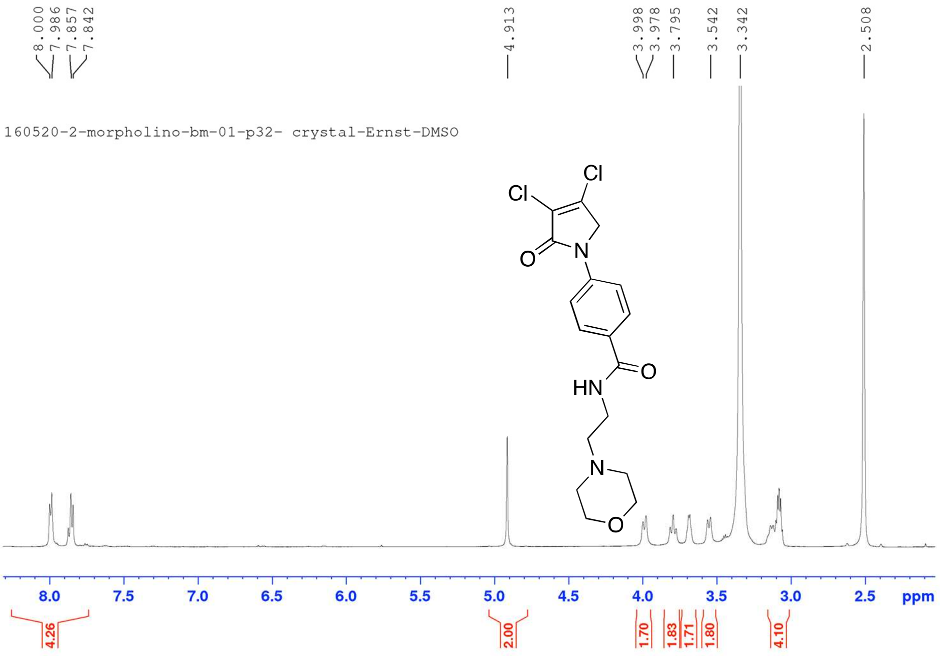

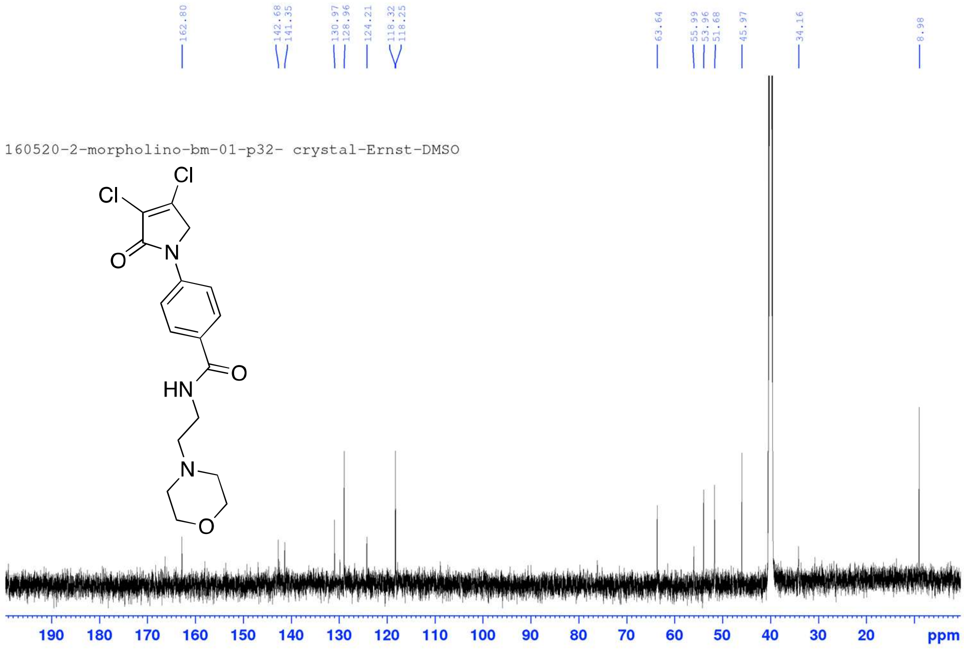

28) 4-(3,4-dichloro-2-oxo-2,5-dihydro-1*H*-pyrrol-1-yl)-*N*-(3-morpholinopropyl)benzamide (Compound 37)

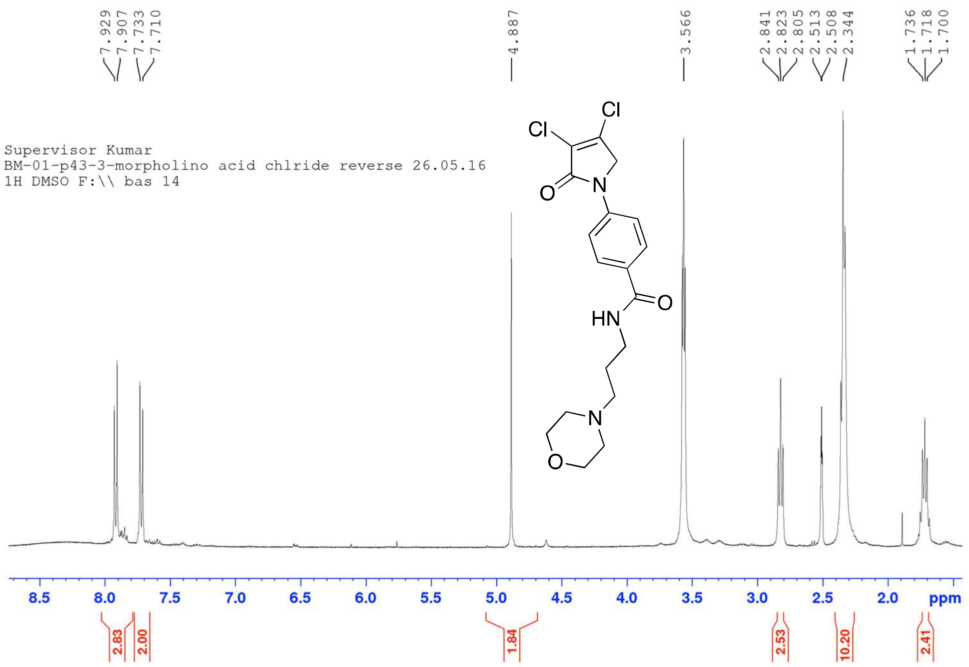

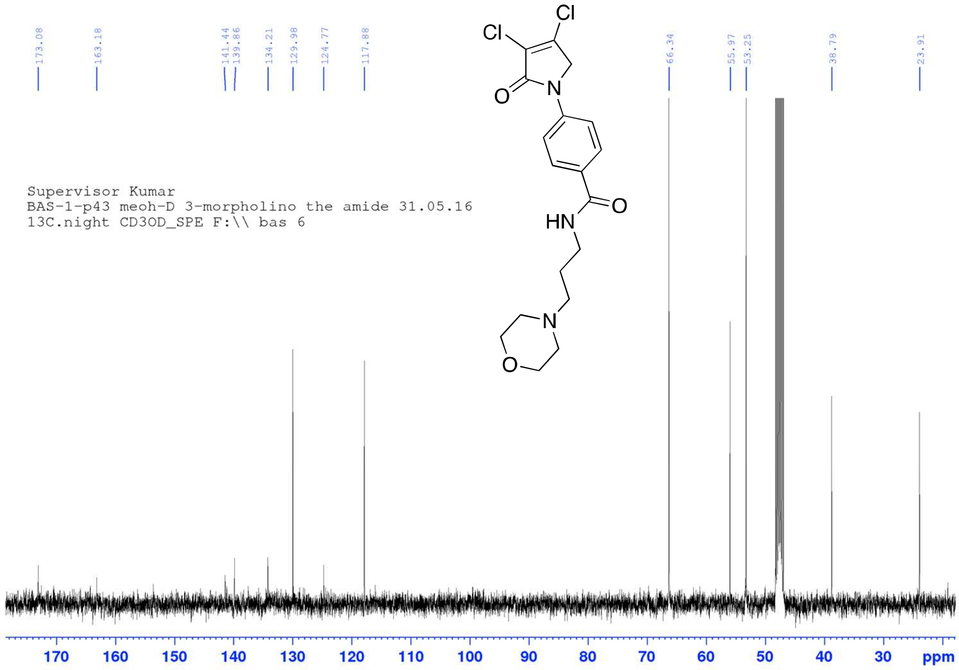

29) 4-(3,4-dichloro-2-oxo-2,5-dihydro-1*H*-pyrrol-1-yl)-*N*-(3-(piperidin-1-yl)propyl)benzamide (Compound 38)

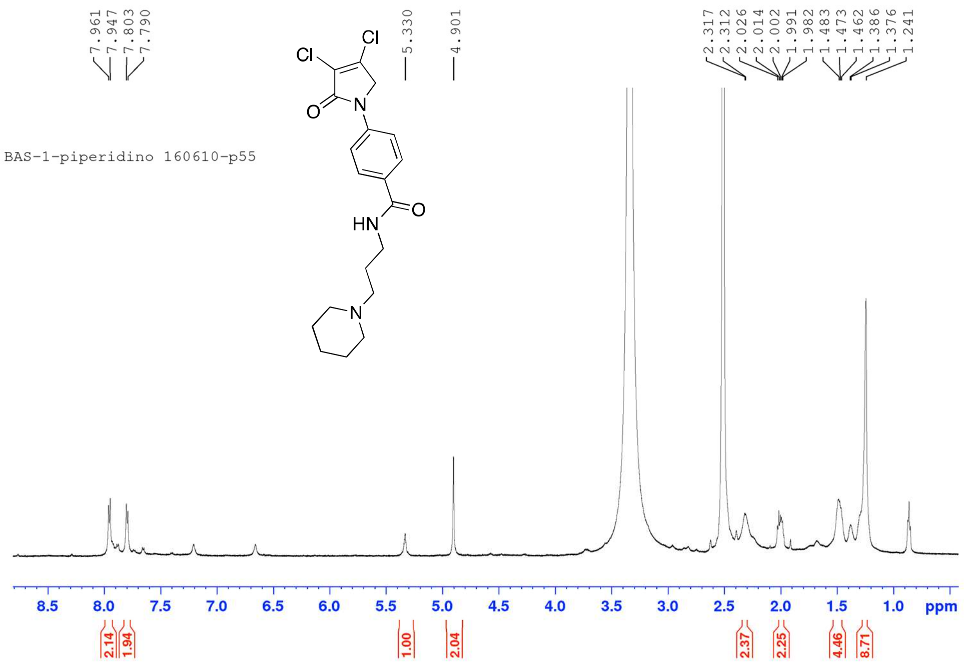

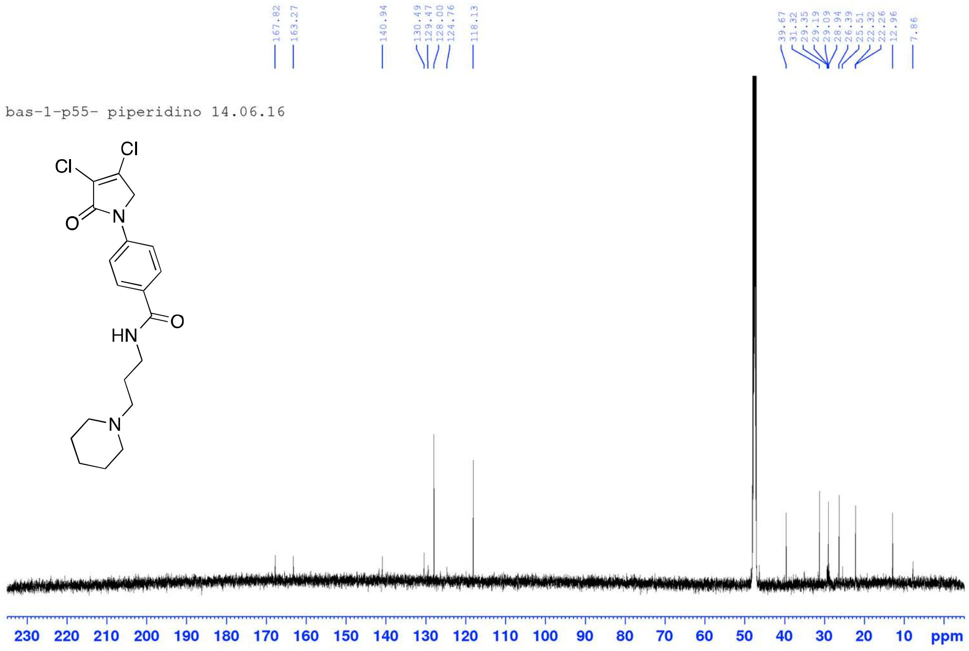

30) *N*-(4-(3,4-dichloro-2-oxo-2,5-dihydro-1*H*-pyrrol-1-yl)phenyl)butyramide (Compound 39)

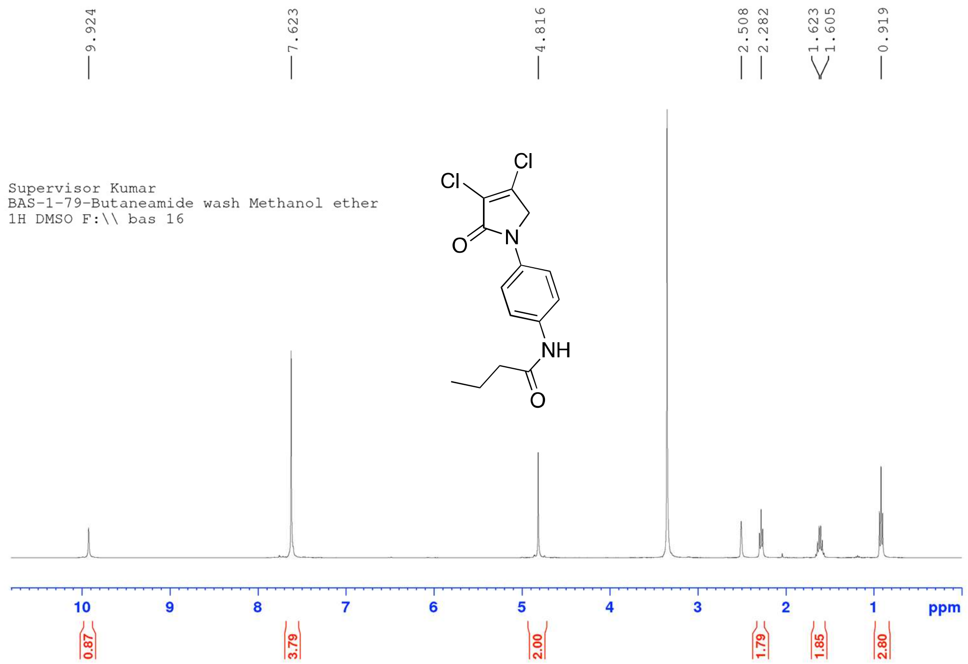

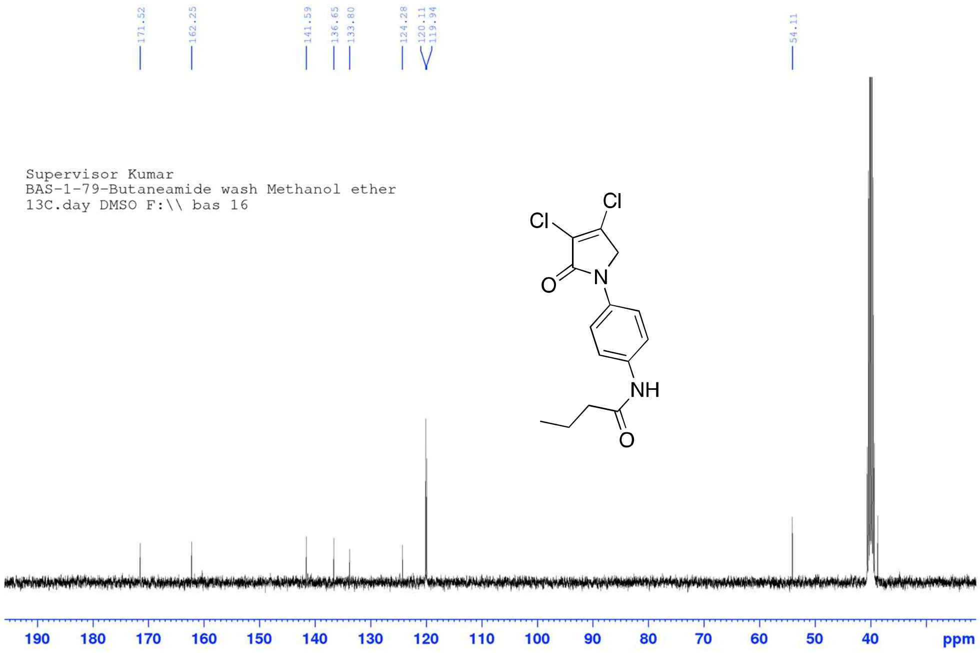

31) *N*-(4-(3,4-dichloro-2-oxo-2,5-dihydro-1*H*-pyrrol-1-yl)phenyl)hexanamide (Compound 40)

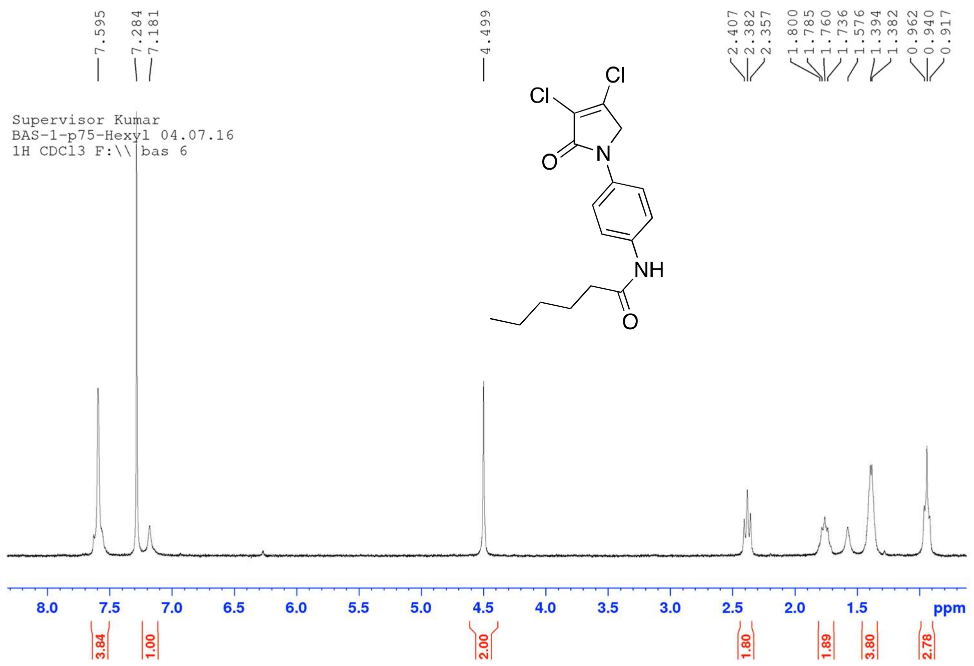

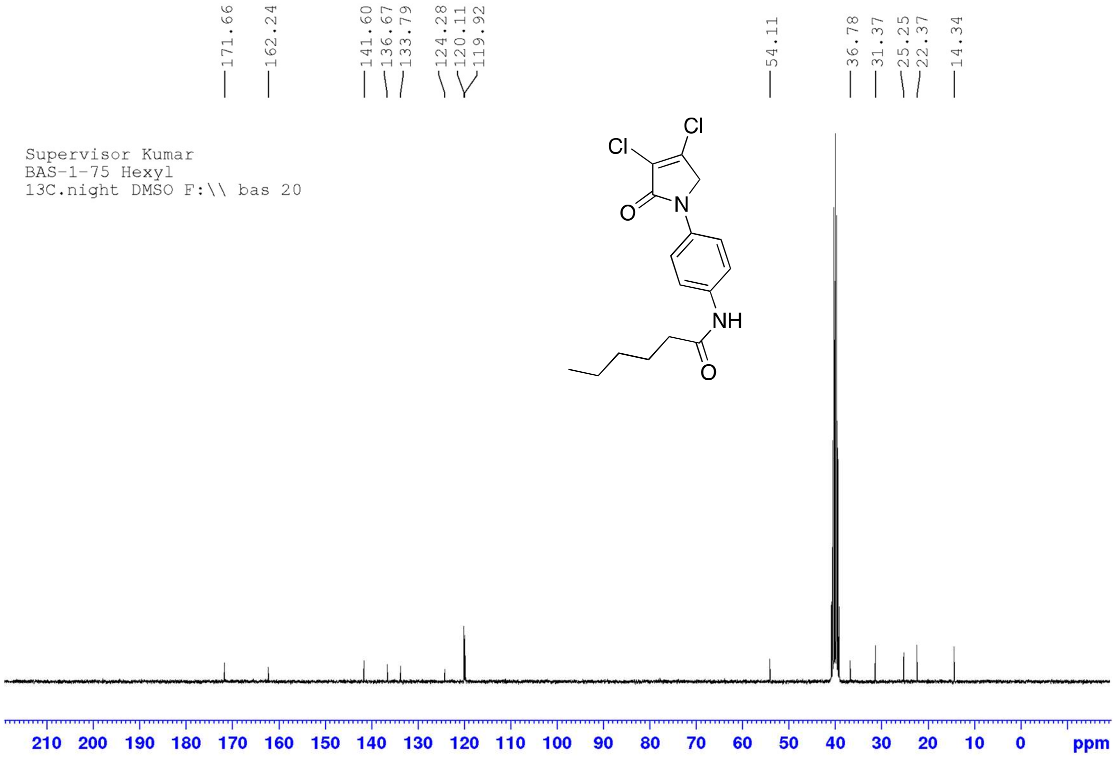

32) N-(4-(3,4-dichloro-2-oxo-2,5-dihydro-1H-pyrrol-1-yl)phenyl)octanamide (Compound 41)

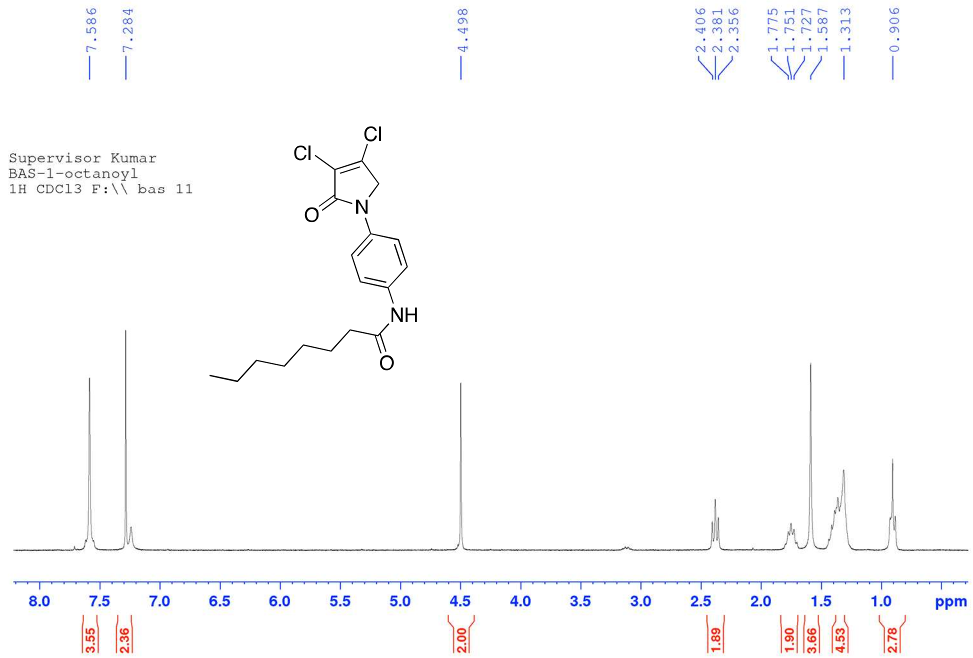

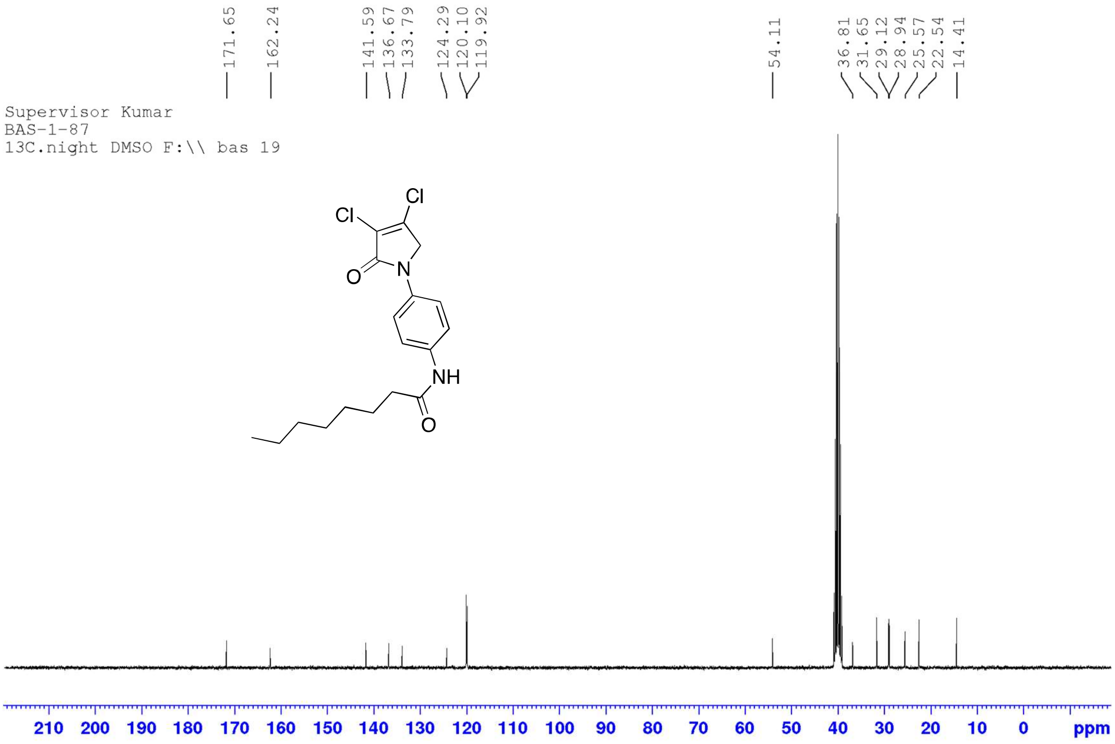

33) 2-(4-bromophenyl)-*N*-(4-(3,4-dichloro-2-oxo-2,5-dihydro-1*H*-pyrrol-1-yl)phenyl)acetamide (Compound 42)

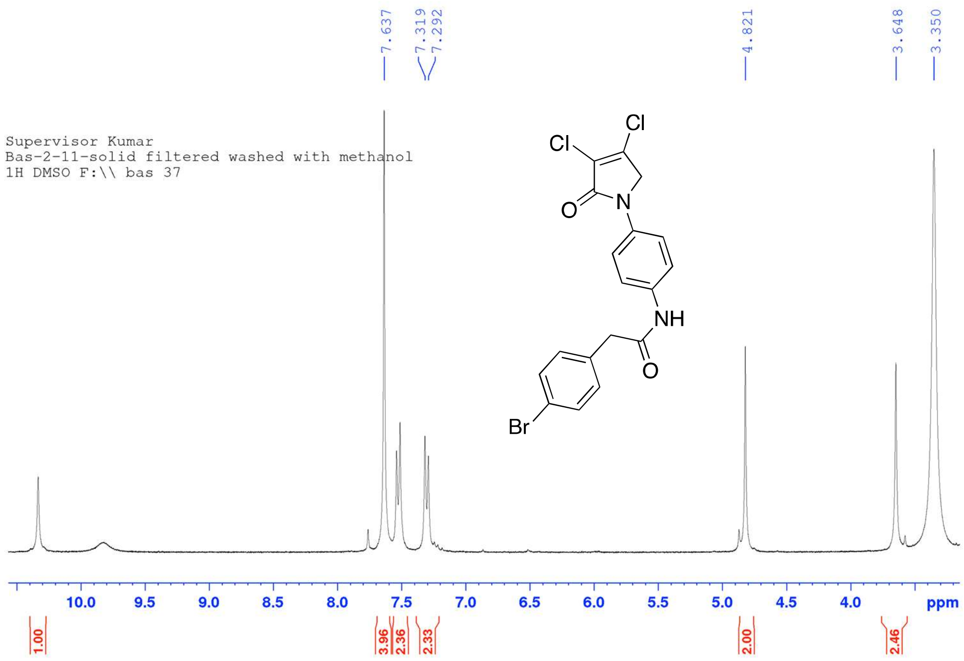

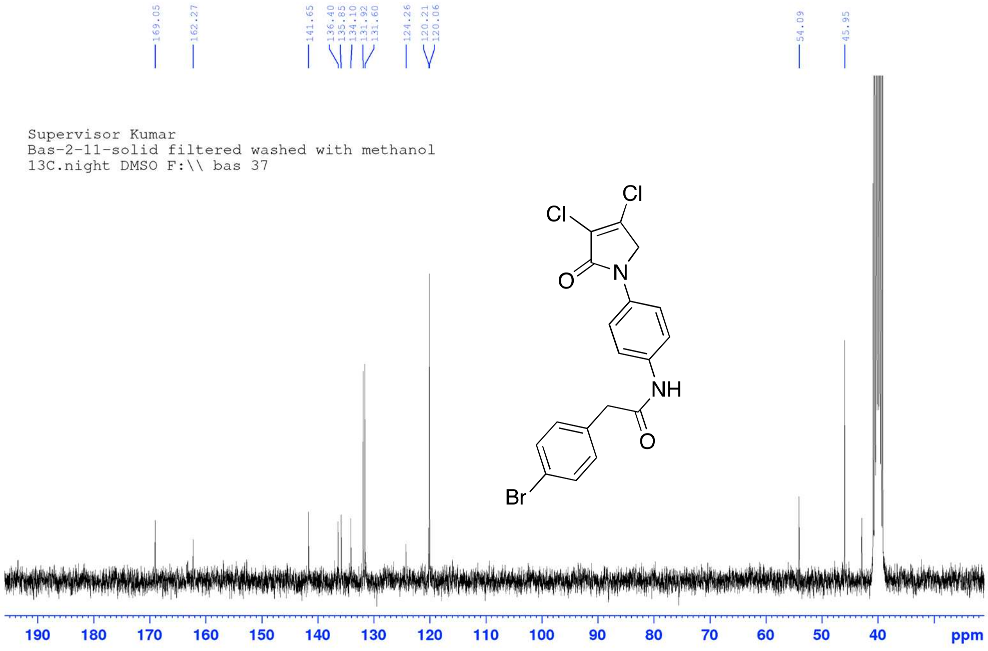

Supplement: Supplementary file 1 [file molecules-23-01106-s001.pdf]
